# Exploring the Subtle Effect of Aliphatic Ring Size on Minor Actinide‐Extraction Properties and Metal Ion Speciation in Bis‐1,2,4‐Triazine Ligands

**DOI:** 10.1002/chem.201903685

**Published:** 2019-10-30

**Authors:** Andrey V. Zaytsev, Rachel Bulmer, Valery N. Kozhevnikov, Mark Sims, Giuseppe Modolo, Andreas Wilden, Paul G. Waddell, Andreas Geist, Petra J. Panak, Patrik Wessling, Frank W. Lewis

**Affiliations:** ^1^ Department of Applied Sciences Faculty of Health and Life Sciences Northumbria University Newcastle upon Tyne NE1 8ST UK; ^2^ Forschungszentrum Jülich GmbH Institut für Energie und Klimaforschung—Nukleare Entsorgung und Reaktorsicherheit (IEK-6) 52428 Jülich Germany; ^3^ School of Natural and Environmental Sciences Newcastle University Kings Road Newcastle upon Tyne NE1 7RU UK; ^4^ Institute for Nuclear Waste Disposal (INE) Karlsruhe Institute of Technology (KIT) 76021 Karlsruhe Germany; ^5^ Ruprecht-Karls-Universität Heidelberg Physikalisch-Chemisches Institut Im Neuenheimer Feld 234 69120 Heidelberg Germany

**Keywords:** actinides, lanthanides, solvent extraction, spent nuclear fuel, triazine ligands

## Abstract

The synthesis and evaluation of three novel bis‐1,2,4‐triazine ligands containing five‐membered aliphatic rings are reported. Compared to the more hydrophobic ligands **1**–**3** containing six‐membered aliphatic rings, the distribution ratios for relevant *f*‐block metal ions were approximately one order of magnitude lower in each case. Ligand **10** showed an efficient, selective and rapid separation of Am^III^ and Cm^III^ from nitric acid. The speciation of the ligands with trivalent *f*‐block metal ions was probed using NMR titrations and competition experiments, time‐resolved laser fluorescence spectroscopy and X‐ray crystallography. While the tetradentate ligands **8** and **10** formed Ln^III^ complexes of the same stoichiometry as their more hydrophobic analogues **2** and **3**, significant differences in speciation were observed between the two classes of ligand, with a lower percentage of the extracted 1:2 complexes being formed for ligands **8** and **10**. The structures of the solid state 1:1 and 1:2 complexes formed by **8** and **10** with Y^III^, Lu^III^ and Pr^III^ are very similar to those formed by **2** and **3** with Ln^III^. Ligand **10** forms Cm^III^ and Eu^III^ 1:2 complexes that are thermodynamically less stable than those formed by ligand **3**, suggesting that less hydrophobic ligands form less stable An^III^ complexes. Thus, it has been shown for the first time how tuning the cyclic aliphatic part of these ligands leads to subtle changes in their metal ion speciation, complex stability and metal extraction affinity.

## Graphical abstract

**Calling all actinides**! Bis‐1,2,4‐triazine ligands bearing five‐membered rings were synthesized and evaluated as actinide‐selective extractants. Tuning the size of the aliphatic ring leads to subtle changes in actinide‐extraction properties. The origins of these changes were elucidated at the molecular level, paving the way for the rational design of improved actinide‐selective extractants for reprocessing of spent nuclear fuel.,

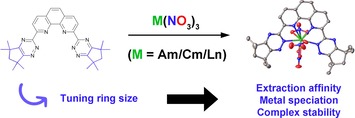

## Introduction

Nuclear energy offers a clean, low carbon source of electricity that is becoming a growing part of the energy mix in many countries worldwide. However, the spent fuel that is produced in nuclear fission reactors is long‐lived and highly radiotoxic.^1^ Following reprocessing to remove uranium and plutonium, the minor actinides americium, curium and neptunium are responsible for much of the long‐term heat load and radiotoxicity of the remaining spent fuel material. Removing these elements before disposal would contribute to sustainable nuclear energy by significantly reducing the size of the final waste repository, and the time needed for the remaining material to decay to the radiotoxicity level of natural uranium (from ca. 10^4^ years to a few hundred years).^2^ Beyond the currently used PUREX process that recovers and recycles most of the uranium and plutonium,^3^ future reprocessing scenarios seek to close the nuclear fuel cycle by separating (partitioning) the minor actinides from the chemically similar and less‐radiotoxic lanthanides, prior to their burning (transmutation) in high neutron flux advanced fast reactors or in accelerator‐driven systems.^4^

**Figure 1:**
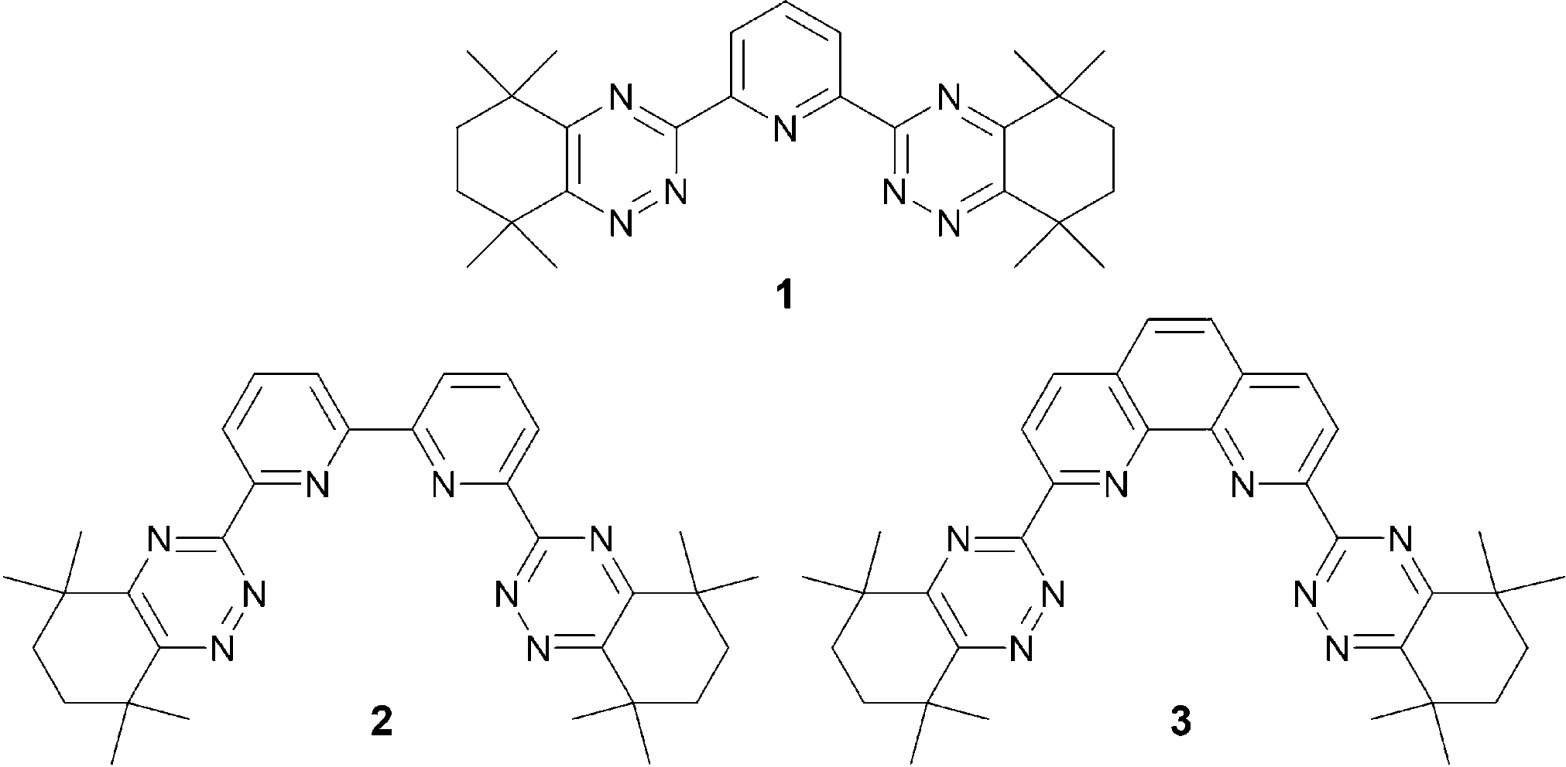
Structures of the benchmark bis‐1,2,4‐triazine ligands **1**, **2** and **3** containing six‐membered aliphatic rings.

Numerous soft N‐ and S‐donor ligands have been evaluated to accomplish the challenging separation of the minor actinides from the lanthanides in a future solvent extraction process.^5^, ^6^ The greater orbital overlap between the more radially extended 5*f* orbitals of the actinides and ligand lone pairs is thought to be the basis for this separation.^7^ Among N‐donor ligands, bis‐1,2,4‐triazine ligands **1**–**3** (Figure 1) fulfil most of the challenging criteria to date for use in such a process. In particular, bis‐triazinyl‐phenanthroline ligands such as **3** 8 and its derivatives have been extensively investigated.^9^ Recent research has focused mostly on the effects that substituents attached to the aromatic rings of **2** 10 and **3** 11 have on their extraction properties. However, there has been less emphasis on modifying the aliphatic rings appended to the triazine rings of ligands **1**–**3**.^12^ We wished to determine what effect changing the aliphatic ring size would have on the actinide extraction properties and metal speciation of these ligands. In this paper, we report our studies on novel bis‐1,2,4‐triazine ligands containing a five‐membered aliphatic ring appended to the outer triazine rings instead of a six‐membered ring (as in **1**–**3**), and we show that this small but subtle modification to the ligand structure can have unexpected effects on the extraction properties and metal ion speciation of these ligands.

## Results and Discussion

### Synthesis and solvent‐extraction studies

**Scheme 1:**
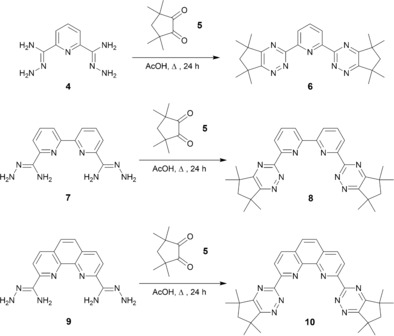
Synthesis of bis‐1,2,4‐triazine ligands **6**, **8** and **10** containing five‐membered aliphatic rings from the α‐diketone **5**.

The novel bis‐1,2,4‐triazine ligands **6**, **8** and **10** were synthesized in moderate to high yields as shown in Scheme 1. The α‐diketone **5** was synthesized by the oxidation of 2,2,4,4‐tetramethylcyclopentanone with selenium(IV) oxide as previously described.^13^ The condensation reaction of **5** with the known bis‐amidrazone **4** ^[14, 15**]**^ in refluxing acetic acid afforded the novel terdentate ligand **6** in 75 % yield. Similarly, the novel tetradentate ligand **8** was obtained from the known bis‐amidrazone **7** 14, 15 in 59 % yield, and the novel tetradentate ligand **10** was obtained from the known bis‐amidrazone **9** 8 in 84 % yield (Scheme 1).

Preliminary solvent extraction experiments were then carried out to determine the ability of the ligands **6**, **8** and **10** to extract An^III^ and separate them from Ln^III^. The distribution ratios (*D*) for Am^III^ and Eu^III^, and the separation factors (SF_Am/Eu_) for the extraction of Am^III^ and Eu^III^ from nitric acid by solutions of terdentate ligand **6** in 1‐octanol are presented in the Supporting Information (section 4.1). The distribution ratios for Am^III^ increased with increasing nitric acid concentration to a maximum *D*_Am_ value of 1.57 at 3.1 m HNO_3_, which corresponds to 61 % Am^III^ extraction. Although these *D* values are rather low, they would be sufficient for use in a multi‐step, counter‐current An^III^ extraction process depending on the conditions (number of stages, flow rates, etc). The average separation factor for Am^III^ over Eu^III^ was approximately 10 between 0.1 m and 1 m HNO_3_ and reached a maximum value at 3.1 m HNO_3_. The distribution ratios for Cm^III^ were very similar, and no significant selectivity for Am^III^ over Cm^III^ was observed for **6** (see Supporting Information section 4.1). The maximum *D*_Am_ value observed for **6** is slightly less than that reported previously for ligand **1** in 1‐octanol (*D*_Am_=3.9, 0.5 m HNO_3_, contact time=60 minutes).^16^ This is probably because ligand **6** is slightly less hydrophobic than ligand **1**, and thus forms less hydrophobic complexes.

Results for the extraction of Am^III^ and Eu^III^ by tetradentate ligand **8** at different nitric acid concentrations are presented in the Supporting Information (section 4.2). Extraction of Am^III^ and Eu^III^ by **8** showed a similar trend to that of ligand **2**, with the *D* values for both metals increasing as [HNO_3_] increases. With ligand **8**, a more efficient and selective extraction of Am^III^ was observed at high nitric acid concentrations than with ligand **6**. The selectivity of **8** for Am^III^ over Eu^III^ was significantly higher than that of ligand **6**, and the average separation factor was approx. 100 between 0.1 m and 3 m HNO_3_. Once again, no significant selectivity for Am^III^ over Cm^III^ was observed with **8** (see Supporting Information section 4.2). Interestingly, the *D* values for Am^III^ and Eu^III^ for **8** were approximately an order of magnitude lower than those previously reported for the more hydrophobic ligand **2** under similar conditions.^17^ The results cannot be directly compared however, as an additional co‐extractant; *N*,*N*′‐dimethyl‐*N*,*N*′‐dioctyl‐2‐hexyloxyethyl malonamide **11**, was used in the case of **2**. To allow a direct comparison with **2**, we carried out extraction experiments for **8** in 1‐octanol in the presence of 0.25 m **11** (see Supporting Information section 4.2). This led to a slight increase in the *D* values for Am^III^ at ≥1 m HNO_3_, but a marked decrease in the selectivity for Am^III^ over Eu^III^ (SF_Am/Eu_≤57 at 1–4 m HNO_3_) compared to the results in the absence of **11**. This is due to the competing non‐selective co‐extraction of Am^III^ and Eu^III^ by **11**, which lowers the separation factor. However, the distribution ratios for Am^III^ and Eu^III^ were still significantly lower with **8** than with **2**.^17^

**Figure 2:**
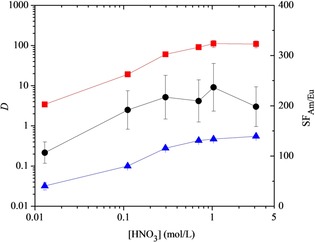
Extraction of Am^III^ and Eu^III^ by tetradentate ligand **10** in 1‐octanol (0.01 m) as a function of the initial nitric acid concentration (*D*=distribution ratio, SF=separation factor, ▪=*D*_Am_, ▴=*D*_Eu_, •=SF_Am/Eu_, mixing time: 60 min., temperature: 22 °C ±1 °C).

Results for the extraction of Am^III^ and Eu^III^ by tetradentate ligand **10** at different nitric acid concentrations are presented in Figure 2. A highly efficient and selective extraction of Am^III^ over Eu^III^ was observed across a range of nitric acid concentrations. The *D* values for Am^III^ reached a maximum value of 112 at 1 m HNO_3_. A maximum selectivity for Am^III^ over Eu^III^ was also observed at 1 m HNO_3_ (SF_Am/Eu_=237). The selectivity for Am^III^ over Eu^III^ shown by ligand **10** was similar to that shown by the analogous, more hydrophobic ligand **3**.^8^ However, the *D* values for both Am^III^ and Eu^III^ were approximately an order of magnitude lower with ligand **10** at high acidity than with ligand **3** (*D*_Am_≈1000, *D*_Eu_≈5 for **3** at ≥1 m HNO_3_; *D*_Am_≈100, *D*_Eu_≈0.5 for **10** at ≥1 m HNO_3_). This could allow for easier back‐extraction (stripping) of the metals from the loaded organic phase after the extraction stages have been carried out. Ligand **10** did not show any significant selectivity for Am^III^ over Cm^III^ (SF_Am/Cm_≤2.2, see Supporting Information section 4.3), in contrast to ligand **3**.^18^

**Figure 3:**
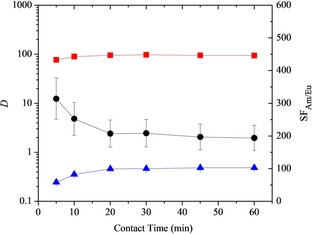
Extraction of Am^III^ and Eu^III^ from 1.03 m nitric acid by tetradentate ligand **10** (0.01 m) in 1‐octanol as a function of contact time (*D*=distribution ratio, SF=separation factor, ▪=*D*_Am_, ▴=*D*_Eu_, ▪=SF_Am/Eu_, temperature: 22 °C ±1 °C).

The extraction of Am^III^ and Eu^III^ by ligand **10** as a function of contact time is presented in Figure 3. As shown, Am^III^ extraction equilibrium was reached within 10 minutes of phase mixing, while Eu^III^ extraction equilibrium was reached after a mixing time of 20 minutes. Thus, the rates of metal extraction were slightly faster for the less hydrophobic ligand **10** than for its more hydrophobic analogue **3** under the same conditions (15 minutes for *D*_Am_ and ≥60 minutes for *D*_Eu_ to reach equilibrium for **3**).^8^

Spent nuclear fuel solutions contain large amounts of Y^III^ and light trivalent lanthanides, which must be separated from the minor actinides. We therefore measured the distribution ratios for Y^III^ and all lanthanides (except Pm^III^) as well as the trivalent actinides. For all three ligands **6**, **8** and **10**, the *D* values for the lanthanides showed an extraction profile across the lanthanide series of first increasing, then decreasing *D* values, in agreement with previous results for ligands **2** and **3**.^8^, ^17^ In the extractions from 3.1 m HNO_3_, Ho^III^ exhibited the highest *D* values for ligands **8** and **10** (*D*_Ho_=0.37 for **8**, *D*_Ho_=3.56 for **10**), while Dy^III^ exhibited the highest *D* value for ligand **6** (*D*_Dy_=0.08). Thus a practical separation of Am^III^ and Cm^III^ from all the lanthanides could be feasible with ligands **6** and **8** (*D*_Ln_<1). Although the later lanthanides Tb^III^–Yb^III^ are somewhat extracted by ligand **10** at high nitric acid concentrations, a highly selective separation of Am^III^ and Cm^III^ from all the lanthanides is feasible since selective lanthanide back‐extraction can be carried out at lower nitric acid concentrations (*D*_Am_ and *D*_Cm_>1, *D*_Ln_<1 at 0.1 m HNO_3_). Furthermore, the later lanthanides are not present in spent fuel solutions, so their extraction is less relevant than that of the early lanthanides.

### NMR titrations and X‐ray crystallography

To gain further insight into the solution speciation of these ligands with metal ions and to rationalise the extraction results, we carried out some ^1^H NMR titrations of the ligands with Y^III^ and the diamagnetic lanthanides La^III^ and Lu^III^. We have previously employed this method to investigate the solution speciation of the analogous ligands **2** and **3**, and related tetradentate ligands with trivalent lanthanides.10a, 19, 20 We used deuterated acetonitrile due to the high cost of deuterated 1‐octanol and to compare with previous results for **2** and **3**.^19^

**Figure 4:**
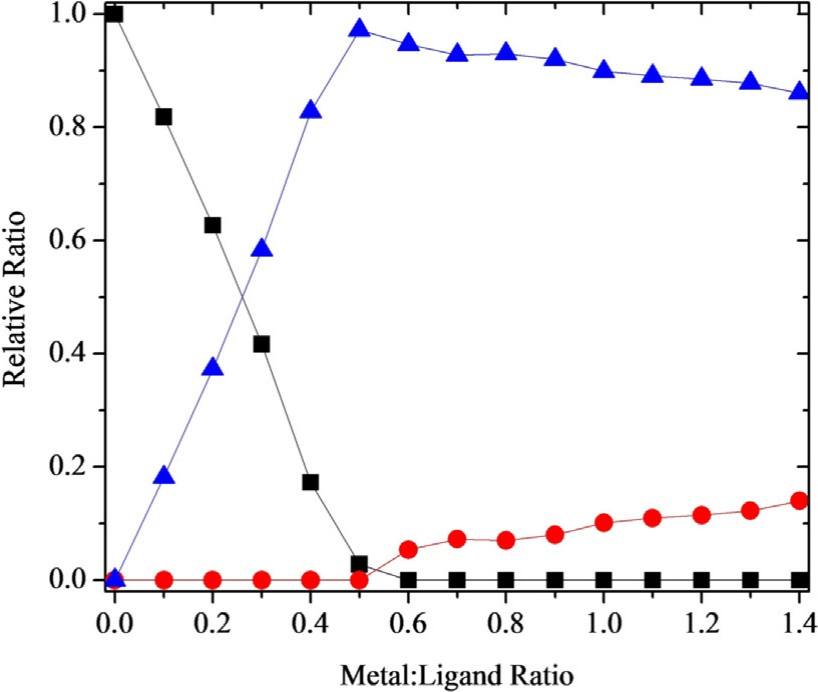
^1^H NMR titration of tetradentate ligand **8** with Y(NO_3_)_3_ in CD_3_CN (Key: ▪=free ligand, •=1:1 complex, ▴=1:2 complex).

For tetradentate ligand **8**, both 1:1 and 1:2 m:L species were observed during the ^1^H NMR titration with Y(NO_3_)_3_ in deuterated acetonitrile. A single species was observed initially during the titration, and the disappearance of the free ligand resonances at a metal:ligand ratio of 0.5 indicates this was the 1:2 species [Y(**8**)_2_(NO_3_)]^2+^. Small amounts of the charge neutral 1:1 complex [Y(**8**)(NO_3_)_3_] were observed at higher metal:ligand ratios, reaching a maximum of 14 %. This complex is formed by partial dissociation of the 1:2 complex. The species distribution curve for the titration of ligand **8** with Y(NO_3_)_3_ is shown in Figure 4. The NMR stack plot is shown in the Supporting Information (section 5.2).

Similar results were observed for the ^1^H NMR titrations of ligand **10** with La^III^, Lu^III^ and Y^III^ (see Supporting Information section 5.3). Both 1:1 and 1:2 m:L species were observed during the ^1^H NMR titration of **10** with La(NO_3_)_3_ in deuterated acetonitrile. The 1:2 complexes [M(**10**)_2_(NO_3_)]^2+^ (M=La^III^, Lu^III^ or Y^III^) were observed initially and small amounts of the charge neutral 1:1 complexes [M(**10**)(NO_3_)_3_] were observed at higher metal:ligand ratios, reaching a maximum of 35, 30 and 18 % for La^III^, Lu^III^ and Y^III^, respectively. Although these results are broadly in agreement with those reported previously for tetradentate ligands **2** and **3**,^19^ a notable difference is observed in the relative ratios of the 1:1 and 1:2 complexes formed in each case. These differences are summarized in Table 1. For the present ligands **8** and **10**, the percentage of the 1:1 complex [M(L)(NO_3_)_3_] for a given metal ion is significantly higher than that observed for ligands **2** and **3** (36 % for **10** versus 27 % for **3** with La^III^, 18 % for **10** versus 5 % for **3** with Y^III^). Since it is known that the extracted species is the more hydrophobic 1:2 complex [M(L)_2_(NO_3_)]^2+^ (L=ligand), this could suggest that the lower distribution ratios observed above for ligands **8** and **10** in comparison to the analogous ligands **2** and **3** could be due to the lower percentage of 1:2 complexes being formed by these ligands under extraction conditions.

**Table 1:** Comparison of the species distribution of ligands **2** and **3** with ligands **8** and **10**.

Ligand	Metal	1:1 Species	1:2 Species	Ref.
10	La^III^	36 %	64 %	this work
3	La^III^	27 %	73 %	19
10	Lu^III^	30 %	70 %	this work
3	Lu^III^	21 %	79 %	19
10	Y^III^	18 %	82 %	this work
3	Y^III^	5 %	95 %	19
8	Y^III^	14 %	86 %	this work
2	Y^III^	7 %	93 %	19

During the ^1^H NMR titration of terdentate ligand **6** with Y(NO_3_)_3_ in deuterated acetonitrile, a single complex species was observed initially (see Supporting Information section 5.1). The complete disappearance of the free ligand resonances at a metal:ligand ratio of between 0.3 and 0.4 suggests that this is the expected 1:3 m:L complex [Y(**6**)_3_]^3+^. These 1:3 complexes are the major solution species formed by terdentate bis‐1,2,4‐triazine ligands with trivalent lanthanides.^16^, ^21^ Further evidence for the formation of this chiral racemic 1:3 complex, which exists as a pair of Λ and Δ enantiomers, was the appearance of four 6‐proton singlets in the aliphatic region corresponding to the four sets of diastereotopic methyl groups. Minor traces (≤10 %) of a second species were also observed on continued addition of metal. This was tentatively assigned as the 1:2 species, formed by partial dissociation of the 1:3 species.

**Figure 5:**
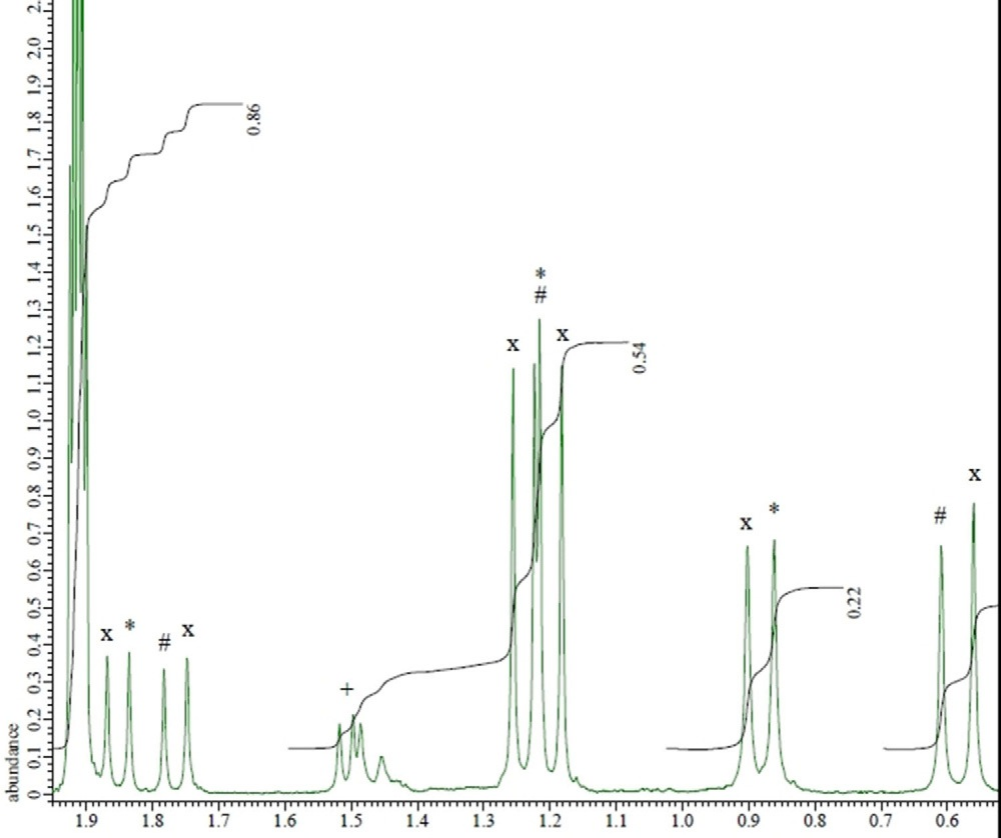
Aliphatic region of the ^1^H NMR spectrum of a 1:1:1 mixture of ligand **8**, ligand **10** and La(NO_3_)_3_ in CD_3_CN (Assignments: *=1:2 bis‐**8** complex, #=1:2 bis‐**10** complex, *x*=heteroleptic 1:2 bis‐complex, +=1:1 complex of **10**). Peak at 1.91 ppm is due to solvent.

A series of ^1^H NMR competition experiments were then carried out to determine if phenanthroline‐derived ligand **10** formed thermodynamically more stable complexes with the lanthanides than bipyridine‐derived ligand **8**, as implied by the higher distribution ratios observed in the extraction experiments for **10**. The aliphatic region of the ^1^H NMR spectrum of a 1:1:1 mixture of **8**, **10** and La(NO_3_)_3_ in deuterated acetonitrile is presented in Figure 5. The spectrum displays resonances for the 1:2 bis‐complex of **8**, the 1:2 bis‐complex of **10**, and an additional set of resonances (four methyl resonances, two methylene resonances) which were assigned to the heteroleptic 1:2 bis‐complex [La(**8**)(**10**)(NO_3_)]^2+^. The heteroleptic complex showed one singlet, one triplet, two doublets and a multiplet in the aromatic region (see Supporting Information section 5.4). These resonances were not previously observed in the ^1^H NMR titration of **10** with La(NO_3_)_3_. The ratio of bis‐**8** complex/bis‐**10** complex/heteroleptic bis‐complex was 1:1:2, indicating that a statistical mixture of the three 1:2 bis‐complexes had been formed, in agreement with previous work on ligands **2** and **3**.^19^

NMR experiments were then carried out to determine if each of the ligands **8** and **10** was able to displace the other from its lanthanide 1:2 bis‐complexes. Addition of a solution of ligand **10** (1 equivalent) to a solution of the 1:2 bis‐complex of ligand **8** with La(NO_3_)_3_ (prepared by addition of 1 equivalent of **8** to 0.5 equivalent of La^III^) gave rise to a mixture of the 1:2 bis‐complex of **10**, the heteroleptic 1:2 bis‐complex and free uncomplexed **8** (see Supporting Information section 5.4). The major species present was the 1:2 bis‐complex of **10**, and no traces of the 1:2 bis‐complex of **8** were observed. Thus ligand **10** displaces ligand **8** from its La^III^ complex and forms the thermodynamically more stable complex with La^III^ than **8**.

When a solution of ligand **8** (1 equivalent) was added to a solution of the 1:2 bis‐complex of ligand **10** with La(NO_3_)_3_ (prepared by adding 1 equivalent of **10** to 0.5 equivalents of La^III^), a mixture almost identical in composition to that observed above was obtained (see Supporting Information section 5.4). The 1:2 bis‐complex of **10** was again the major species formed, and no traces of either the 1:2 bis‐complex of **8**, or free uncomplexed **10** were observed. Thus ligand **8** is at best able to displace one of ligand **10** from its 1:2 bis‐complexes but is never able to displace both. We have previously observed the same phenomenon with ligands **2** and **3**.^19^ These results suggest that the order of thermodynamic stability of the three 1:2 bis‐complexes is:

$$\left[\mathrm{La}\left(10\right){}_{2}\left(\mathrm{NO}{}_{3}\right)\right]{}^{2+}>\left[\mathrm{La}\left(10\right)\left(8\right)\left(\mathrm{NO}{}_{3}\right)\right]{}^{2+}>\left[\mathrm{La}\left(8\right){}_{2}\left(\mathrm{NO}{}_{3}\right)\right]{}^{2+}$$

Similarly, a 1:1:1 mixture of ligands **8**, **10** and Y(NO_3_)_3_ in deuterated acetonitrile led again to the expected statistical mixture of the three 1:2 bis complexes (1:2 bis‐complex of **8**, 1:2 bis‐complex of **10**, heteroleptic 1:2 bis‐complex) in a ratio of 1:1:2 (see Supporting Information section 5.4). However, in contrast to La^III^, only partial ligand displacement reactions were observed when either **8** or **10** was added to a solution of the Y^III^ bis‐complex of the other ligand. Addition of **10** to the 1:2 bis‐complex of **8** with Y^III^ led to a mixture containing mostly the bis‐complex of **8** and free uncomplexed ligand **10**, as well as traces of the heteroleptic 1:2 bis‐complex [Y(**8**)(**10**)(NO_3_)]^2+^. Addition of **8** to the 1:2 bis‐complex of **10** with Y^III^ led to a mixture of primarily the bis‐complex of **10** and uncomplexed **8**, as well as traces of the heteroleptic 1:2 bis‐complex. The partial ligand displacement reactions observed here for Y^III^ are likely due to its higher kinetic inertness towards ligand substitution compared to La^III^, in agreement with the lower ligand exchange rate constant observed for the Y^III^ aqua complex.^22^

**Figure 6:**
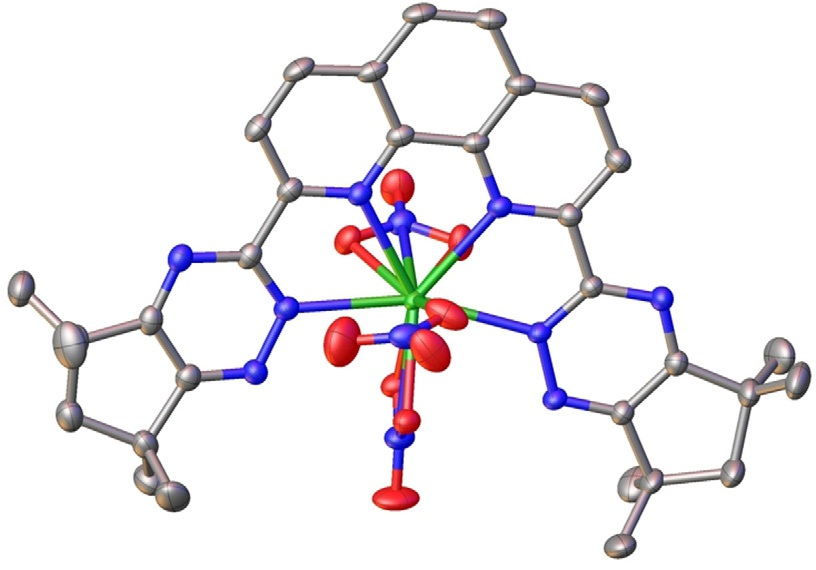
X‐ray crystal structure of Lu(**10**)(NO_3_)_3_ with thermal ellipsoids shown at 50 % probability. Hydrogen atoms and solvent molecules are omitted for clarity. CCDC https://www.ccdc.cam.ac.uk/services/structures?id=doi:10.1002/chem.201903685 contains the supplementary crystallographic data for this structure. These data are provided free of charge by http://www.ccdc.cam.ac.uk/.

To further characterise the various species produced upon complexation, single crystal X‐ray crystallography experiments were performed. Perhaps surprisingly, the crystals grown from solutions of **10** with Y(NO_3_)_3_ or Lu(NO_3_)_3_ were of the minor 1:1 neutral complexes [Y(**10**)(NO_3_)_3_] and [Lu(**10**)(NO_3_)_3_]. The structure of the Lu^III^ complex is shown in Figure 6.

Both structures crystallised as acetonitrile disolvates and were essentially isomorphous, sharing similar unit cell parameters, space groups and packing. The two structures are distinguishable, however, by the coordination of the three nitrate ligands about the lanthanide ion. In the structure of [Y(**10**)(NO_3_)_3_] (see Supporting Information section 3.2), all three nitrate ligands are bidentate with Ln−O distances in the 2.47–2.56 Å range for those in axial positions and around 2.37 Å for the nitrate ligand in the equatorial position which lies in roughly the same plane as the ligand. The structure is very similar to that of the 10‐coordinate [Y(**3**)(NO_3_)_3_] complex published previously.^19^ In contrast, only two of the nitrate ligands in [Lu(**10**)(NO_3_)_3_] are bidentate, one in an axial and the other in an equatorial position (Figure 6). These ligands exhibit significantly shorter Ln−O bond lengths compared to those of [Y(**10**)(NO_3_)_3_] lying in the ca. 2.41–2.44 Å range for the axial positions and around 2.33 Å for the equatorial position. The third nitrate ligand is monodentate with a bond distance of 2.288(2) Å, giving a nine‐coordinate complex overall. This phenomenon has been observed previously in complexes of Lu^III^ with tetradentate bis‐1,2,4‐triazine ligands, where a water molecule displaced one of the nitrate ligands to the outer coordination sphere.^23^

This discrepancy in coordination geometry can be rationalised by considering the ionic radii of the lanthanides in question. With an ionic radius of 86 pm, Lu^III^ is slightly smaller than Y^III^, which has a radius of 90 pm, but this subtle difference may be all that prevents the coordination sphere of Lu^III^ from accommodating a third bidentate nitrate ligand. Further evidence of this constraint on the coordination geometry is observed in the twisting of the triazine rings of **10**, which is more pronounced in the structure of [Lu(**10**)(NO_3_)_3_]. A quantitative measure of this is the N3‐Ln1‐N6 bond angle, which demonstrates the effect of this twist on the coordination of the ligand about Ln1. For [Y(**10**)(NO_3_)_3_] this angle is 165.91(5)° whereas the more pronounced twist observed in [Lu(**10**)(NO_3_)_3_] gives rise to an angle of 161.02(7)°. This very slight deviation is enough to reduce the space available to the monodentate nitrate and prevent it binding in a bidentate fashion while also providing greater access to the metal ion to the nitrate *trans* to it allowing it to bind more strongly and with shorter contacts than the axial nitrate ligands in [Y(**10**)(NO_3_)_3_].

**Figure 7:**
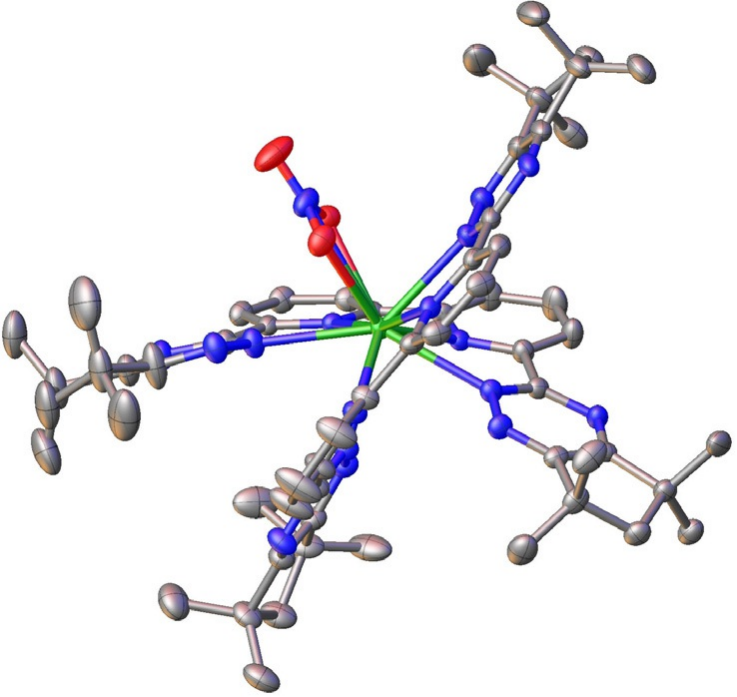
The structure of the Λ independent cation of [Pr(**8**)_2_(NO_3_)][Pr(NO_3_)_5_] with thermal ellipsoids shown at 50 % probability. The structure is disordered; only the components with the largest occupancies are shown. Hydrogen atoms, counterions and solvent molecules are omitted for clarity. CCDC https://www.ccdc.cam.ac.uk/services/structures?id=doi:10.1002/chem.201903685 contains the supplementary crystallographic data for this structure. These data are provided free of charge by http://www.ccdc.cam.ac.uk/.

Attempts to obtain the structures of any of the 1:2 bis‐complex species observed in the course of the NMR titrations proved unsuccessful. However, good quality single crystals of a Pr^III^ complex with ligand **8**, [Pr(**8**)_2_(NO_3_)][Pr(NO_3_)_5_], were grown providing a representative structure of one of these 1:2 bis‐complexes (Figure 7). The asymmetric unit of the structure comprises two crystallographically independent molecules (*Z*′=2), one of each of the Δ and Λ optical isomers. In terms of their coordination, the structure is very similar to those of **2** with Eu^III[24]^ and **3** with Pu^III^.^25^ As this is the case it is probably safe to assume that the coordination of the ligands about the lanthanide is similar across the series and that any effect of the lanthanide contraction will be manifest in the coordination of the nitrate as was observed in the structures of the 1:1 species.

The X‐ray crystal structure of free ligand **8** (see Supporting Information section 3.2) shows that the ligand adopts the non‐chelating *trans* conformation in the solid state with respect to the C−C torsion between the central pyridine rings. This was also observed in the structure of the analogous ligand **2**,^23^ and is due to the high torsional barrier to rotation about this C−C bond when the ligand adopts the chelating *cis* conformer.^19^

### TRLFS measurements and DFT calculations

To gain further insight on the speciation in solution and support the NMR and X‐ray crystallography findings, the complexation of Cm^III^ and Eu^III^ with ligand **10** was studied by time‐resolved laser fluorescence spectroscopy. This technique allows the study of the coordination chemistry of fluorescent metal ions.^26^, ^27^ Cm^III^ and Eu^III^ represent trivalent actinides and lanthanides, respectively with excellent fluorescence properties.

#### Complexation kinetics

**Figure 8:**
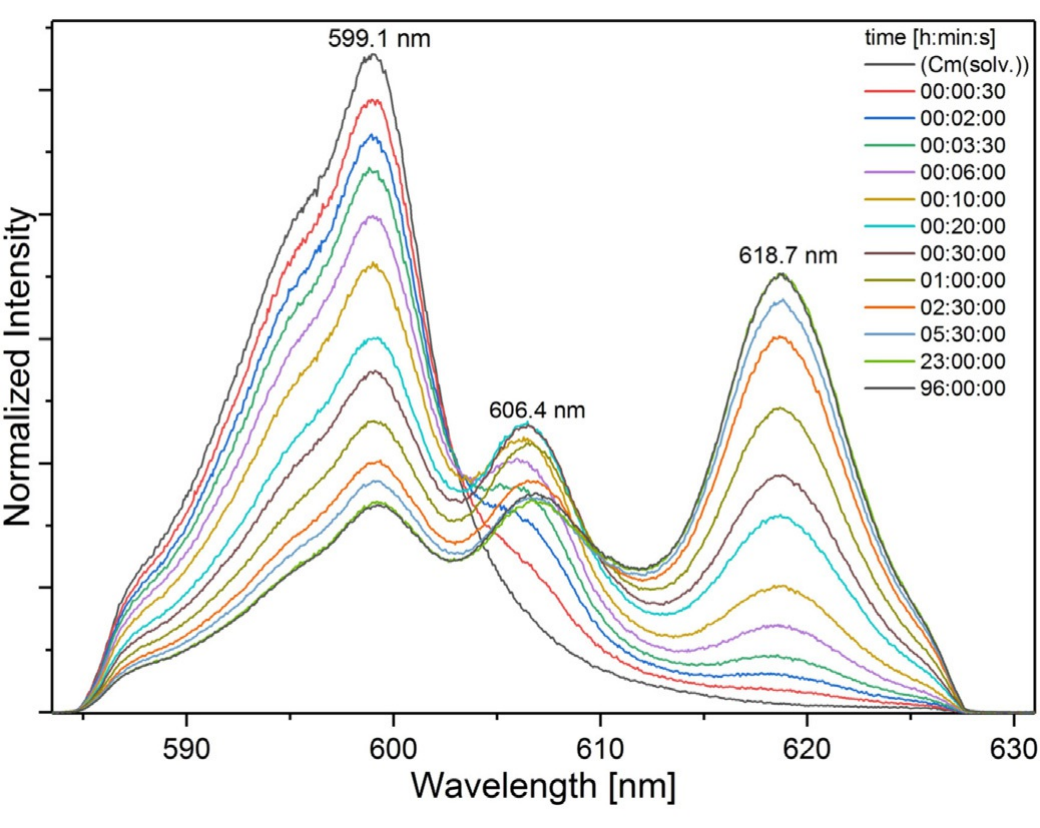
Normalized Cm^III^ fluorescence spectra as a function of time after addition of ligand **10** in MeOH+1.5 vol. % H_2_O (*c*(H^+^)=91.2 mm. *c*(**10**)=9.90×10^−8^ m; *c*(Cm^III^)=4.69×10^−8^ m).

Tetradentate bis‐1,2,4‐triazine derivatives such as **3** show relatively slow complexation kinetics.^28^ Therefore, the fluorescence emission of Cm^III^ at a given ligand concentration was measured as a function of time after addition of **10**. Cm^III^ fluorescence spectra resulting from the ^6^D_7/2_→^8^S_7/2_ transition are shown in Figure 8. Without addition of **10** the Cm^III^ solvent spectrum at 599.1 nm was observed with a shoulder at 595.4 nm. Upon addition of **10** the emission band at 599.1 nm decreased and new emission bands at 606.4 nm and 618.7 nm occurred. With time the emission band at 618.7 nm became dominant. No further changes of the Cm^III^ fluorescence spectrum were observed after 23 h, indicating that the system was at equilibrium.

In the case of Eu^III^, the fluorescence emission bands of the ^5^D_0_→^7^F_*n*_ (*n*=0, 1,2) transitions were studied as a function of time after addition of **10** (see Supporting Information section 6.1). Without **10** an emission band at 578.9 nm for the ^5^D_0_→^7^F_0_ transition of the Eu^III^ solvent species was observed. Upon addition of **10** two new emission bands at 579.5 nm and 581.1 nm occurred. The ^5^D_0_→^7^F_1_ and ^5^D_0_→^7^F_2_ transitions exhibited a change of shape and splitting of the emission bands due to complexation of Eu^III^ with **10**. No further changes of the Eu^III^ fluorescence emission spectra were observed after 4.5 h, confirming chemical equilibrium.

#### Complexation of Cm^III^ and Eu^III^ with ligand 10

**Figure 9:**
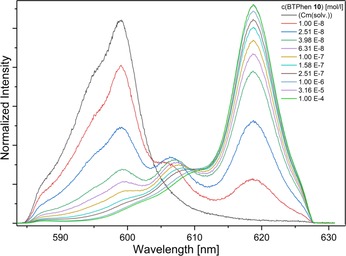
Normalized Cm^III^ fluorescence spectra as a function of the concentration of ligand **10** in MeOH+1.5 vol. % H_2_O (*c*(H^+^)=91.2 mm; *c*(Cm^III^)_ini_=4.69×10^−8^ m).

To determine thermodynamic data for the complexation of Cm^III^ and Eu^III^ with **10**, the evolution of the fluorescence spectra of Cm^III^ and Eu^III^ as a function of the concentration of **10** was studied in nitrate free media. Batch samples containing increasing concentrations of **10** were equilibrated for 24 h before being measured. The normalized Cm^III^ fluorescence spectra are shown in Figure 9. The formation of two species at 606.4 nm and 618.7 nm was observed. Single component spectra for the Cm^III^ solvent species and both complex species are shown in the Supporting Information (section 6.2).

**Figure 10:**
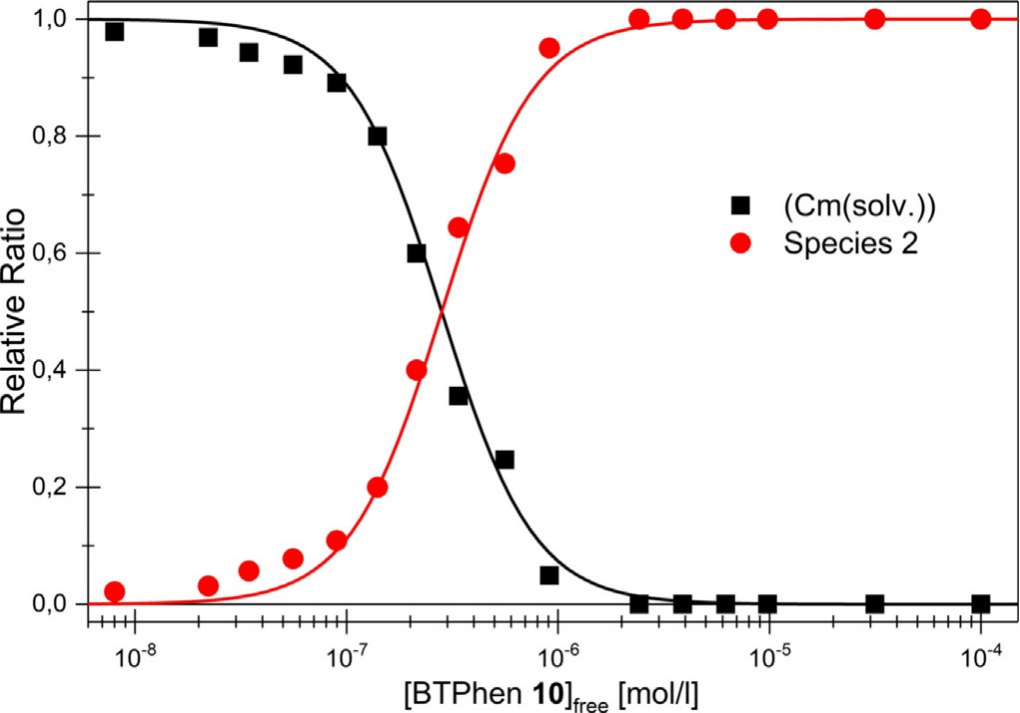
Relative ratio of Cm(solv.) and species 2 as a function of the concentration of ligand **10**. Symbols represent experimental data whereas lines denote calculations.

The fluorescence intensity factor (FI) describes the fluorescence intensity of a species relative to a reference. Due to the high FI of species 2 (FI=56±6), the speciation was determined from the overall fluorescence intensity. The speciation is shown in Figure 10. The formation of species 2 starts at 8×10^−9^ m of free **10** and becomes dominant at 2.8×10^−7^ m. The relative ratio of species **1** is irrelevant (<3 %) under the applied conditions and is therefore not shown in Figure 10. Slope analysis according to Equation (1) was performed to determine the stoichiometry of species 2.

(1) $$M^{3+}+n\;L\overset{⇀}{↽}{\left({\mathrm{ML}}_{n}\right)}^{3+}\log\frac{c\left({\left({\mathrm{ML}}_{n}\right)}^{3+}\right)}{c\left(M^{3+}\right)}=n\log c\left(L\right)+\log K{}^{'}$$

(2) $$\beta {{}^{'}}_{2}=\frac{c\left({\left({\mathrm{ML}}_{2}\right)}^{3+}\right)}{c\left(M^{3+}\right){\left(c\left(L\right)\right)}^{2}}$$

The slope of the linear correlation indicates the number of coordinated ligand molecules. A slope of 2.11±0.17 was obtained, showing that species 2 is the 1:2 complex [Cm(**10**)_2_]^3+^ (see Supporting Information section 6.2). The conditional stability constant for the formation of the 1:2 complex according to Equation (2) is log *β*′_2_=13.1±0.2.

Fluorescence spectra of the Eu^III 5^D_0_→^7^F_0_ transition are shown in the Supporting Information (section 6.2). Since neither the excited state (^5^D_0_) nor the ground state (^7^F_0_) are split (*J*=0), the number of emission bands accounts for the number of species present in the system.

The Eu^III^ solvent spectrum was observed at 578.9 nm. Upon addition of **10** two new emission bands at 579.5 and 581.1 nm occurred, indicating the formation of two different species. Eu^III^ speciation (see Supporting Information section 6.2) was determined from the overall fluorescence intensity due to the high FI factor of species 2 (FI_2_=1325±130). Again, species 1 is only present at irrelevant concentrations and is not shown in the speciation. Slope analysis resulted in a slope of 2.05±0.06, confirming the formation of the 1:2 complex [Eu(**10**)_2_]^3+^. The conditional stability constant for this complex is log *β*′_2_=10.3±0.4.

Comparing both tetradentate phenanthroline‐derived ligands **10** and **3** under the same conditions, it is evident that **3** is a stronger ligand than **10**. The stability constants for both the Cm^III^ and the Eu^III^ 1:2 complexes are approximately one order of magnitude lower in the case of **10** (Table 2).

**Table 2:** Comparison of FI factors and stability constants for the complexation of Cm^III^ and Eu^III^ with tetradentate ligands **10** and **3** in MeOH with 1.5 vol % H_2_O (*c*(H^+^)=91.2 mm).

	Ligand **10**	Ligand **3** ^[a]^
FI_2_ factor	Cm^III^: 56±6 Eu^III^: 1325±130	Cm^III^: 82±8 Eu^III^: 1414±140
Log *β*′_2_	Cm^III^: 13.1±0.2 Eu^III^: 10.3±0.4	Cm^III^: 13.8±0.2 Eu^III^: 11.6±0.4

[a] Ref. 28.

#### Comparison of mono‐ and biphasic experiments

Tetradentate bis‐1,2,4‐triazine ligands extract trivalent actinide and lanthanide ions from nitric acid or nitrate solutions as 1:2 complexes.10b, 17, 29 With **2** and **3**, the extracted complexes were previously shown to be [ML_2_(NO_3_)]^2+^ complexes containing one inner‐sphere nitrate anion (L=**2** or **3**).^28^ The possible presence of an inner sphere nitrate in the 1:2 complexes with **10** was studied in a similar manner by extracting Cm^III^ or Eu^III^ from solutions containing 0.1 m nitric acid and 1.9 m NH_4_NO_3_ into solutions of 10 mm **10** in 1‐octanol. After phase separation, the organic phases were studied by time‐resolved laser fluorescence spectroscopy.

**Figure 11:**
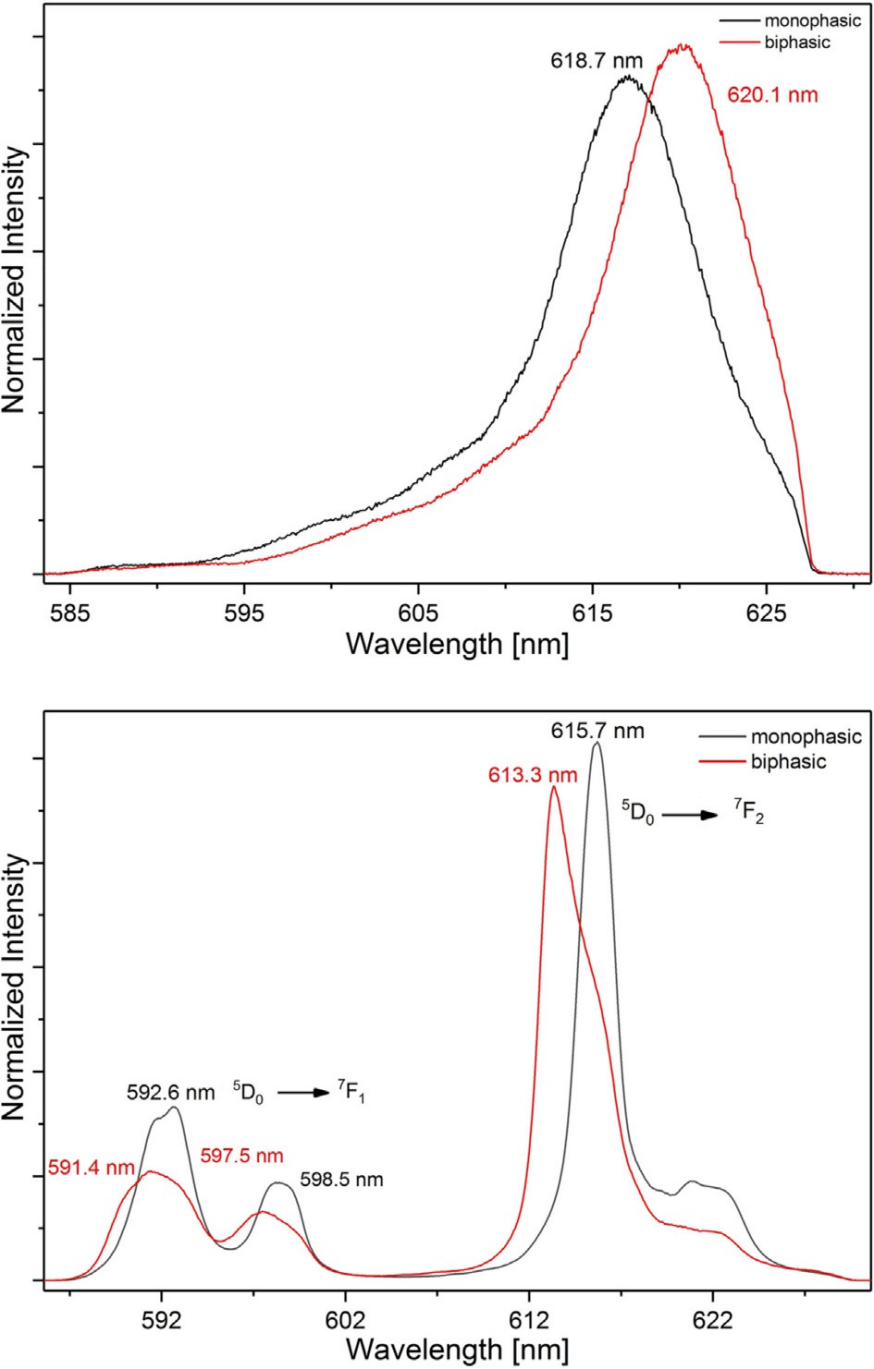
Comparison of the (black) fluorescence emission spectra of the [M(**10**)_2_]^3+^ complexes in a monophasic batch experiment and (red) the formed complexes in the organic phase after extraction (Top: M=Cm^III^; bottom: M=Eu^III^; ^5^D_0_→^7^F_*n*_ transitions (*n*=1, 2)).

Figure 11 compares the Cm^III^ (top) and Eu^III^ (bottom) spectra of the 1:2 complexes of **10** in methanol with those from the solvent extraction experiments. The emission spectrum of the extracted Cm^III^ complex shows an emission band at 620.1 nm, which is bathochromically shifted by 1.4 nm with respect to the emission band of the [Cm(**10**)_2_]^3+^ complex (618.7 nm). In the case of Eu^III^, the emission band of the ^5^D_0_→^7^F_1_ and ^5^D_0_→^7^F_2_ transitions of the [Eu(**10**)_2_]^3+^complex and the complex in the organic phase of the extraction experiment differ in shape and position. The emission band (^5^D_0_→^7^F_2_ transition) of the complex formed during the extraction experiment displays a peak maximum at 613.3 nm while the emission band of the [Eu(**10**)_2_]^3+^ complex exhibits a peak maximum at 615.7 nm. Similar shifts and changes in position and shape of the emission bands were observed for **2** and **3** and were assigned to the additional complexation of a nitrate ion in the inner coordination sphere of Cm^III^.^28^

Adding ammonium nitrate to a solution containing the [Cm(**10**)_2_]^3+^ complex resulted in a comparable bathochromic shift of the emission band from initially 618.7 nm (no nitrate added) to 619.2 nm (9.97×10^−2^ m of nitrate added), as shown in the Supporting Information (section 6.3).

In the case of Eu^III^, a change in shape of the emission band resulting from the ^5^D_0_→^7^F_2_ transition was observed (see Supporting Information section 6.3). With increasing nitrate concentration, a new peak at 613.3 nm appeared, indicating the formation of the same species observed in the extraction experiments. Thus ligand **10** extracts Cm^III^ and Eu^III^ from acidic nitrate solutions as [M(**10**)_2_(NO_3_)]^2+^ complexes, as was previously observed for **2** and **3**.^28^

In an attempt to gain further insight into why An^III^ complexes of ligand **10** are less stable than those of ligand **3**, DFT calculations were carried out on free ligands **3** and **10** and their respective [AmL_2_(NO_3_)]^2+^ complexes (L=**3** or **10**) using a level of theory successfully used in previous studies of actinide complexes.^30^ Comparison of the energies of the complexes relative to the respective free ligand conformations of lowest energy enabled the relative complexation energies of the ligands to be determined. The results indicate that the binding energy of **3** when forming [Am(**3**)_2_(NO_3_)]^2+^ was 1.76 kJ mol^−1^ less favorable than the binding of **10** when forming [Am(**10**)_2_(NO_3_)]^2+^ in an acetonitrile solvent field (see Supporting Information section 7). Similar values were obtained from calculations in the gas phase and in a 1‐octanol solvent field. This suggests that the 1:2 complex of **10** is marginally more stable than that of **3**, but that neither has a significantly greater binding energy than the other. This may indicate that there is little difference between the inherent metal binding energies of the ligands **3** and **10**, and that the differences in extraction properties observed above arise instead from specific solvent interactions.

## Conclusion

We report on three novel bis‐1,2,4‐triazine ligands derived from a five‐membered ring diketone, and we show for the first time how tuning the aliphatic ring size of bis‐1,2,4‐triazine ligands leads to subtle changes in the speciation of the ligands with trivalent *f*‐block metal ions, the thermodynamic stabilities of the formed metal complexes, and the trivalent actinide extraction affinities of the ligands. We propose that this insight could enable a more rational design of actinide‐selective ligands with tailored solvent extraction properties suitable for future spent nuclear fuel reprocessing to close the nuclear fuel cycle.

## Conflict of interest

The authors declare no conflict of interest.

## Supporting information

As a service to our authors and readers, this journal provides supporting information supplied by the authors. Such materials are peer reviewed and may be re‐organized for online delivery, but are not copy‐edited or typeset. Technical support issues arising from supporting information (other than missing files) should be addressed to the authors.

Supplementary
