# Supplementary material for: Exploring the Subtle Effect of Aliphatic Ring Size on Minor Actinide‐Extraction Properties and Metal Ion Speciation in Bis‐1,2,4‐Triazine Ligands
Source: Chemistry. 2019 Oct 30;26(2):428–37. doi: 10.1002/chem.201903685 (PMC7027750; doi:10.1002/chem.201903685)
Supplement: Supplementary file 1 — Supplementary [file CHEM-26-428-s001.pdf]

CHEMISTRY
A European Journal

Supporting Information

Exploring the Subtle Effect of Aliphatic Ring Size on Minor Actinide-Extraction Properties and Metal Ion Speciation in Bis-1,2,4-Triazine Ligands

Andrey V. Zaytsev,[a] Rachel Bulmer,[a] Valery N. Kozhevnikov,[a] Mark Sims,[a] Giuseppe Modolo,[b] Andreas Wilden,[b] Paul G. Waddell,[c] Andreas Geist,[d] Petra J. Panak,[d, e] Patrik Wessling,[d, e] and Frank W. Lewis*[a]

chem_201903685_sm_miscellaneous_information.pdf

ELECTRONIC SUPPLEMENTARY INFORMATION

for the paper entitled

Exploring the Subtle Effect of Aliphatic Ring Size on Minor Actinide Extraction Properties and Metal Ion Speciation in Bis-1,2,4-Triazine Ligands

Andrey V. Zaytsev,[a] Rachel Bulmer,[a] Valery N. Kozhevnikov,[a] Mark Sims,[a] Giuseppe Modolo,[b] Andreas Wilden,[b] Paul G. Waddell,[c] Andreas Geist,[d] Petra J. Panak,[d,e] Patrik Wessling[d,e] and Frank W. Lewis *[a]

[a] *Department of Applied Sciences, Faculty of Health and Life Sciences, Northumbria University, Newcastle upon Tyne NE1 8ST, UK. E-mail: frank.lewis@northumbria.ac.uk*

[b] *Forschungszentrum Jülich GmbH, Institut für Energie und Klimaforschung - Nukleare Entsorgung und Reaktorsicherheit (IEK-6), 52428 Jülich, Germany. E-mail: g.modolo@fz-juelich.de*

[c] *School of Natural and Environmental Sciences, Newcastle University, Kings Road, Newcastle upon Tyne NE1 7RU, UK. E-mail: paul.waddell@newcastle.ac.uk*

[d] *Karlsruhe Institute of Technology (KIT), Institute for Nuclear Waste Disposal (INE), 76021 Karlsruhe, Germany. E-mail: andreas.geist@kit.edu*

[e] *Ruprecht-Karls-Universität Heidelberg, Physikalisch-Chemisches Institut, Im Neuenheimer Feld, 69120 Heidelberg, Germany. E-mail: petra.panak@kit.edu*

CONTENTS **PAGE**

1.		**Experimental Procedures**	3
2.		**NMR Spectra**	9
3.		**X-ray Crystallography**	19
	3.1	Crystal Data and Structure Refinement	19
	3.2	Crystal Structures	23
4.		**Solvent Extraction Studies**	27
	4.1	Extraction Studies for Ligand **6**	27
	4.2	Extraction Studies for Ligand **8**	32
	4.3	Extraction Studies for Ligand **10**	42
5.		**NMR Titrations with Metal Salts**	48
	5.1	NMR Titrations of Ligand **6** with Metal Salts	48
	5.2	NMR Titrations of Ligand **8** with Metal Salts	51
	5.3	NMR Titrations of Ligand **10** with Metal Salts	52
	5.4	NMR Competition Experiments	58
6.		**TRLFS Measurements**	68
	6.1	Complexation Kinetics with Ligand **10**	68
	6.2	Complexation of Cm(III) and Eu(III) with Ligand **10**	69
	6.3	Comparison of Mono- and Biphasic Experiments	77
7.		**DFT Calculations**	80
8.		**References**	84

1: Experimental Procedures

General procedures

All solvents and reagents were purchased from Sigma-Aldrich, Acros Organics, Fluorochem or Alfa-Aesar and used without further purification unless otherwise specified. Reactions were monitored by TLC using silica gel with UV_{254} fluorescent indicator. Uncorrected melting points were measured in open capillary tubes using a DigiMelt MPA161 SRS instrument. NMR spectra were recorded on a JEOL ECS400FT Delta spectrometer (399.78 MHz for ^{1}H NMR, 100.53 MHz for ^{13}C NMR). Chemical shifts are reported in parts per million (ppm) relative to tetramethylsilane as internal standard. Coupling constants (*J*) are measured in hertz. Multiplets are reported as follows: b = broad, s = singlet, d = doublet, dd = double doublet, t = triplet, q = quartet, m = multiplet, app d = apparent doublet, app t = apparent triplet. Low resolution mass spectra were obtained in methanol solutions on a Thermo Finnigan LCQ Advantage MS detector using electrospray ionisation (ESI). High resolution mass spectra were obtained on a Finnigan MAT900XLT high-resolution double focussing MS spectrometer using nano-electrospray ionisation (NESI) at the EPSRC National Mass Spectrometry Service (University of Swansea). Column chromatography was conducted using 0.060–0.20 mm silica gel (70–230 mesh), and automated flash column chromatography was performed using a Biotage Isolera One ISO-1SV instrument. Bis-amidrazones **4**,[1,2] **7**[1,2] and **9**[3] were synthesized as previously described in the literature. 2,2,4,4-Tetramethylcyclopentanone was obtained as a generous gift from Merck.

3,3,5,5-Tetramethylcyclopentan-1,2-dione 5[4]

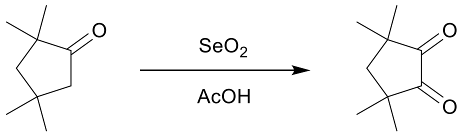

5

2,2,4,4-Tetramethylcyclopentanone (5.45 g, 39.4 mmol) was dissolved in acetic acid (75 mL) and selenium(IV) oxide (3.93 g, 35.46 mmol, 0.9 eq) was added. The flask was heated under reflux for 1.5 hours. The flask was allowed to cool to room temperature and the precipitated selenium metal was filtered. The filtrate was evaporated to a small volume and diethyl ether (200 mL) was added. The solution was washed successively with water (4 × 30 mL), saturated sodium hydrogen carbonate (30 mL) and brine (30 mL) and then dried over

magnesium sulfate, filtered and evaporated to afford the title compound **5** as a bright pink solid (5.40 g, 89%). δ_H(399.8 MHz, $CDCl_3$, Me_4Si) 1.23 (12H, s, 4 × C*H*$_3$), 1.98 (2H, s, C*H*$_2$).

2,6-Bis(5,5,7,7-tetramethyl-6,7-dihydro-5*H*-cyclopenta[*e*]-1,2,4-triazin-3-yl)pyridine 6

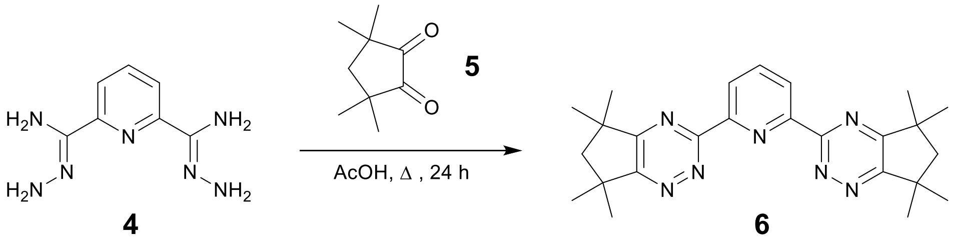

Pyridine-2,6-dicarbohydrazonamide **4**[1,2] (0.31 g, 1.604 mmol) was suspended in acetic acid (7 mL) in a round-bottomed flask and 3,3,5,5-tetramethylcyclopentan-1,2-dione **5** (0.544 g, 3.53 mmol, 2.1 eq) was added. The flask was heated under reflux for 24 hours. The flask was allowed to cool to room temperature and the solvent was evaporated to a small volume. The residue was dissolved in dichloromethane (50 mL) and the solution was dried over magnesium sulfate, filtered and evaporated. The solid was triturated with petroleum ether (20 mL) and then filtered and washed with petroleum ether (20 mL) to afford the title compound **6** as a light-yellow solid (0.517 g, 75%). Mp 208.2–208.8 ^oC (from dichloromethane). δ_H(399.8 MHz, $CDCl_3$, Me_4Si) 1.49 (12H, s, 4 × C*H*$_3$), 1.51 (12H, s, 4 × C*H*$_3$), 2.05 (4H, s, 2 × C*H*$_2$), 8.09 (1H, t, *J* 7.6, 4-H), 8.73 (2H, d, *J* 7.6, 3-H and 5-H). δ_C(100.5 MHz, $CDCl_3$, Me_4Si) 29.1 (4 × *C*H$_3$), 29.8 (4 × *C*H$_3$), 40.4 (2 × quat), 41.5 (2 × quat), 53.1 (2 × *C*H$_2$), 125.7 (C-3 and C-5), 138.2 (C-4), 154.0 (2 × quat), 162.5 (2 × quat), 168.0 (2 × quat), 171.3 (2 × quat). *m/z* (HRMS, ESI) 430.2709 ($[M + H]^+$); $C_{25}H_{32}N_7$ requires 430.2714.

6,6’-Bis(5,5,7,7-tetramethyl-6,7-dihydro-5*H*-cyclopenta[*e*]-1,2,4-triazin-3-yl)-2,2’-bipyridine 8

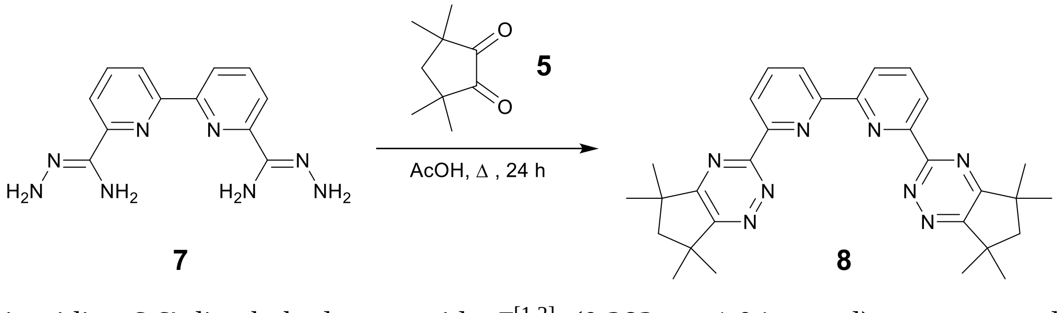

2,2'-Bipyridine-6,6'-dicarbohydrazonamide **7**[1,2] (0.282 g, 1.04 mmol) was suspended in acetic acid (5 mL) in a round-bottomed flask and 3,3,5,5-tetramethylcyclopentan-1,2-dione **5** (0.322 g, 2.09 mmol, 2.0 eq) was added. The flask was heated under reflux for 24 hours. The

flask was allowed to cool to room temperature and the solvent was evaporated to a small volume. The residue was dissolved in dichloromethane (50 mL) and the solution was dried over magnesium sulfate, filtered and evaporated. The solid was dissolved in dichloromethane (2 mL) and diluted with petroleum ether (10 mL). The precipitated solid was filtered and washed with petroleum ether (10 mL) to afford the title compound **8** as a yellow solid (0.311 g, 59%). Mp 251.6–252.2 °C (from dichloromethane). δ_H(399.8 MHz, $CDCl_3$, Me_4Si) 1.48 (12H, s, 4 × C*H*$_3$), 1.53 (12H, s, 4 × C*H*$_3$), 2.07 (4H, s, 2 × C*H*$_2$), 8.05 (2H, t, *J* 7.6, 4-H and 4'-H), 8.54 (2H, d, *J* 7.6, 5-H and 5'-H), 8.91 (2H, d, *J* 7.6, 3-H and 3'-H). δ_C(100.5 MHz, $CDCl_3$, Me_4Si) 29.1 (4 × *C*H$_3$), 29.8 (4 × *C*H$_3$), 40.4 (2 × quat), 41.3 (2 × quat), 53.0 (2 × *C*H$_2$), 123.0 (C-3 and C-3'), 124.4 (C-5 and C-5'), 138.0 (C-4 and C-4'), 153.1 (2 × quat), 156.2 (2 × quat), 162.9 (2 × quat), 167.6 (2 × quat), 170.9 (2 × quat). *m*/*z* (HRMS, ESI) 507.2972 ($[M + H]^+$); $C_{30}H_{35}N_8$ requires 507.2979.

2,9-Bis(5,5,7,7-tetramethyl-6,7-dihydro-5*H*-cyclopenta[*e*]-1,2,4-triazin-3-yl)-1,10-phenanthroline 10

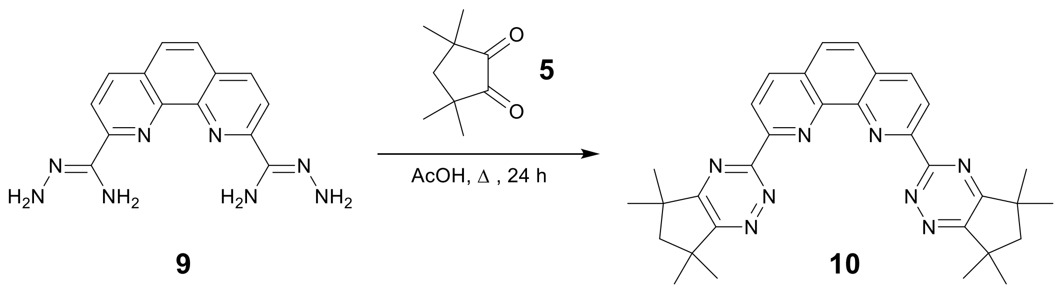

1,10-Phenanthroline-2,9-dicarbohydrazonamide **9**[3] (0.313 g, 1.063 mmol) was suspended in acetic acid (5 mL) in a round-bottomed flask and 3,3,5,5-tetramethylcyclopentan-1,2-dione **5** (0.361 g, 2.34 mmol, 2.2 eq) was added. The flask was heated under reflux for 24 hours. The flask was allowed to cool to room temperature and the solvent was evaporated to a small volume. The residue was dissolved in dichloromethane (50 mL) and the solution was dried over magnesium sulfate, filtered and evaporated. The solid was dissolved in dichloromethane (2 mL) and diluted with petroleum ether (10 mL). The precipitated solid was filtered and washed with petroleum ether (10 mL) to afford the title compound **10** as a dark yellow solid (0.474 g, 84%). Mp >260 °C (from dichloromethane). δ_H(399.8 MHz, $CDCl_3$, Me_4Si) 1.54 (12H, s, 4 × C*H*$_3$), 1.58 (12H, s, 4 × C*H*$_3$), 2.07 (4H, s, 2 × C*H*$_2$), 7.94 (2H, s, 5-H and 6-H), 8.46 (2H, d, *J* 8.4, 4-H and 7-H), 8.94 (2H, d, *J* 8.4, 3-H and 8-H). δ_C(100.5 MHz, $CDCl_3$, Me_4Si) 29.2 (4 × *C*H$_3$), 29.8 (4 × *C*H$_3$), 40.5 (2 × quat), 41.6 (2 × quat), 53.2 (2 × *C*H$_2$), 123.7 (C-3 and C-8), 127.7 (C-5 and C-6), 129.8 (2 × quat), 137.4 (C-4 and C-7), 146.7 (2 × quat),

153.9 (2 × quat), 163.3 (2 × quat), 167.8 (2 × quat), 171.5 (2 × quat). *m/z* (HRMS, ESI) 531.2969 ($[M + H]^+$); $C_{32}H_{35}N_8$ requires 531.2979.

Solvent Extraction Measurements

The aqueous solutions were prepared by spiking nitric acid solutions (0.01–3 mol dm^{-3}) containing 1×10^{-5} mol dm^{-3} of each lanthanide (+Y, w/o Pm) with stock solutions of ^{241}Am, ^{152}Eu and ^{244}Cm tracers (10 µL) in nitric acid. Ultrapure water (18.2 MΩcm) was used for all dilutions. The radiotracers ^{241}Am, ^{244}Cm and ^{152}Eu were supplied by Isotopendienst M. Blaseg GmbH, Waldburg (Germany), Oak Ridge National Laboratory, Oak Ridge (USA), and Eckert & Ziegler Nuclitec GmbH, Braunschweig (Germany), respectively. Solutions of the ligands **6**, **8** and **10** (0.01 mol dm^{-3}) were prepared by dissolving **6**, **8** or **10** in 1-octanol, with or without *N*,*N*'-dimethyl-*N*,*N*'-dioctyl-2-hexyloxyethyl malonamide **11** as an additional phase modifier. Each organic phase (500 µL) was shaken separately with each of the aqueous phases (500 µL) for one hour at 22 °C using a thermostatted aluminum block installed on an IKA Vibrax Orbital Shaker Model VXR (2,200 rpm). The contact time of one hour was sufficient to attain the distribution equilibrium. After phase separation by centrifugation, 200 µL aliquots of each phase were withdrawn for radio analysis. Activity measurements of the γ-ray emitters ^{241}Am and ^{152}Eu were performed with a HPGe γ-ray spectrometer, EG & G Ortec, Munich (Germany). The γ-lines at 59.5 keV, and 121.8 keV were examined for ^{241}Am, and ^{152}Eu, respectively. The nuclides ^{241}Am and ^{244}Cm were measured by means of alpha spectrometry with an Alpha Spectrometer OctêteTM PC obtained from EG & G Ortec, Munich (Germany). Stable elements were determined by ICP-MS on a NexION 2000 obtained from Perkin Elmer Sciex, Roggau-Jügesheim (Germany). The concentration of inactive elements in organic phases was measured via ICP-MS using Triton-X 100 as surfactant. The distribution ratio *D* was calculated as the ratio between the radioactivity/concentration in the organic and the aqueous phase. The separation factor SF is calculated as the ratio between the distribution ratios of the corresponding metals. Distribution ratios between 0.01 and 100 exhibit a maximum error of ± 5 %. The error may be up to ± 20 % for smaller and larger values.

NMR Titrations and Competition Experiments

Stock solutions (0.01 mol dm^{-3}) of the ligands **6**, **8** and **10**, and of the metal nitrate salts $La(NO_3)_3.6H_2O$, $Lu(NO_3)_3.H_2O$, $Yb(NO_3)_3.H_2O$ and $Y(NO_3)_3.6H_2O$ (Aldrich) were prepared

in CD_3CN (Fluorochem). A 0.5 mL aliquot of the appropriate ligand solution was placed in an NMR tube and the ^1H NMR spectrum was recorded at 399.8 MHz on a JEOL ECS400FT Delta spectrometer. The appropriate lanthanide salt solution was added to the NMR tube in 50 μL aliquots (ie: 0.1 equivalents each time) using a calibrated Gilson 100 μL micropipette. The tube was inverted several times to ensure full mixing and the ^1H NMR spectrum was recorded after each successive addition until the resonances of the free ligand had completely disappeared and/or until no further spectral changes were observed. Homogeneous solutions were obtained after each addition. The relative ratios of the different species present were calculated from the relative integrals of a suitable one-proton resonance of the ligand **6**, **8** or **10**. These values were normalized such that, for a given one-proton resonance, the total integration for all species present equalled unity. The species distributions at different metal:ligand ratios were calculated from these normalized relative ratios. The same solutions were used for the NMR competition experiments. Solutions of the 1:2 complexes of **8** or **10** were obtained by adding the metal (100 μL, 1 equivalent) to the ligand **8** or **10** (200 μL, 2 equivalents). The ^1H NMR spectrum was then recorded prior to addition of the second ligand (200 μL, 2 equivalents). The ^1H NMR spectra of the 1:1:1 mixtures of **8**, **10** and metal nitrate salt were obtained by prior mixing of solutions of **8** and **10** (200 μL each) in an NMR tube before addition of the metal salt solution (200 μL).

X-ray Crystallography

All crystal structure data were collected using molybdenum radiation ($\lambda_{MoK\alpha}$ = 0.71073 Å) at 150 K on an Xcalibur, Atlas, Gemini diffractometer (Rigaku Oxford Diffraction) equipped with an Oxford Cryosystems CryostreamPlus open-flow N_2 cooling device. For **8** and Lu(**10**)$(NO_3)_3$, intensities were corrected for absorption empirically using spherical harmonics. In the case of Y(**10**)$(NO_3)_3$ intensities were corrected for absorption numerically using a multi-faceted crystal model created by indexing the faces of the crystal for which data were collected.[5] Unit cell measurement, data collection and data reduction were performed using the software CrysAlisPro.[6]

All structures were solved using XT[7] and refined by XL[8] using the Olex2 interface.[9] All non-hydrogen atoms were refined anisotropically and hydrogen atoms were positioned with idealised geometry with their displacement parameters U_H constrained using a riding model to be an appropriate multiple of the U_{eq} value of the parent atom.

TRLFS Measurements

All experiments were conducted in MeOH (Millipore, Uvasol®) with 1.5 vol.% of water and a proton concentration of 91.2 mM. Cm(III) batch samples consisted of 985 µL MeOH, 7.8 µL $HClO_4$ (11.7 M, Millipore, Suprapur®), 5 µL ultrapure water (Millipore, Billerica, USA; 18.2 MΩ·cm) and 2.2 µL Cm(III) stock solution (2.12×10^{-5} M $Cm(ClO_4)_3$ in 0.1 M $HClO_4$; ^{248}Cm: 89.7%, ^{246}Cm: 9.4%, ^{243}Cm: 0.4%, ^{244}Cm: 0.3%, ^{245}Cm: 0.1%, ^{247}Cm: 0.1%), resulting in an initial Cm(III) concentration of 4.69×10^{-8} M. Eu(III) batch experiments consisted of 985 µL MeOH, 7.8 µL $HClO_4$ and 7.2 µL Eu(III) stock solution (1.07 mM $Eu(ClO_4)_3$ in 0.1 M $HClO_4$) resulting in an initial Eu(III) concentration of 7.68×10^{-6} M. The ligand concentrations were adjusted by adding known amounts of a stock solution of **10** to the aforementioned solvent. The stock solutions of **10** were prepared by dissolving 1.13 mg in 2 mL of solvent.

For extraction experiments 500 µL of both organic and aqueous phases were shaken on an orbital vortex shaker at 2500 rpm for 1 h at 293 K. Samples were centrifuged at 6000 rpm for 2 min, phases were separated and 300 µL were investigated by TRLFS. Aqueous phases consisted of 10^{-7} M Cm(III) or 7.68×10^{-6} M Eu(III) in 0.1 M nitric acid containing 1.9 M NH_4NO_3. Organic phases consisted of 10 mM **10** in 1-octanol.

Experiments were carried out at 293 K and atmospheric pressure. For TRLFS a Nd:YAG Laser (Surelite II laser, Continuum) was used that pumps a Dye Laser (Narrow Scan D-R; Radiant Dyes Laser Accessories GmbH) using wavelengths of 396.6 nm for Cm(III) and 394 nm for Eu(III). Spectral decomposition was performed with a spectrograph (Shamrock 303i, ANDOR) with 300, 1199 and 2400 lines per mm grating. The fluorescence emission was detected by an ICCD camera (iStar Gen III, ANDOR) with a delay of 1 µs after excitation and a gate width of 1 ms to discriminate short-lived species.

DFT Calculations

All calculations were carried out using Gaussian09.[10] Geometry optimisations were performed using the B3LYP functional,[11,12] with the def-SVP all-electron basis sets[13] for light atoms, and the same basis set[14] with accompanying 60-electron core pseudo potential (ECP) for Am(III).[15,16] Subsequent energy calculations were carried out using the def-TZVP basis set for light atoms,[17] again using the same basis set for Am(III)[14] with the corresponding ECP.[15,16] This level of theory has been used successfully in previous studies of actinide complexes[18,19] and shown to give comparable results to other DFT methods.[20] Solvation energies were calculated using the SMD solvation model.[21]

2: NMR Spectra

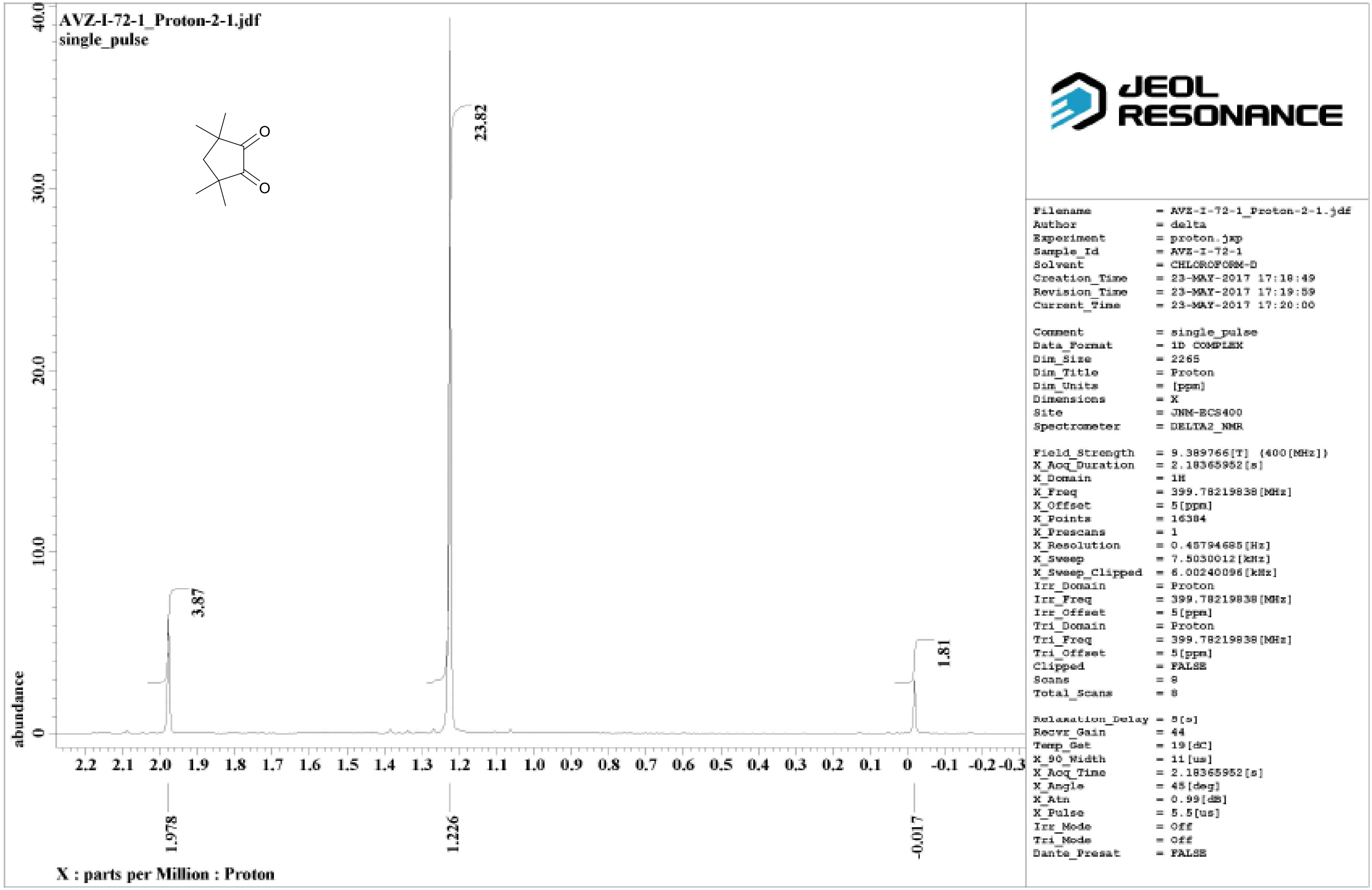

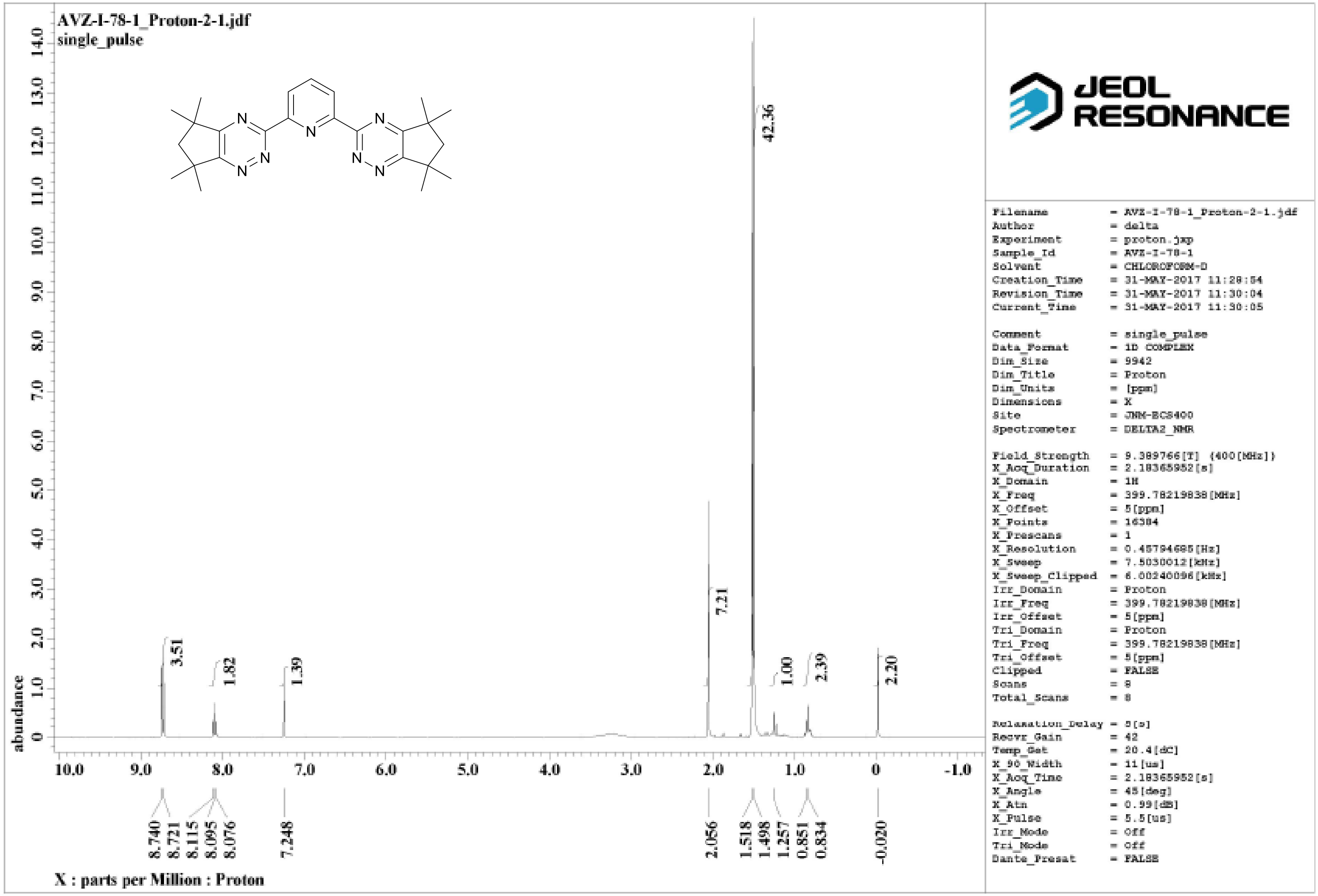
AVZ-I-78-1_Proton-2-1.jdf
single_pulse
abundance
X : parts per Million : Proton
3.51
1.82
1.39
7.21
42.36
1.00
2.39
2.20
8.740
8.721
8.115
8.095
8.076
7.248
2.056
1.518
1.498
1.257
0.851
0.834
-0.020
JEOL RESONANCE
Filename = AVZ-I-78-1_Proton-2-1.jdf
Author = delta
Experiment = proton.jxp
Sample_Id = AVZ-I-78-1
Solvent = CHLOROFORM-D
Creation_Time = 31-MAY-2017 11:28:54
Revision_Time = 31-MAY-2017 11:30:04
Current_Time = 31-MAY-2017 11:30:05
Comment = single_pulse
Data_Format = 1D COMPLEX
Dim_Size = 9942
Dim_Title = Proton
Dim_Units = [ppm]
Dimensions = X
Site = JNM-ECS400
Spectrometer = DELTA2_NMR
Field_Strength = 9.389766[T] (400[MHz])
X_Acq_Duration = 2.18365952[s]
X_Domain = 1H
X_Freq = 399.78219838[MHz]
X_Offset = 5[ppm]
X_Points = 16384
X_Prescans = 1
X_Resolution = 0.45794685[Hz]
X_Sweep = 7.5030012[kHz]
X_Sweep_Clipped = 6.00240096[kHz]
Irr_Domain = Proton
Irr_Freq = 399.78219838[MHz]
Irr_Offset = 5[ppm]
Tri_Domain = Proton
Tri_Freq = 399.78219838[MHz]
Tri_Offset = 5[ppm]
Clipped = FALSE
Scans = 8
Total_Scans = 8
Relaxation_Delay = 5[s]
Recvr_Gain = 42
Temp_Get = 20.4[dC]
X_90_Width = 11[us]
X_Acq_Time = 2.18365952[s]
X_Angle = 45[deg]
X_Atn = 0.99[dB]
X_Pulse = 5.5[us]
Irr_Mode = Off
Tri_Mode = Off
Dante_Presat = FALSE

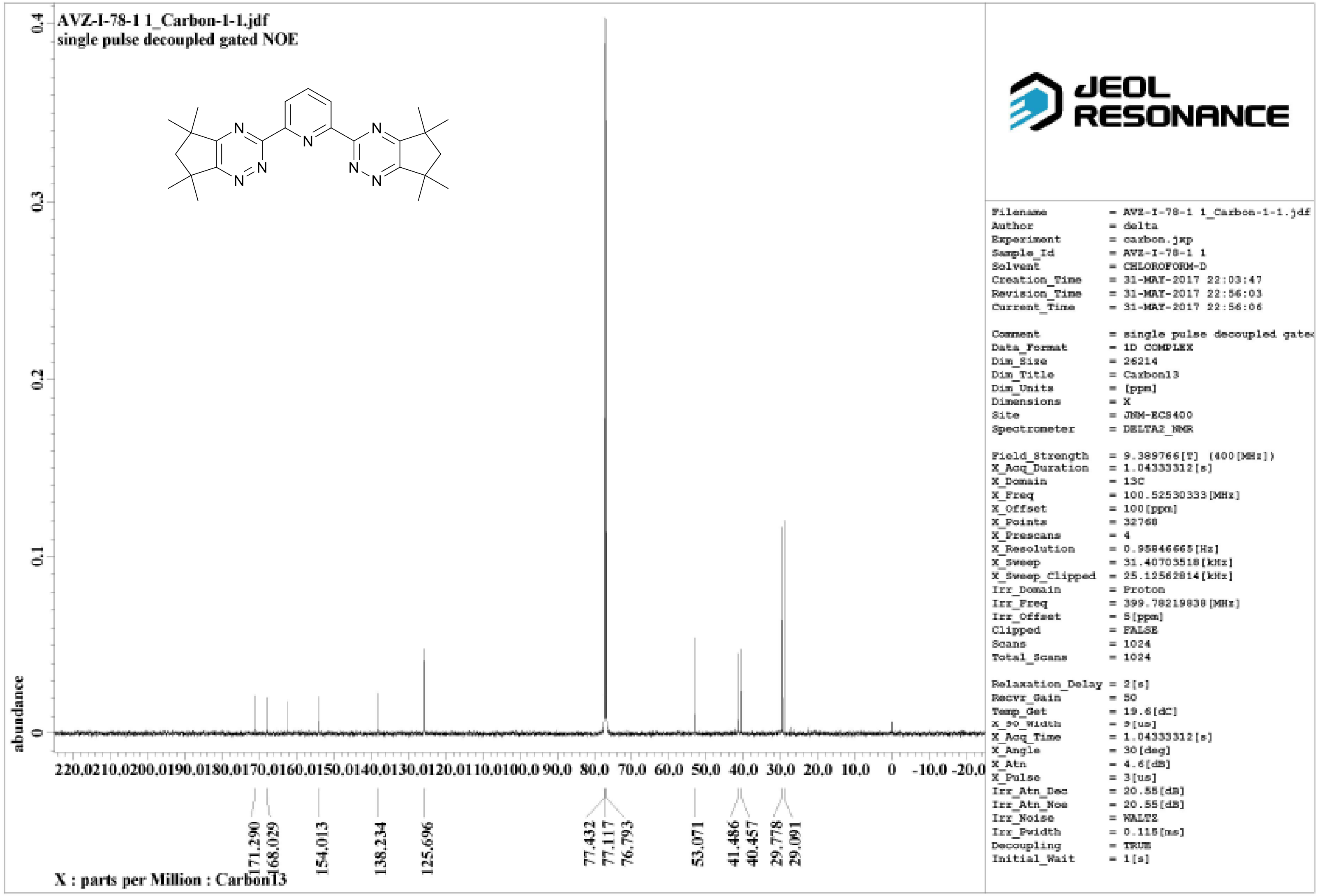

AVZ-I-78-1 1_Carbon-1-1.jdf
single pulse decoupled gated NOE
abundance
X : parts per Million : Carbon13
171.290
168.029
154.013
138.234
125.696
77.432
77.117
76.793
53.071
41.486
40.457
29.778
29.091
Filename = AVZ-I-78-1 1_Carbon-1-1.jdf
Author = delta
Experiment = carbon.jxp
Sample_Id = AVZ-I-78-1 1
Solvent = CHLOROFORM-D
Creation_Time = 31-MAY-2017 22:03:47
Revision_Time = 31-MAY-2017 22:56:03
Current_Time = 31-MAY-2017 22:56:06
Comment = single pulse decoupled gate
Data_Format = 1D COMPLEX
Dim_Size = 26214
Dim_Title = Carbon13
Dim_Units = [ppm]
Dimensions = X
Site = JNM-ECS400
Spectrometer = DELTA2_NMR
Field_Strength = 9.389766[T] (400[MHz])
X_Acq_Duration = 1.04333312[s]
X_Domain = 13C
X_Freq = 100.52530333[MHz]
X_Offset = 100[ppm]
X_Points = 32768
X_Prescans = 4
X_Resolution = 0.95846665[Hz]
X_Sweep = 31.40703518[kHz]
X_Sweep_Clipped = 25.12562814[kHz]
Irr_Domain = Proton
Irr_Freq = 399.78219838[MHz]
Irr_Offset = 5[ppm]
Clipped = FALSE
Scans = 1024
Total_Scans = 1024
Relaxation_Delay = 2[s]
Recvr_Gain = 50
Temp_Get = 19.6[dC]
X_90_Width = 9[us]
X_Acq_Time = 1.04333312[s]
X_Angle = 30[deg]
X_Atn = 4.6[dB]
X_Pulse = 3[us]
Irr_Atn_Dec = 20.55[dB]
Irr_Atn_Noe = 20.55[dB]
Irr_Noise = WALTZ
Irr_Pwidth = 0.115[ms]
Decoupling = TRUE
Initial_Wait = 1[s]

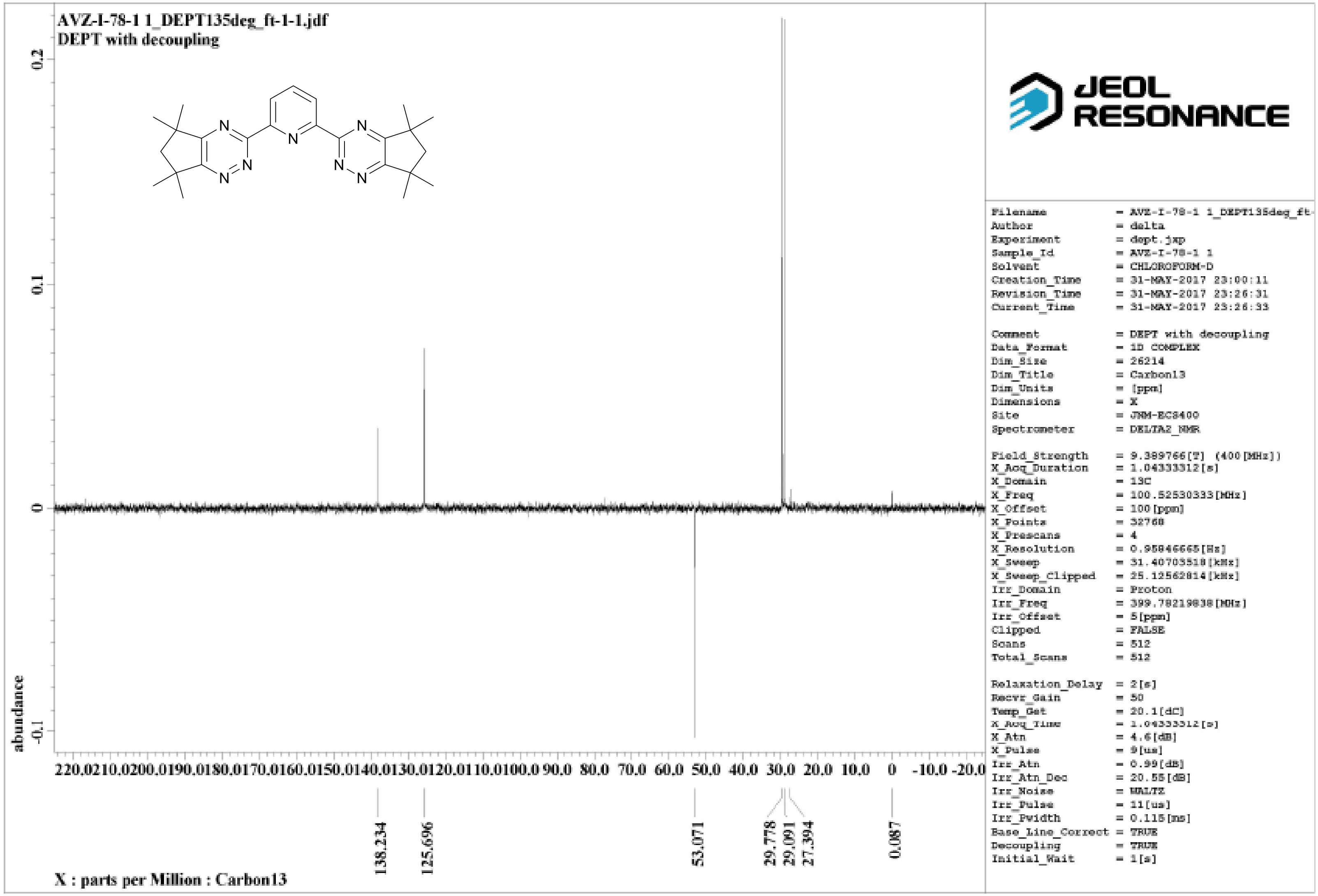
AVZ-I-78-1 1_DEPT135deg_ft-1-1.jdf
DEPT with decoupling
JEOL RESONANCE
abundance
X : parts per Million : Carbon13
138.234
125.696
53.071
29.778
29.091
27.394
0.087
Filename = AVZ-I-78-1 1_DEPT135deg_ft-
Author = delta
Experiment = dept.jxp
Sample_Id = AVZ-I-78-1 1
Solvent = CHLOROFORM-D
Creation_Time = 31-MAY-2017 23:00:11
Revision_Time = 31-MAY-2017 23:26:31
Current_Time = 31-MAY-2017 23:26:33
Comment = DEPT with decoupling
Data_Format = 1D COMPLEX
Dim_Size = 26214
Dim_Title = Carbon13
Dim_Units = [ppm]
Dimensions = X
Site = JNM-ECS400
Spectrometer = DELTA2_NMR
Field_Strength = 9.389766[T] (400[MHz])
X_Acq_Duration = 1.04333312[s]
X_Domain = 13C
X_Freq = 100.52530333[MHz]
X_Offset = 100[ppm]
X_Points = 32768
X_Prescans = 4
X_Resolution = 0.95846665[Hz]
X_Sweep = 31.40703518[kHz]
X_Sweep_Clipped = 25.12562814[kHz]
Irr_Domain = Proton
Irr_Freq = 399.78219838[MHz]
Irr_Offset = 5[ppm]
Clipped = FALSE
Scans = 512
Total_Scans = 512
Relaxation_Delay = 2[s]
Recvr_Gain = 50
Temp_Get = 20.1[dC]
X_Acq_Time = 1.04333312[s]
X_Atn = 4.6[dB]
X_Pulse = 9[us]
Irr_Atn = 0.99[dB]
Irr_Atn_Dec = 20.55[dB]
Irr_Noise = WALTZ
Irr_Pulse = 11[us]
Irr_Pwidth = 0.115[ms]
Base_Line_Correct = TRUE
Decoupling = TRUE
Initial_Wait = 1[s]

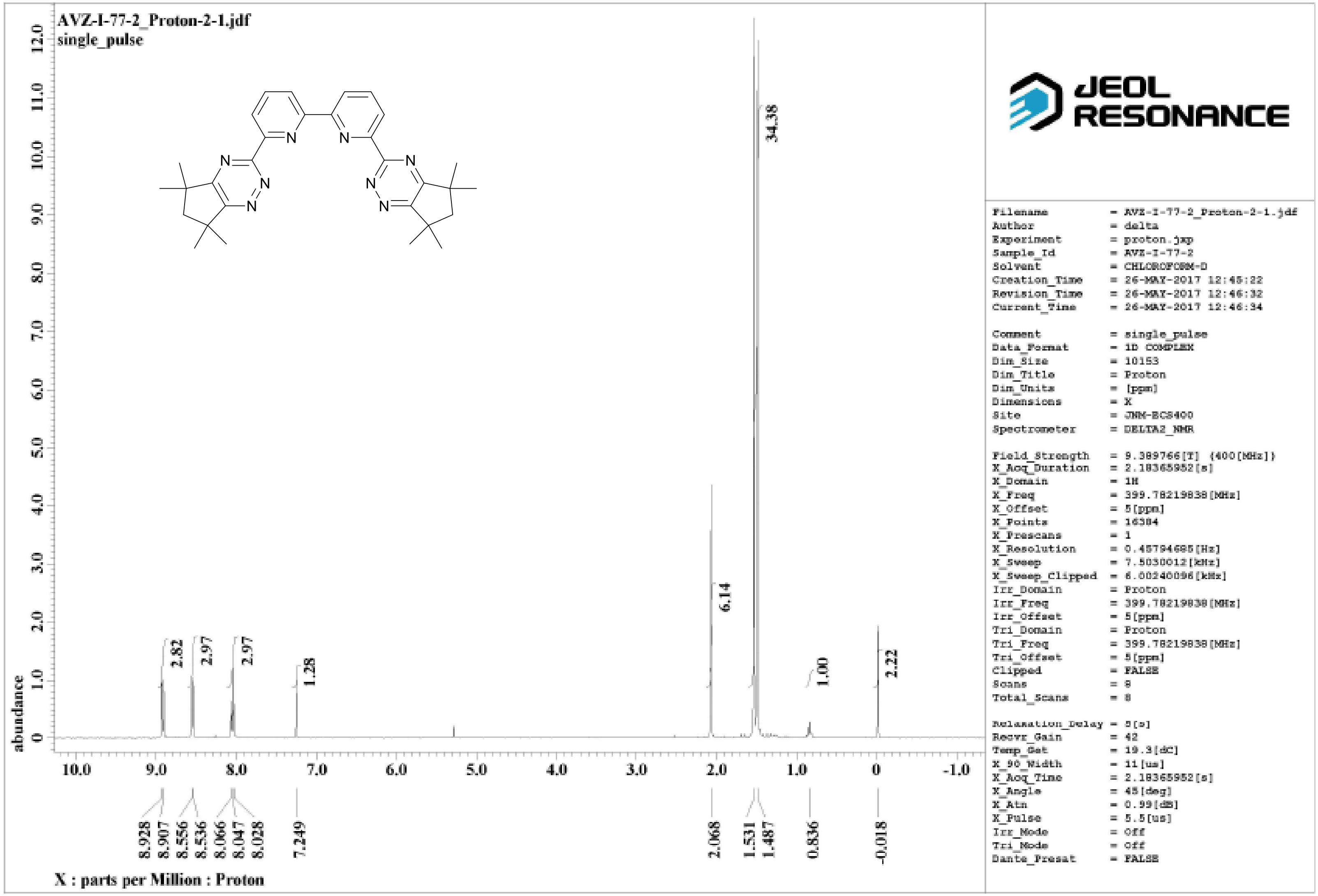
AVZ-I-77-2_Proton-2-1.jdf
single_pulse
N
N
N
N
N
N
N
N
abundance
12.0
11.0
10.0
9.0
8.0
7.0
6.0
5.0
4.0
3.0
2.0
1.0
0
2.82
2.97
2.97
1.28
6.14
34.38
1.00
2.22
10.0
9.0
8.0
7.0
6.0
5.0
4.0
3.0
2.0
1.0
0
-1.0
8.928
8.907
8.556
8.536
8.066
8.047
8.028
7.249
2.068
1.531
1.487
0.836
-0.018
X : parts per Million : Proton
JEOL RESONANCE
Filename = AVZ-I-77-2_Proton-2-1.jdf
Author = delta
Experiment = proton.jxp
Sample_Id = AVZ-I-77-2
Solvent = CHLOROFORM-D
Creation_Time = 26-MAY-2017 12:45:22
Revision_Time = 26-MAY-2017 12:46:32
Current_Time = 26-MAY-2017 12:46:34
Comment = single_pulse
Data_Format = 1D COMPLEX
Dim_Size = 10153
Dim_Title = Proton
Dim_Units = [ppm]
Dimensions = X
Site = JNM-ECS400
Spectrometer = DELTA2_NMR
Field_Strength = 9.389766[T] (400[MHz])
X_Acq_Duration = 2.18365952[s]
X_Domain = 1H
X_Freq = 399.78219838[MHz]
X_Offset = 5[ppm]
X_Points = 16384
X_Prescans = 1
X_Resolution = 0.45794685[Hz]
X_Sweep = 7.5030012[kHz]
X_Sweep_Clipped = 6.00240096[kHz]
Irr_Domain = Proton
Irr_Freq = 399.78219838[MHz]
Irr_Offset = 5[ppm]
Tri_Domain = Proton
Tri_Freq = 399.78219838[MHz]
Tri_Offset = 5[ppm]
Clipped = FALSE
Scans = 8
Total_Scans = 8
Relaxation_Delay = 5[s]
Recvr_Gain = 42
Temp_Get = 19.3[dC]
X_90_Width = 11[us]
X_Acq_Time = 2.18365952[s]
X_Angle = 45[deg]
X_Atn = 0.99[dB]
X_Pulse = 5.5[us]
Irr_Mode = Off
Tri_Mode = Off
Dante_Presat = FALSE

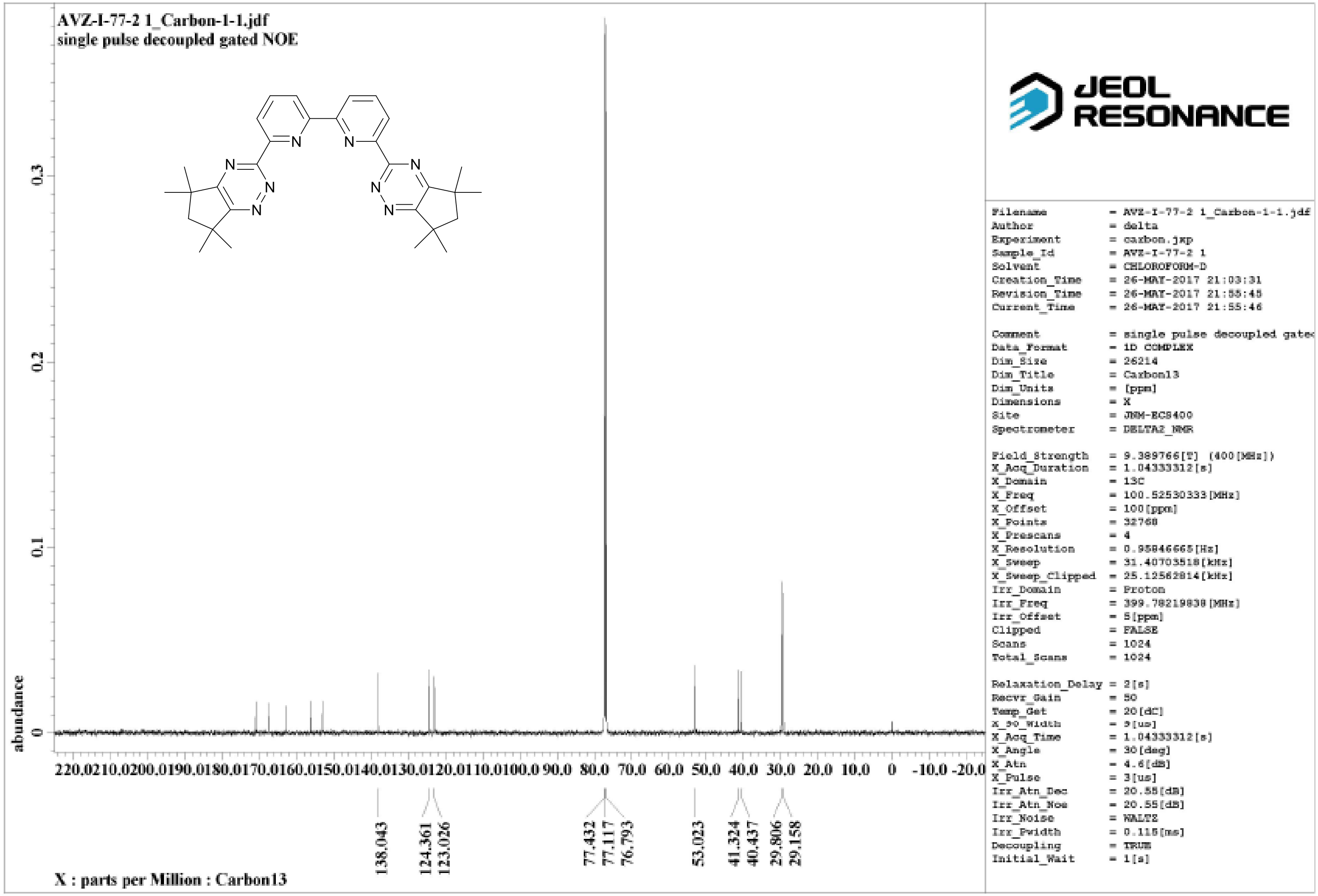
AVZ-I-77-2 1_Carbon-1-1.jdf
single pulse decoupled gated NOE
N
N
N
N
N
N
N
N
abundance
0
0.1
0.2
0.3
220.0 210.0 200.0 190.0 180.0 170.0 160.0 150.0 140.0 130.0 120.0 110.0 100.0 90.0 80.0 70.0 60.0 50.0 40.0 30.0 20.0 10.0 0 -10.0 -20.0
138.043
124.361
123.026
77.432
77.117
76.793
53.023
41.324
40.437
29.806
29.158
X : parts per Million : Carbon13
Filename = AVZ-I-77-2 1_Carbon-1-1.jdf
Author = delta
Experiment = carbon.jxp
Sample_Id = AVZ-I-77-2 1
Solvent = CHLOROFORM-D
Creation_Time = 26-MAY-2017 21:03:31
Revision_Time = 26-MAY-2017 21:55:45
Current_Time = 26-MAY-2017 21:55:46
Comment = single pulse decoupled gatec
Data_Format = 1D COMPLEX
Dim_Size = 26214
Dim_Title = Carbon13
Dim_Units = [ppm]
Dimensions = X
Site = JNM-ECS400
Spectrometer = DELTA2_NMR
Field_Strength = 9.389766[T] (400[MHz])
X_Acq_Duration = 1.04333312[s]
X_Domain = 13C
X_Freq = 100.52530333[MHz]
X_Offset = 100[ppm]
X_Points = 32768
X_Prescans = 4
X_Resolution = 0.95846665[Hz]
X_Sweep = 31.40703518[kHz]
X_Sweep_Clipped = 25.12562814[kHz]
Irr_Domain = Proton
Irr_Freq = 399.78219838[MHz]
Irr_Offset = 5[ppm]
Clipped = FALSE
Scans = 1024
Total_Scans = 1024
Relaxation_Delay = 2[s]
Recvr_Gain = 50
Temp_Get = 20[dC]
X_90_Width = 9[us]
X_Acq_Time = 1.04333312[s]
X_Angle = 30[deg]
X_Atn = 4.6[dB]
X_Pulse = 3[us]
Irr_Atn_Dec = 20.55[dB]
Irr_Atn_Noe = 20.55[dB]
Irr_Noise = WALTZ
Irr_Pwidth = 0.115[ms]
Decoupling = TRUE
Initial_Wait = 1[s]

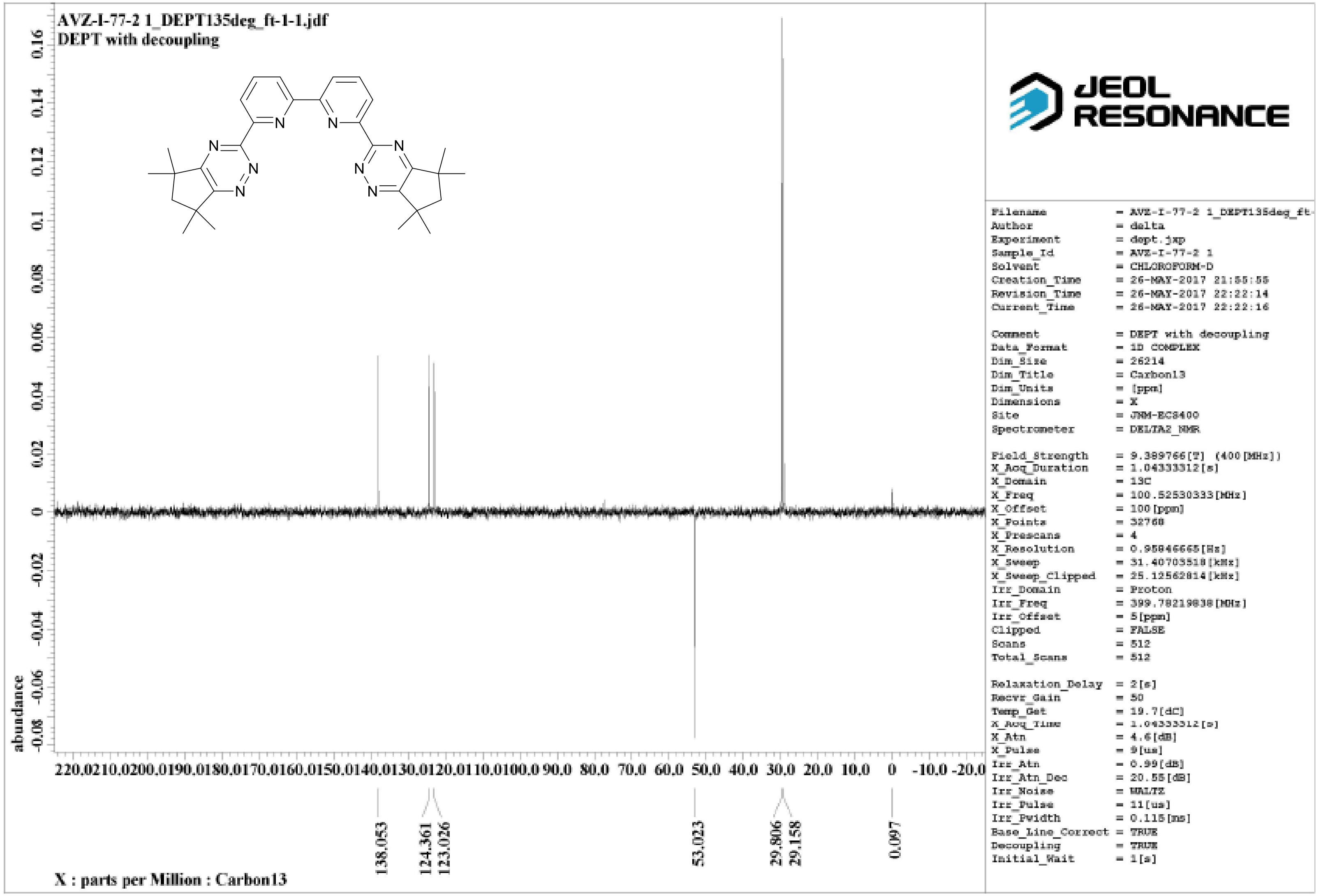
AVZ-I-77-2 1_DEPT135deg_ft-1-1.jdf
DEPT with decoupling
JEOL RESONANCE
Filename = AVZ-I-77-2 1_DEPT135deg_ft-
Author = delta
Experiment = dept.jxp
Sample_Id = AVZ-I-77-2 1
Solvent = CHLOROFORM-D
Creation_Time = 26-MAY-2017 21:55:55
Revision_Time = 26-MAY-2017 22:22:14
Current_Time = 26-MAY-2017 22:22:16
Comment = DEPT with decoupling
Data_Format = 1D COMPLEX
Dim_Size = 26214
Dim_Title = Carbon13
Dim_Units = [ppm]
Dimensions = X
Site = JNM-ECS400
Spectrometer = DELTA2_NMR
Field_Strength = 9.389766[T] (400[MHz])
X_Acq_Duration = 1.04333312[s]
X_Domain = 13C
X_Freq = 100.52530333[MHz]
X_Offset = 100[ppm]
X_Points = 32768
X_Prescans = 4
X_Resolution = 0.95846665[Hz]
X_Sweep = 31.40703518[kHz]
X_Sweep_Clipped = 25.12562814[kHz]
Irr_Domain = Proton
Irr_Freq = 399.78219838[MHz]
Irr_Offset = 5[ppm]
Clipped = FALSE
Scans = 512
Total_Scans = 512
Relaxation_Delay = 2[s]
Recvr_Gain = 50
Temp_Get = 19.7[dC]
X_Acq_Time = 1.04333312[s]
X_Atn = 4.6[dB]
X_Pulse = 9[us]
Irr_Atn = 0.99[dB]
Irr_Atn_Dec = 20.55[dB]
Irr_Noise = WALTZ
Irr_Pulse = 11[us]
Irr_Pwidth = 0.115[ms]
Base_Line_Correct = TRUE
Decoupling = TRUE
Initial_Wait = 1[s]
abundance
138.053
124.361
123.026
53.023
29.806
29.158
0.097
X : parts per Million : Carbon13

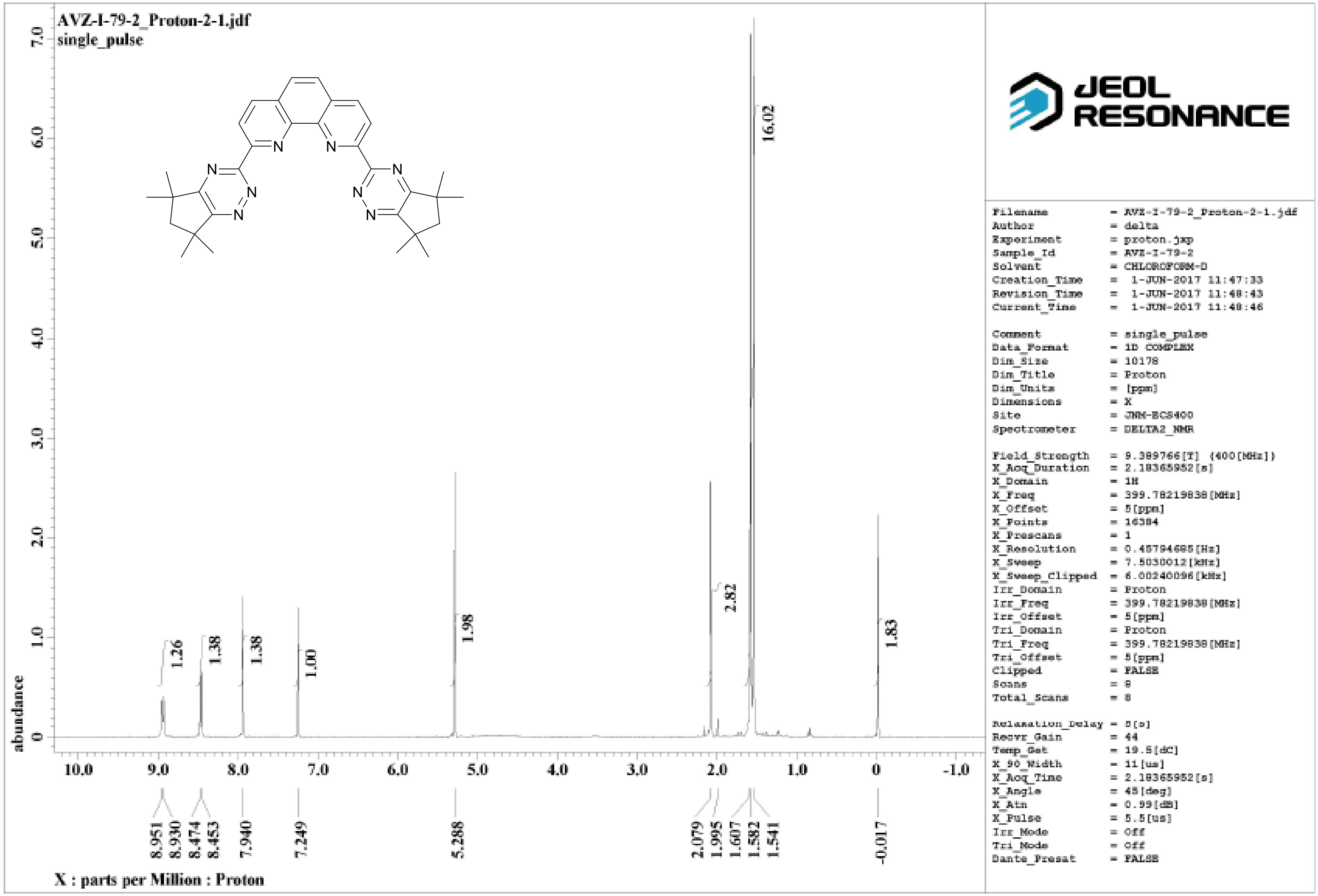
AVZ-I-79-2_Proton-2-1.jdf
single_pulse
abundance
X : parts per Million : Proton
1.26
1.38
1.38
1.00
1.98
2.82
16.02
1.83
8.951
8.930
8.474
8.453
7.940
7.249
5.288
2.079
1.995
1.607
1.582
1.541
-0.017
JEOL RESONANCE
Filename = AVZ-I-79-2_Proton-2-1.jdf
Author = delta
Experiment = proton.jxp
Sample_Id = AVZ-I-79-2
Solvent = CHLOROFORM-D
Creation_Time = 1-JUN-2017 11:47:33
Revision_Time = 1-JUN-2017 11:48:43
Current_Time = 1-JUN-2017 11:48:46
Comment = single_pulse
Data_Format = 1D COMPLEX
Dim_Size = 10178
Dim_Title = Proton
Dim_Units = [ppm]
Dimensions = X
Site = JNM-ECS400
Spectrometer = DELTA2_NMR
Field_Strength = 9.389766[T] (400[MHz])
X_Acq_Duration = 2.18365952[s]
X_Domain = 1H
X_Freq = 399.78219838[MHz]
X_Offset = 5[ppm]
X_Points = 16384
X_Prescans = 1
X_Resolution = 0.45794685[Hz]
X_Sweep = 7.5030012[kHz]
X_Sweep_Clipped = 6.00240096[kHz]
Irr_Domain = Proton
Irr_Freq = 399.78219838[MHz]
Irr_Offset = 5[ppm]
Tri_Domain = Proton
Tri_Freq = 399.78219838[MHz]
Tri_Offset = 5[ppm]
Clipped = FALSE
Scans = 8
Total_Scans = 8
Relaxation_Delay = 5[s]
Recvr_Gain = 44
Temp_Get = 19.5[dC]
X_90_Width = 11[us]
X_Acq_Time = 2.18365952[s]
X_Angle = 45[deg]
X_Atn = 0.99[dB]
X_Pulse = 5.5[us]
Irr_Mode = Off
Tri_Mode = Off
Dante_Presat = FALSE

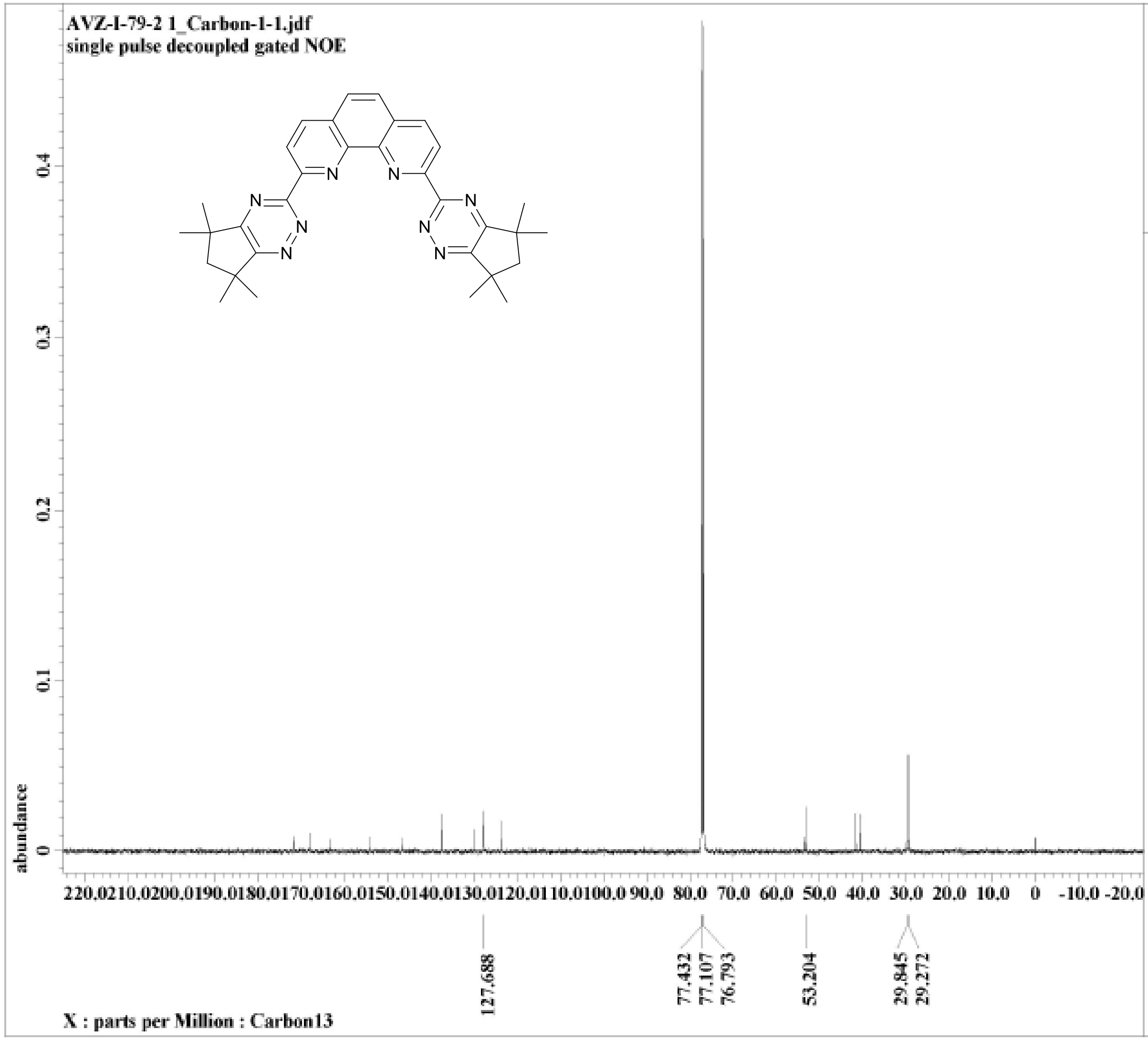

Filename	= AVZ-I-79-2 1_Carbon-1-1.jdf
Author	= delta
Experiment	= carbon.jxp
Sample_Id	= AVZ-I-79-2 1
Solvent	= CHLOROFORM-D
Creation_Time	= 1-JUN-2017 22:02:39
Revision_Time	= 1-JUN-2017 22:54:52
Current_Time	= 1-JUN-2017 22:54:55
Comment	= single pulse decoupled gate
Data_Format	= 1D COMPLEX
Dim_Size	= 26214
Dim_Title	= Carbon13
Dim_Units	= [ppm]
Dimensions	= X
Site	= JNM-ECS400
Spectrometer	= DELTA2_NMR
Field_Strength	= 9.389766[T] (400[MHz])
X_Acq_Duration	= 1.04333312[s]
X_Domain	= 13C
X_Freq	= 100.52530333[MHz]
X_Offset	= 100[ppm]
X_Points	= 32768
X_Prescans	= 4
X_Resolution	= 0.95846665[Hz]
X_Sweep	= 31.40703518[kHz]
X_Sweep_Clipped	= 25.12562814[kHz]
Irr_Domain	= Proton
Irr_Freq	= 399.78219838[MHz]
Irr_Offset	= 5[ppm]
Clipped	= FALSE
Scans	= 1024
Total_Scans	= 1024
Relaxation_Delay	= 2[s]
Recvr_Gain	= 50
Temp_Get	= 19.9[dC]
X_90_Width	= 9[us]
X_Acq_Time	= 1.04333312[s]
X_Angle	= 30[deg]
X_Atn	= 4.6[dB]
X_Pulse	= 3[us]
Irr_Atn_Dec	= 20.55[dB]
Irr_Atn_Noe	= 20.55[dB]
Irr_Noise	= WALTZ
Irr_Pwidth	= 0.115[ms]
Decoupling	= TRUE
Initial_Wait	= 1[s]

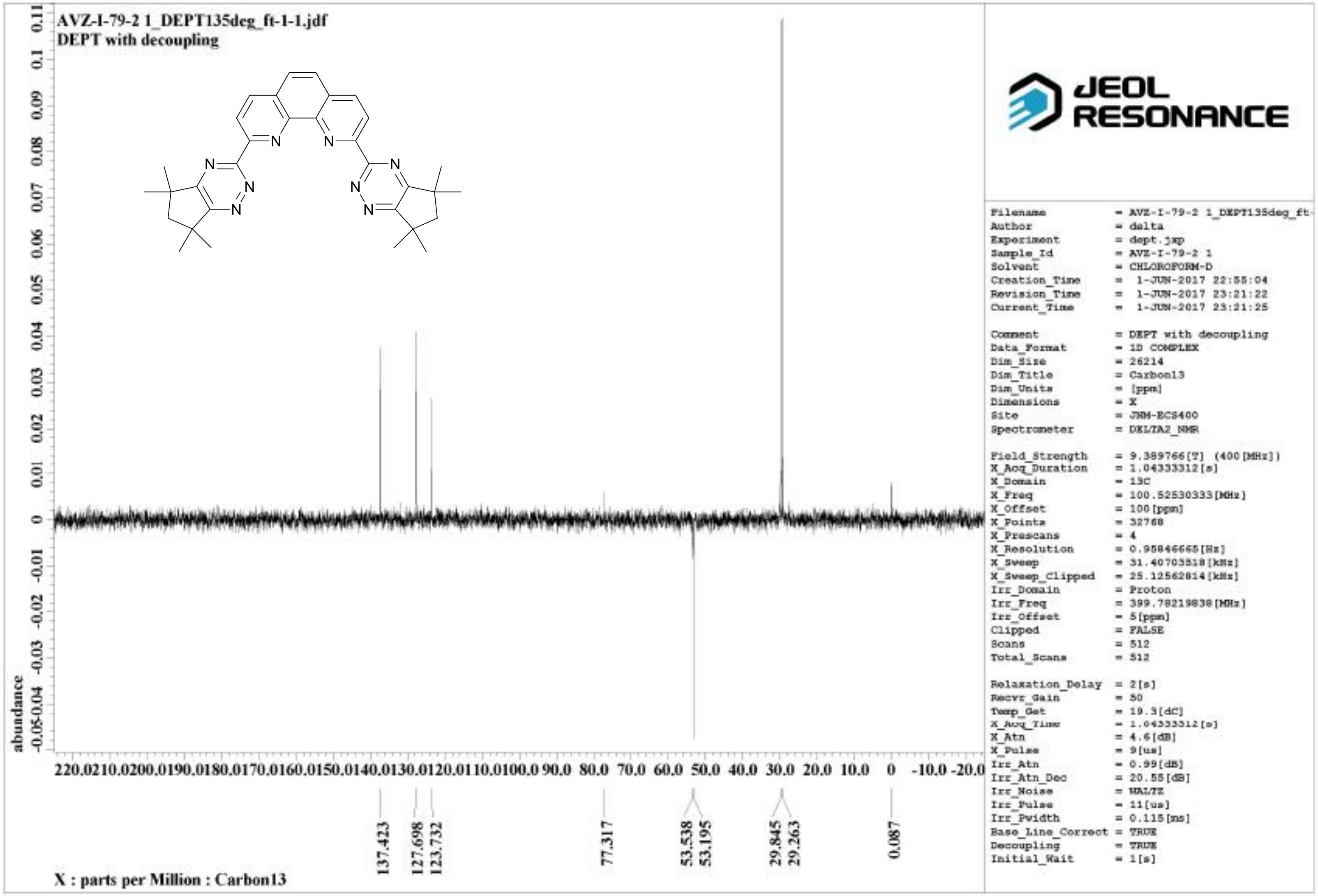

AVZ-I-79-2 1_DEPT135deg_ft-1-1.jdf
DEPT with decoupling
N
N
N
N
N
N
N
N
abundance
0.11
0.1
0.09
0.08
0.07
0.06
0.05
0.04
0.03
0.02
0.01
0
-0.01
-0.02
-0.03
-0.04
-0.05
220.0
210.0
200.0
190.0
180.0
170.0
160.0
150.0
140.0
130.0
120.0
110.0
100.0
90.0
80.0
70.0
60.0
50.0
40.0
30.0
20.0
10.0
0
-10.0
-20.0
137.423
127.698
123.732
77.317
53.538
53.195
29.845
29.263
0.087
X : parts per Million : Carbon13
JEOL RESONANCE
Filename = AVZ-I-79-2 1_DEPT135deg_ft-
Author = delta
Experiment = dept.jxp
Sample_Id = AVZ-I-79-2 1
Solvent = CHLOROFORM-D
Creation_Time = 1-JUN-2017 22:55:04
Revision_Time = 1-JUN-2017 23:21:22
Current_Time = 1-JUN-2017 23:21:25
Comment = DEPT with decoupling
Data_Format = 1D COMPLEX
Dim_Size = 26214
Dim_Title = Carbon13
Dim_Units = [ppm]
Dimensions = X
Site = JNM-ECS400
Spectrometer = DELTA2_NMR
Field_Strength = 9.389766[T] (400[MHz])
X_Acq_Duration = 1.04333312[s]
X_Domain = 13C
X_Freq = 100.52530333[MHz]
X_Offset = 100[ppm]
X_Points = 32768
X_Prescans = 4
X_Resolution = 0.95846665[Hz]
X_Sweep = 31.40703518[kHz]
X_Sweep_Clipped = 25.12562814[kHz]
Irr_Domain = Proton
Irr_Freq = 399.78219838[MHz]
Irr_Offset = 5[ppm]
Clipped = FALSE
Scans = 512
Total_Scans = 512
Relaxation_Delay = 2[s]
Recvr_Gain = 50
Temp_Get = 19.3[dC]
X_Acq_Time = 1.04333312[s]
X_Atn = 4.6[dB]
X_Pulse = 9[us]
Irr_Atn = 0.99[dB]
Irr_Atn_Dec = 20.55[dB]
Irr_Noise = WALTZ
Irr_Pulse = 11[us]
Irr_Pwidth = 0.115[ms]
Base_Line_Correct = TRUE
Decoupling = TRUE
Initial_Wait = 1[s]

3: X-ray Crystallography

3.1 Crystal Data and Structure Refinement

Table 1. Crystal data and structure refinement for Y(**10**)(NO_3)$_3$.

Empirical formula	$C_{36}H_{40}N_{13}O_9Y$
Formula weight	887.72
Temperature/K	150.0(2)
Crystal system	triclinic
Space group	P-1
a/Å	11.5205(4)
b/Å	11.7850(4)
c/Å	15.5829(6)
α/°	82.476(3)
β/°	78.915(3)
γ/°	84.917(3)
Volume/Å^3	2053.95(13)
Z	2
ρ_{calc}g/cm^3	1.435
μ/mm^{-1}	1.488
F(000)	916.0
Crystal size/mm^3	0.38 × 0.12 × 0.06
Radiation	MoKα (λ = 0.71073)
2Θ range for data collection/°	6.736 to 57.598
Index ranges	$-15 \leq h \leq 15$, $-15 \leq k \leq 15$, $-20 \leq l \leq 20$
Reflections collected	33090
Independent reflections	9307 [R_{int} = 0.0428, R_{sigma} = 0.0522]
Data/restraints/parameters	9307/0/542
Goodness-of-fit on F^2	1.028
Final R indexes [I>=2σ (I)]	R_1 = 0.0374, wR_2 = 0.0665
Final R indexes [all data]	R_1 = 0.0559, wR_2 = 0.0719
Largest diff. peak/hole / e Å^{-3}	0.35/-0.42

Table 2. Crystal data and structure refinement for Lu(**10**)(NO_3)$_3$.

Empirical formula	$C_{36}H_{40}LuN_{13}O_9$
Formula weight	973.78
Temperature/K	150.0(2)
Crystal system	triclinic
Space group	P-1
a/Å	11.5908(3)
b/Å	11.6261(2)
c/Å	15.7382(3)
α/°	82.7580(18)
β/°	78.147(2)
γ/°	84.630(2)
Volume/Å^3	2054.03(9)
Z	2
ρ_{calc}g/cm^3	1.574
μ/mm^{-1}	2.472
F(000)	980.0
Crystal size/mm^3	0.36 × 0.2 × 0.04
Radiation	MoKα (λ = 0.71073)
2Θ range for data collection/°	6.708 to 58.872
Index ranges	-14 ≤ h ≤ 15, -16 ≤ k ≤ 15, -20 ≤ l ≤ 21
Reflections collected	66921
Independent reflections	10228 [R_{int} = 0.0532, R_{sigma} = 0.0415]
Data/restraints/parameters	10228/0/542
Goodness-of-fit on F^2	1.050
Final R indexes [I>=2σ (I)]	R_1 = 0.0304, wR_2 = 0.0546
Final R indexes [all data]	R_1 = 0.0423, wR_2 = 0.0589
Largest diff. peak/hole / e Å^{-3}	1.49/-0.72

Table 3. Crystal data and structure refinement for $[Pr(\mathbf{8})_2(NO_3)][Pr(NO_3)_5]$.

Empirical formula	$C_{62}H_{72}N_{23}O_{19}Pr_2$
Formula weight	1725.24
Temperature/K	150.0(2)
Crystal system	monoclinic
Space group	C2/c
a/Å	32.0214(3)
b/Å	37.2925(3)
c/Å	26.8722(3)
α/°	90
β/°	96.9857(9)
γ/°	90
Volume/Å^3	31851.5(5)
Z	16
ρ_{calc}g/cm^3	1.439
μ/mm^{-1}	1.287
F(000)	14000.0
Crystal size/mm^3	0.29 × 0.24 × 0.15
Radiation	MoKα (λ = 0.71073)
2Θ range for data collection/°	6.486 to 57.628
Index ranges	$-39 \le h \le 40$, $-48 \le k \le 49$, $-34 \le l \le 33$
Reflections collected	253698
Independent reflections	37958 [R_{int} = 0.0510, R_{sigma} = 0.0457]
Data/restraints/parameters	37958/3430/2052
Goodness-of-fit on F^2	1.012
Final R indexes [I>=2σ (I)]	R_1 = 0.0429, wR_2 = 0.0979
Final R indexes [all data]	R_1 = 0.0713, wR_2 = 0.1124
Largest diff. peak/hole / e Å^{-3}	1.74/-2.10

Table 4. Crystal data and structure refinement for **8**.

Empirical formula	$C_{30}H_{34}N_8$
Formula weight	506.65
Temperature/K	150.0(2)
Crystal system	monoclinic
Space group	$P2_1/n$
a/Å	8.1747(3)
b/Å	17.1489(5)
c/Å	10.1387(4)
α/°	90
β/°	110.859(4)
γ/°	90
Volume/Å^3	1328.15(8)
Z	2
ρ_{calc}g/cm^3	1.267
μ/mm^{-1}	0.079
F(000)	540.0
Crystal size/mm^3	0.42 × 0.34 × 0.1
Radiation	MoKα (λ = 0.71073)
2Θ range for data collection/°	7.144 to 57.508
Index ranges	-9 ≤ h ≤ 10, -21 ≤ k ≤ 22, -13 ≤ l ≤ 13
Reflections collected	20888
Independent reflections	3137 [R_{int} = 0.0417, R_{sigma} = 0.0321]
Data/restraints/parameters	3137/0/176
Goodness-of-fit on F^2	1.043
Final R indexes [I>=2σ (I)]	R_1 = 0.0451, wR_2 = 0.0966
Final R indexes [all data]	R_1 = 0.0646, wR_2 = 0.1068
Largest diff. peak/hole / e Å^{-3}	0.27/-0.22

3.2 Crystal Structures

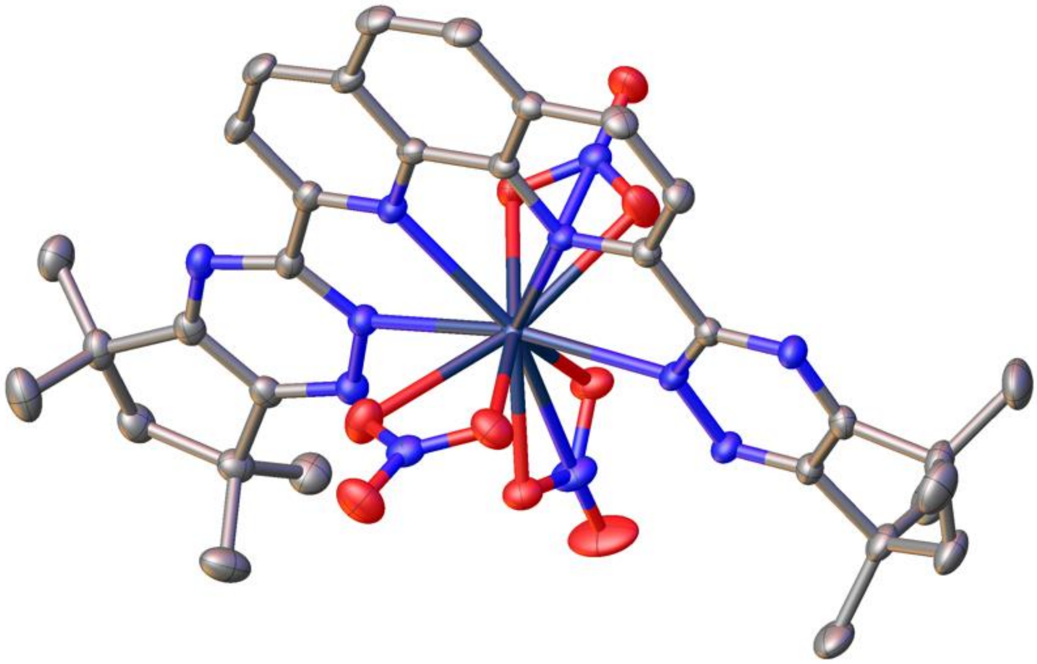

Figure 1. The asymmetric unit of the crystal structure of Y(**10**)$(NO_3)_3$ with thermal ellipsoids at the 50% probability level. Hydrogen atoms, solvent molecules and atomic numbering are omitted for clarity. CCDC 1891928 contains the crystallographic data for this structure. These data can be obtained free of charge via www.ccdc.cam.ac.uk/conts/retrieving.html, or from the Cambridge Crystallographic Data Centre, 12 Union Road, Cambridge CB2 1EZ, UK (fax: (+44)1223-336-033; or deposit@ccdc.cam.ac.uk).

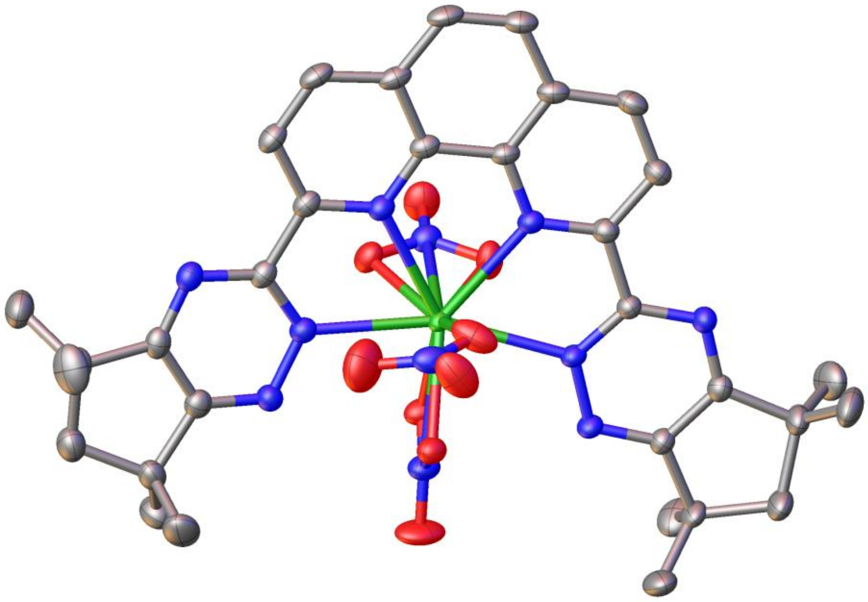

Figure 2. The asymmetric unit of the crystal structure of Lu(**10**)(NO_3)$_3$ with thermal ellipsoids at the 50% probability level. Hydrogen atoms, solvent molecules and atomic numbering are omitted for clarity. CCDC 1891927 contains the crystallographic data for this structure. These data can be obtained free of charge via www.ccdc.cam.ac.uk/conts/retrieving.html, or from the Cambridge Crystallographic Data Centre, 12 Union Road, Cambridge CB2 1EZ, UK (fax: (+44)1223-336-033; or deposit@ccdc.cam.ac.uk).

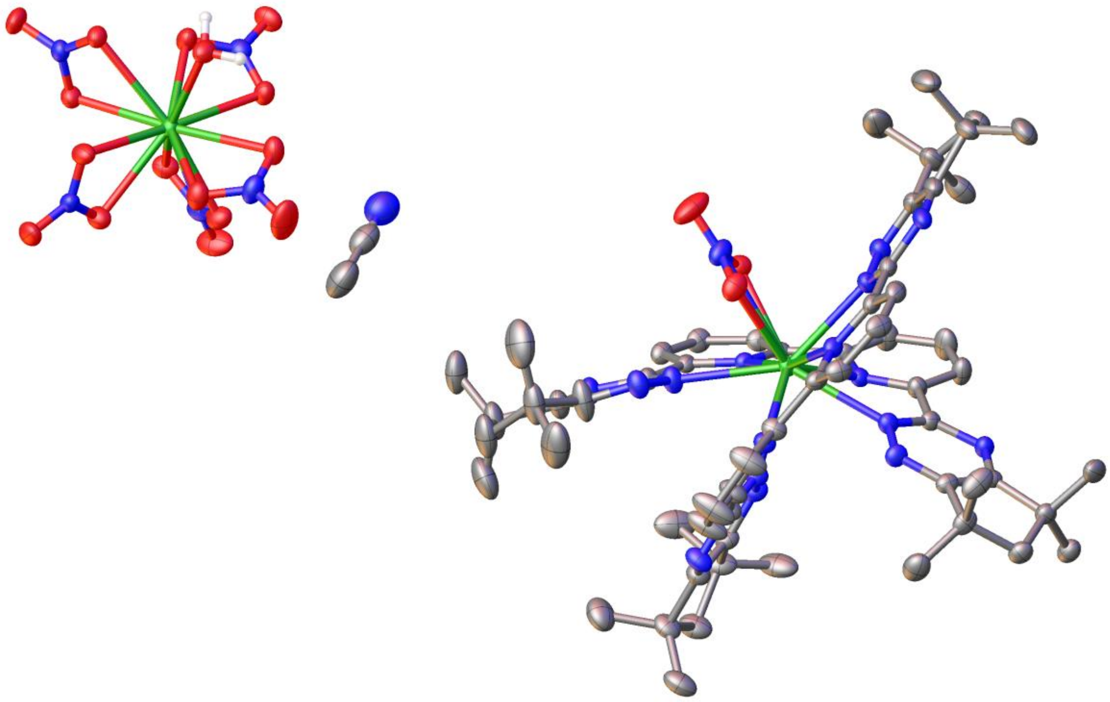

Figure 3. One crystallographically independent formula unit of the crystal structure of $[Pr(\mathbf{8})_2(NO_3)][Pr(NO_3)_5]$ with thermal ellipsoids at the 50% probability level. Hydrogen atoms bound to carbon and the atomic numbering scheme are omitted for clarity. CCDC 1891930 contains the crystallographic data for this structure. These data can be obtained free of charge via www.ccdc.cam.ac.uk/conts/retrieving.html, or from the Cambridge Crystallographic Data Centre, 12 Union Road, Cambridge CB2 1EZ, UK (fax: (+44)1223-336-033; or deposit@ccdc.cam.ac.uk).

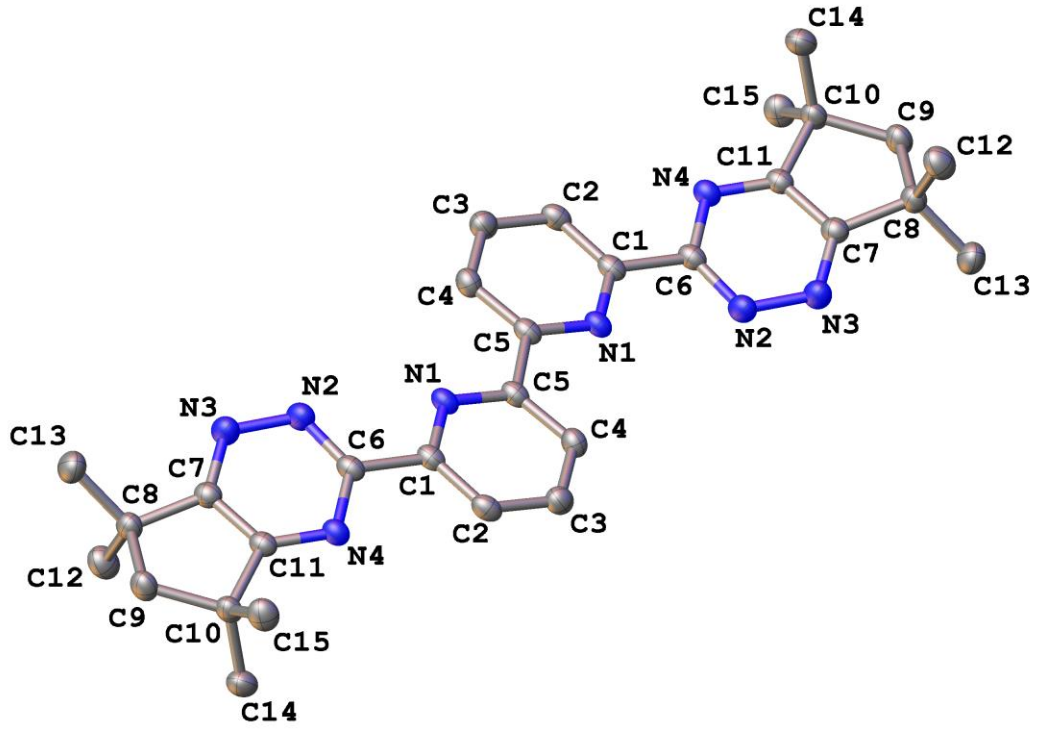

Figure 4. X-ray crystal structure of **8** with thermal ellipsoids at the 50% probability level. Hydrogen atoms and solvent molecules are omitted for clarity. CCDC 1891929 contains the crystallographic data for this structure. These data can be obtained free of charge via www.ccdc.cam.ac.uk/conts/retrieving.html, or from the Cambridge Crystallographic Data Centre, 12 Union Road, Cambridge CB2 1EZ, UK (fax: (+44)1223-336-033; or deposit@ccdc.cam.ac.uk).

4: Solvent Extraction Studies

4.1 Extraction Studies for Ligand 6

Table 5. Extraction of Am(III) and Eu(III) by ligand **6** as a function of the initial nitric acid concentration of the aqueous phase. Results are from gamma spectrometry (*D* = distribution ratio, SF = separation factor, contact time: 60 min, temperature: 22 °C ± 1 °C).

**[HNO$_3$] initial (mol/L)**	D_{Am}	D_{Eu}	$SF_{Am/Eu}$
0.01	0.016	0.001	26.27
0.11	0.019	0.002	8.60
0.30	0.067	0.008	7.99
0.70	0.277	0.031	9.01
1.03	0.427	0.050	8.61
3.11	1.565	0.038	41.00

Table 6. Extraction of Am(III) and Cm(III) by ligand **6** as a function of the initial nitric acid concentration of the aqueous phase. Results are from alpha spectrometry (*D* = distribution ratio, SF = separation factor, contact time: 60 min, temperature: 22 °C ± 1 °C).

**[HNO$_3$] initial (mol/L)**	D_{Am}	D_{Cm}	$SF_{Am/Cm}$
0.01	0.01	0.01	1.05
0.11	0.04	0.02	2.44
0.30	0.08	0.06	1.33
0.70	0.22	0.17	1.33
1.03	0.27	0.20	1.31
3.11	1.39	1.05	1.31

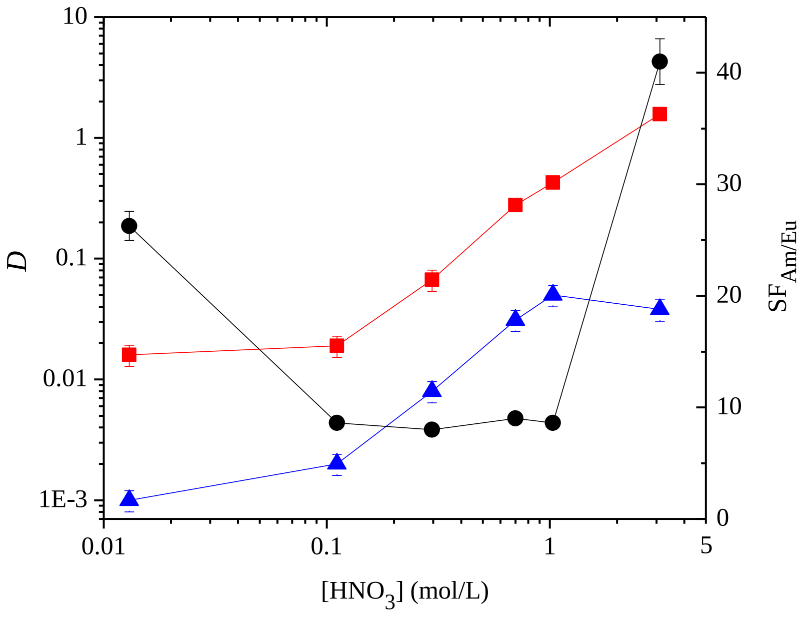

Figure 5. Extraction of Am(III) and Eu(III) by ligand **6** (0.01 M) into 1-octanol as a function of the initial nitric acid concentration of the aqueous phase (*D* = distribution ratio, SF = separation factor, ■ = D_{Am}, ▲ = D_{Eu}, ● = $SF_{Am/Eu}$, mixing time: 60 min., temperature: 22°C ± 1°C).

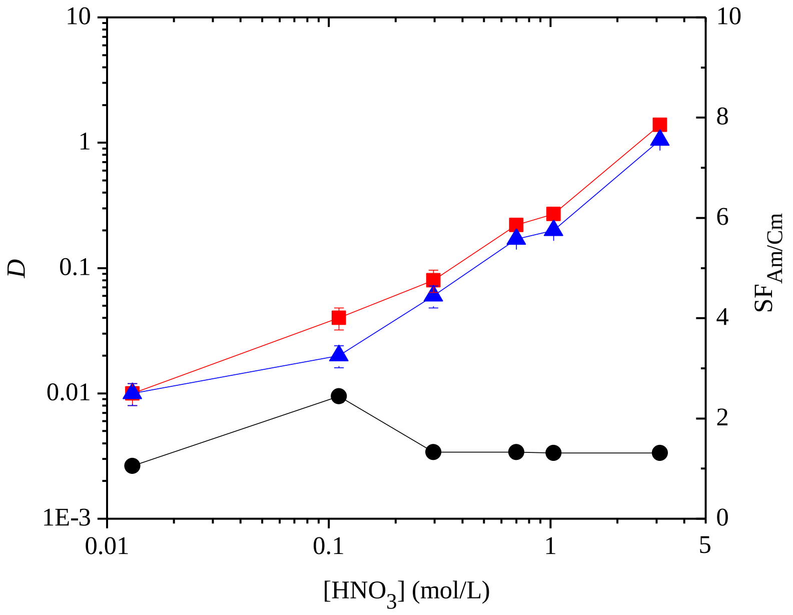

Figure 6. Extraction of Am(III) and Cm(III) by ligand **6** (0.01 M) into 1-octanol as a function of the initial nitric acid concentration of the aqueous phase (D = distribution ratio, SF = separation factor, ■ = D_{Am}, ▲ = D_{Cm}, ● = $SF_{Am/Cm}$, contact time: 60 min, temperature: 22 °C ± 1 °C).

Table 7. Extraction of Y(III) and all the trivalent lanthanides (except Pm) by ligand **6** as a function of the initial nitric acid concentration of the aqueous phase. Results are from ICP-MS (*D* = distribution ratio, SF = separation factor, contact time: 60 min, temperature: 22 °C ± 1 °C).

**[HNO_3] (mol/L)**		0.01	0.11	0.30	0.70	1.03	3.11
**Element**	**Atomic Number**	*D*	*D*	*D*	*D*	*D*	*D*
Y	39	0.004	0.001	0.001	0.003	0.002	0.005
La	57	0.002	0.001	0.001	0.001	0.001	0.002
Ce	58	0.003	0.001	0.001	0.001	0.001	0.002
Pr	59	0.001	0.000	0.000	0.000	0.000	0.002
Nd	60	0.002	0.001	0.001	0.001	0.001	0.003
Sm	62	0.001	0.001	0.005	0.013	0.007	0.018
Eu	63	0.001	0.002	0.010	0.027	0.016	0.042
Gd	64	0.001	0.002	0.009	0.026	0.016	0.043
Tb	65	0.003	0.002	0.012	0.036	0.022	0.077
Dy	66	0.005	0.002	0.011	0.034	0.021	0.077
Ho	67	0.004	0.002	0.009	0.028	0.016	0.063
Er	68	0.005	0.002	0.008	0.022	0.014	0.053
Tm	69	0.004	0.002	0.007	0.018	0.010	0.044
Yb	70	0.004	0.002	0.006	0.014	0.009	0.035
Lu	71	0.003	0.001	0.005	0.011	0.006	0.027

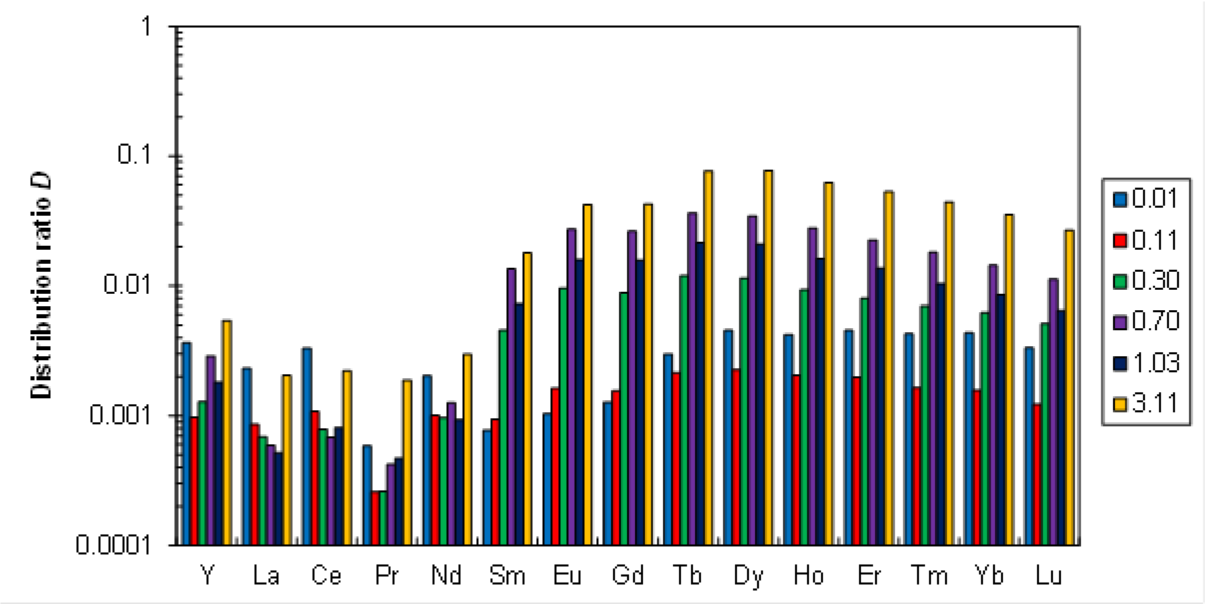

Figure 7. Extraction of Y(III) and all the trivalent lanthanides (except Pm) by ligand **6** as a function of the initial nitric acid concentration (in mol dm^{-3}) of the aqueous phase. Results are from ICP-MS (*D* = distribution ratio, SF = separation factor, contact time: 60 min, temperature: 22 ^{o}C ± 1 ^{o}C).

4.2 Extraction Studies for Ligand 8

Table 8. Extraction of Am(III) and Eu(III) by ligand **8** as a function of the initial nitric acid concentration of the aqueous phase. Results are from gamma spectrometry (*D* = distribution ratio, SF = separation factor, contact time: 60 min, temperature: 22 °C ± 1 °C).

**[HNO_3] initial (mol/L)**	D_{Am}	D_{Eu}	$SF_{Am/Eu}$
0.01	0.004	0.00031	12.50
0.11	0.012	0.00018	66.65
0.30	0.063	0.001	79.03
0.70	0.298	0.002	125.25
1.03	0.671	0.005	135.56
3.11	2.600	0.022	119.19

Table 9. Extraction of Am(III) and Cm(III) by ligand **8** as a function of the initial nitric acid concentration of the aqueous phase. Results are from alpha spectrometry (*D* = distribution ratio, SF = separation factor, contact time: 60 min, temperature: 22 °C ± 1 °C).

**[HNO_3] initial (mol/L)**	D_{Am}	D_{Cm}	$SF_{Am/Cm}$
0.01	0.007	0.004	1.73
0.11	0.035	0.009	3.94
0.30	0.055	0.032	1.73
0.70	0.257	0.147	1.76
1.03	0.817	0.478	1.71
3.11	1.846	1.257	1.47

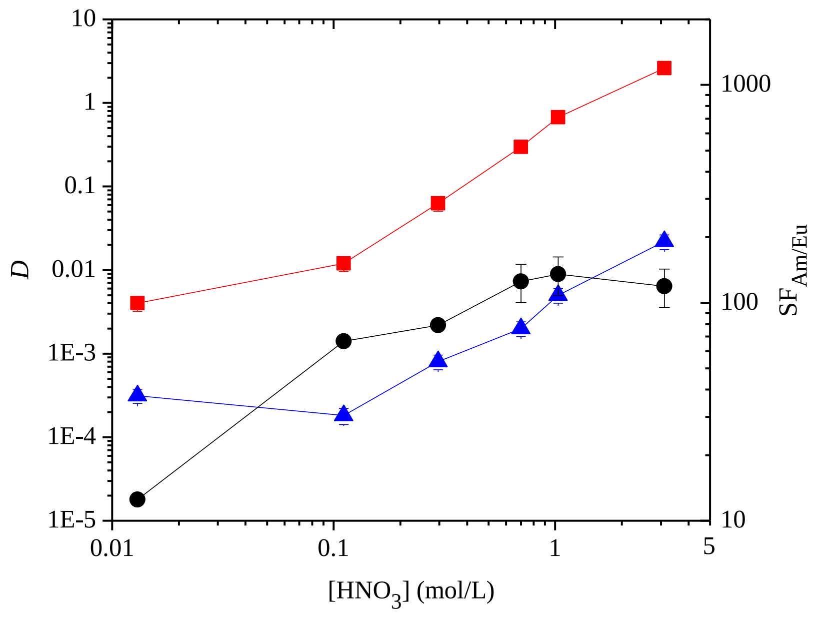

Figure 8. Extraction of Am(III) and Eu(III) by ligand **8** (0.01 M) in 1-octanol as a function of the initial nitric acid concentration of the aqueous phase (D = distribution ratio, SF = separation factor, ■ = D_{Am}, ▲ = D_{Eu}, ● = $SF_{Am/Eu}$, mixing time: 60 min., temperature: 22°C ± 1°C).

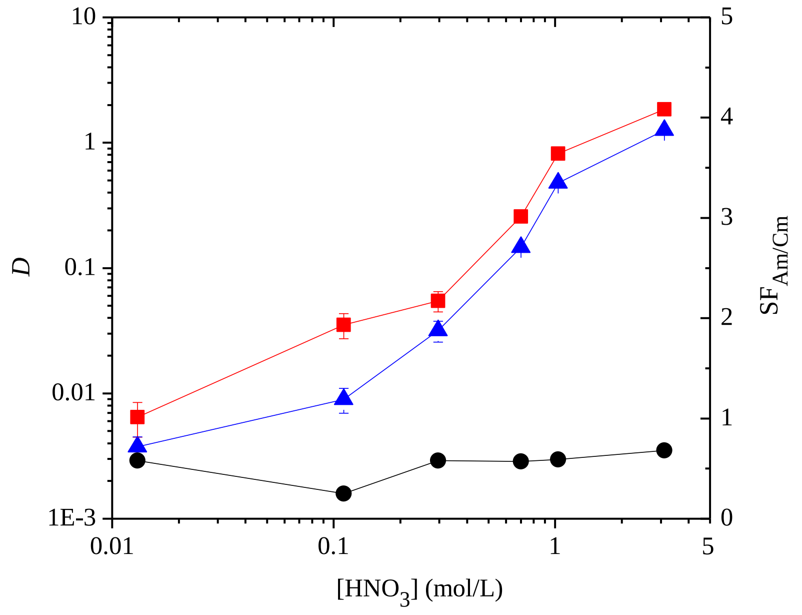

Figure 9. Extraction of Am(III) and Cm(III) by ligand **8** (0.01 M) into 1-octanol as a function of the initial nitric acid concentration of the aqueous phase (D = distribution ratio, SF = separation factor, ■ = D_{Am}, ▲ = D_{Cm}, ● = $SF_{Am/Cm}$, contact time: 60 min, temperature: 22 °C ± 1 °C).

Table 10. Extraction of Y(III) and all the trivalent lanthanides (except Pm) by ligand **8** as a function of the initial nitric acid concentration of the aqueous phase. Results are from ICP-MS (*D* = distribution ratio, SF = separation factor, contact time: 60 min, temperature: 22 ^{o}C ± 1 ^{o}C).

**[HNO$_3$] (mol/L)**		0.01	0.11	0.30	0.70	1.03	3.11
**Element**	**Atomic Number**	*D*	*D*	*D*	*D*	*D*	*D*
Y	39	0.001	0.001	0.001	0.001	0.003	0.015
La	57	0.001	0.001	0.001	0.001	0.001	0.001
Ce	58	0.002	0.001	0.001	0.001	0.004	0.002
Pr	59	0.001	0.001	0.000	0.001	0.001	0.003
Nd	60	0.002	0.002	0.001	0.002	0.001	0.007
Sm	62	0.002	0.001	0.001	0.003	0.006	0.022
Eu	63	0.001	0.001	0.001	0.003	0.007	0.032
Gd	64	0.001	0.001	0.001	0.003	0.008	0.036
Tb	65	0.002	0.001	0.002	0.009	0.023	0.133
Dy	66	0.002	0.001	0.003	0.017	0.047	0.284
Ho	67	0.002	0.001	0.005	0.028	0.074	0.371
Er	68	0.003	0.002	0.006	0.033	0.087	0.354
Tm	69	0.006	0.002	0.006	0.030	0.078	0.285
Yb	70	0.017	0.004	0.006	0.026	0.064	0.240
Lu	71	0.044	0.007	0.006	0.019	0.047	0.212

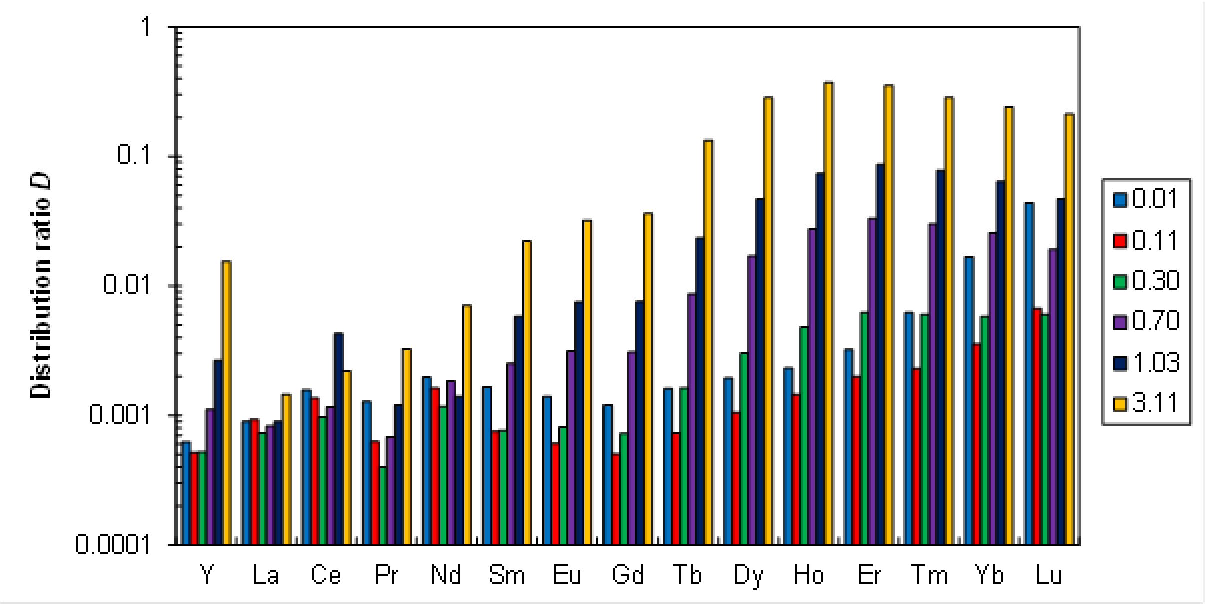

Figure 10. Extraction of Y(III) and all the trivalent lanthanides (except Pm) by ligand **8** as a function of the initial nitric acid concentration (in mol dm^{-3}) of the aqueous phase. Results are from ICP-MS (*D* = distribution ratio, SF = separation factor, contact time: 60 min, temperature: 22 °C ± 1 °C).

Table 11. Extraction of Am(III) and Eu(III) by ligand **8** + *N*,*N*'-dimethyl-*N*,*N*'-dioctyl-2-hexyloxyethyl malonamide **11** (0.25 M) as a function of the initial nitric acid concentration of the aqueous phase. Results are from gamma spectrometry (*D* = distribution ratio, SF = separation factor, contact time: 60 min, temperature: 22 ^{o}C ± 1 ^{o}C).

**[HNO$_3$] initial (mol/L)**	D_{Am}	D_{Eu}	$SF_{Am/Eu}$
0.001	0.100	0.010	10.13
0.01	0.033	0.003	9.66
0.11	0.046	0.003	13.84
1.03	1.490	0.026	56.97
2.009	2.976	0.054	54.95
4.12	1.825	0.093	19.64

Table 12. Extraction of Am(III) and Cm(III) by ligand **8** + *N*,*N*'-dimethyl-*N*,*N*'-dioctyl-2-hexyloxyethyl malonamide **11** (0.25 M) as a function of the initial nitric acid concentration of the aqueous phase. Results are from alpha spectrometry (*D* = distribution ratio, SF = separation factor, contact time: 60 min, temperature: 22 ^{o}C ± 1 ^{o}C).

**[HNO$_3$] initial (mol/L)**	D_{Am}	D_{Cm}	$SF_{Am/Cm}$
0.001	0.11	0.06	1.73
0.01	0.04	0.02	1.93
0.11	0.05	0.03	1.76
1.03	1.52	0.92	1.65
2.00	2.95	1.85	1.60
4.12	1.82	1.22	1.49

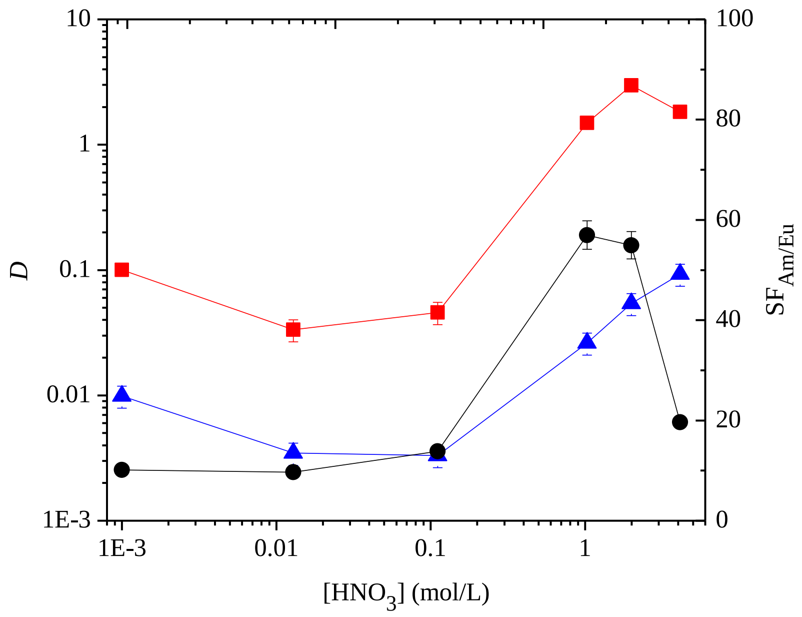

Figure 11. Extraction of Am(III) and Eu(III) by ligand **8** (0.01 M) + *N*,*N*'-dimethyl-*N*,*N*'-dioctyl-2-hexyloxyethyl malonamide **11** (0.25 M) into 1-octanol as a function of the initial nitric acid concentration of the aqueous phase (*D* = distribution ratio, SF = separation factor, ■ = D_{Am}, ▲ = D_{Eu}, ● = $SF_{Am/Eu}$, contact time: 60 min, temperature: 22 °C ± 1 °C).

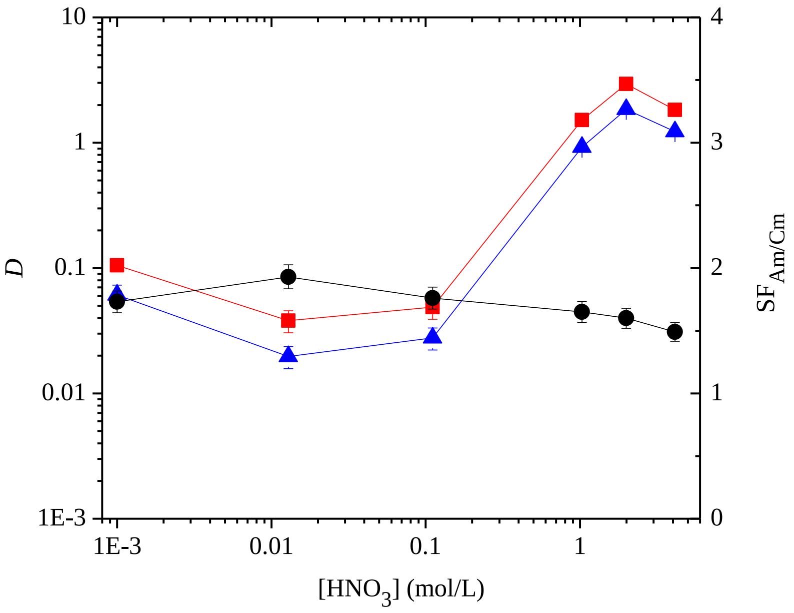

Figure 12. Extraction of Am(III) and Cm(III) by ligand **8** (0.01 M) + *N*,*N*'-dimethyl-*N*,*N*'-dioctyl-2-hexyloxyethyl malonamide **11** (0.25 M) into 1-octanol as a function of the initial nitric acid concentration of the aqueous phase (D = distribution ratio, SF = separation factor, ■ = D_{Am}, ▲ = D_{Cm}, ● = $SF_{Am/Cm}$, contact time: 60 min, temperature: 22 °C ± 1 °C).

Table 13. Extraction of Y(III) and all the trivalent lanthanides (except Pm) by ligand **8** + *N*,*N*'-dimethyl-*N*,*N*'-dioctyl-2-hexyloxyethyl malonamide **11** (0.25 M) as a function of the initial nitric acid concentration of the aqueous phase. Results are from ICP-MS (*D* = distribution ratio, SF = separation factor, contact time: 60 min, temperature: 22 °C ± 1 °C).

**[HNO_3] (mol/L)**		0.001	0.01	0.11	1.03	2.00	4.12
**Element**	**Atomic Number**	*D*	*D*	*D*	*D*	*D*	*D*
Y	39	0.001	0.001	0.001	0.006	0.024	0.043
La	57	0.001	0.000	0.001	0.006	0.021	0.048
Ce	58	0.001	0.001	0.016	0.018	0.055	0.084
Pr	59	0.000	0.000	0.001	0.009	0.033	0.075
Nd	60	0.001	0.001	≤0.001	0.011	0.036	0.074
Sm	62	0.001	0.001	0.001	0.017	0.051	0.087
Eu	63	0.001	0.001	0.001	0.020	0.060	0.091
Gd	64	0.001	0.001	0.001	0.019	0.058	0.082
Tb	65	0.001	0.001	0.001	0.044	0.146	0.154
Dy	66	0.002	0.002	0.002	0.079	0.272	0.207
Ho	67	0.003	0.003	0.003	0.115	0.335	0.193
Er	68	0.007	0.004	0.004	0.130	0.318	0.202
Tm	69	0.021	0.009	0.004	0.104	0.259	0.176
Yb	70	0.070	0.025	0.006	0.085	0.211	0.188
Lu	71	0.195	0.062	0.010	0.058	0.161	0.188

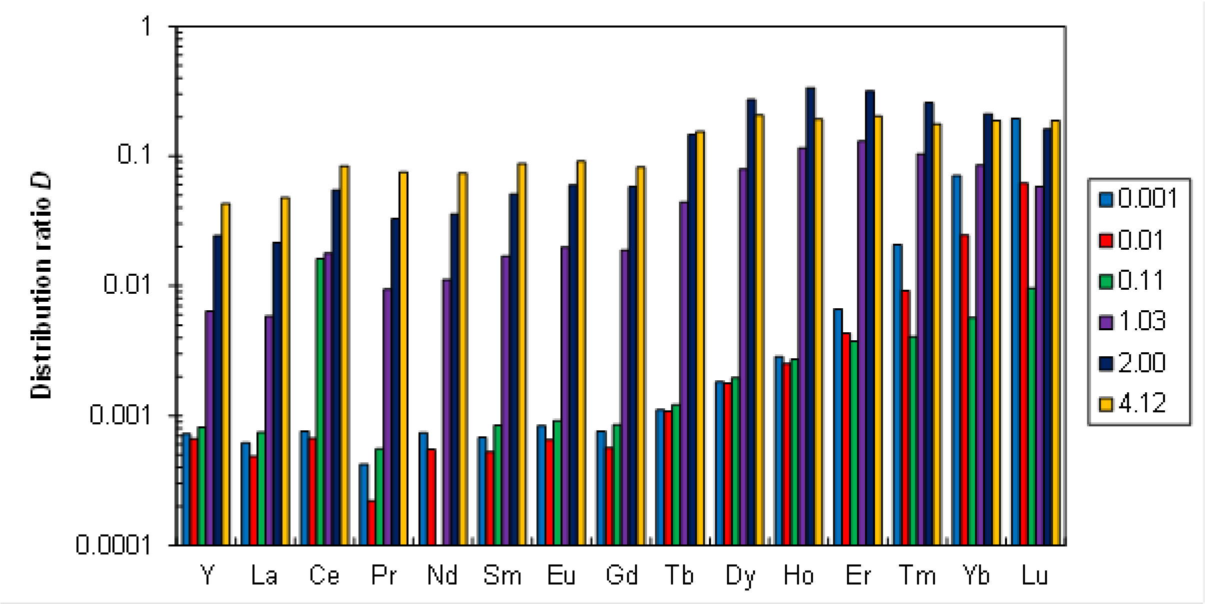

Figure 13. Extraction of Y(III) and all the trivalent lanthanides (except Pm) by ligand **8** + *N*,*N*'-dimethyl-*N*,*N*'-dioctyl-2-hexyloxyethyl malonamide **11** (0.25 M) as a function of the initial nitric acid concentration (in mol dm^{-3}) of the aqueous phase. Results are from ICP-MS (*D* = distribution ratio, SF = separation factor, contact time: 60 min, temperature: 22 ^{o}C ± 1 ^{o}C).

4.3 Extraction Studies for Ligand 10

Table 14. Extraction of Am(III) and Eu(III) by ligand **10** as a function of the initial nitric acid concentration of the aqueous phase. Results are from gamma spectrometry (*D* = distribution ratio, SF = separation factor, contact time: 60 min, temperature: 22 °C ± 1 °C).

**[HNO_3] initial (mol/L)**	D_{Am}	D_{Eu}	$SF_{Am/Eu}$
0.01	3.427	0.032	106.81
0.11	19.250	0.100	191.78
0.30	60.596	0.279	217.28
0.70	90.925	0.434	209.65
1.03	111.813	0.472	236.89
3.11	109.306	0.551	198.32

Table 15. Extraction of Am(III) and Cm(III) by ligand **10** as a function of the initial nitric acid concentration of the aqueous phase. Results are from alpha spectrometry (*D* = distribution ratio, SF = separation factor, contact time: 60 min, temperature: 22 °C ± 1 °C).

**[HNO_3] initial (mol/L)**	D_{Am}	D_{Cm}	$SF_{Am/Cm}$
0.01	3.20	1.65	1.94
0.11	16.80	8.26	2.03
0.30	63.22	27.94	2.26
0.70	84.56	39.94	2.12
1.03	94.39	50.39	1.87
3.11	93.00	51.45	1.81

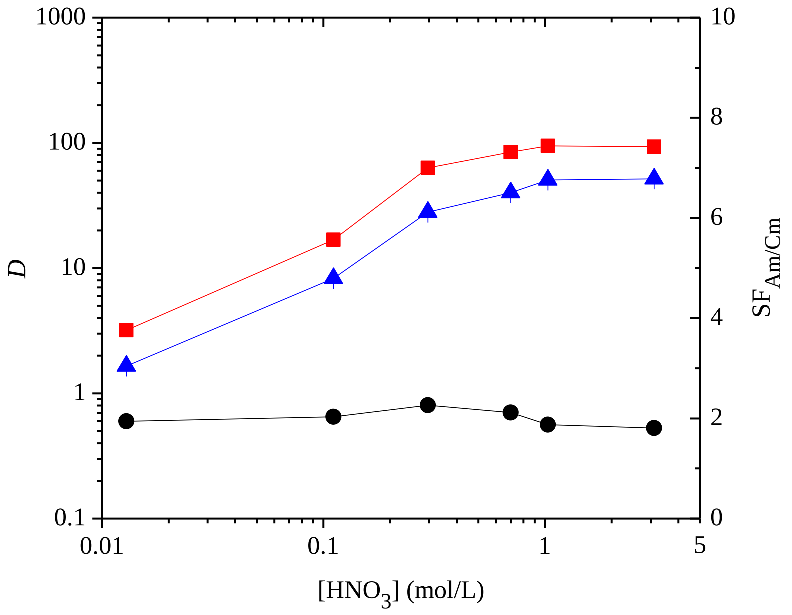

Figure 14. Extraction of Am(III) and Cm(III) by ligand **10** (0.01 M) into 1-octanol as a function of the initial nitric acid concentration of the aqueous phase (*D* = distribution ratio, SF = separation factor, ■ = D_{Am}, ▲ = D_{Cm}, ● = $SF_{Am/Cm}$, contact time: 60 min, temperature: 22 °C ± 1 °C).

Table 16. Extraction of Am(III) and Eu(III) from 1.03 M nitric acid by ligand **10** as a function of contact time. Results are from gamma spectrometry (*D* = distribution ratio, SF = separation factor, temperature: 22 ^{o}C ± 1 ^{o}C).

**Contact time (min)**	D_{Am}	D_{Eu}	$SF_{Am/Eu}$
5	77.047	0.245	313.94
10	89.666	0.355	252.51
20	95.355	0.460	207.29
30	97.330	0.467	208.43
45	95.239	0.484	196.96
60	94.308	0.487	193.74

Table 17. Extraction of Am(III) and Cm(III) from 1.03 M nitric acid by ligand **10** as a function of contact time. Results are from alpha spectrometry (*D* = distribution ratio, SF = separation factor, temperature: 22 ^{o}C ± 1 ^{o}C).

**Contact time (min)**	D_{Am}	D_{Cm}	$SF_{Am/Cm}$
5	51.21	23.45	2.18
10	50.70	33.65	1.51
20	101.95	45.61	2.24
30	76.89	39.73	1.94
45	88.15	38.53	2.29
60	78.11	42.71	1.83

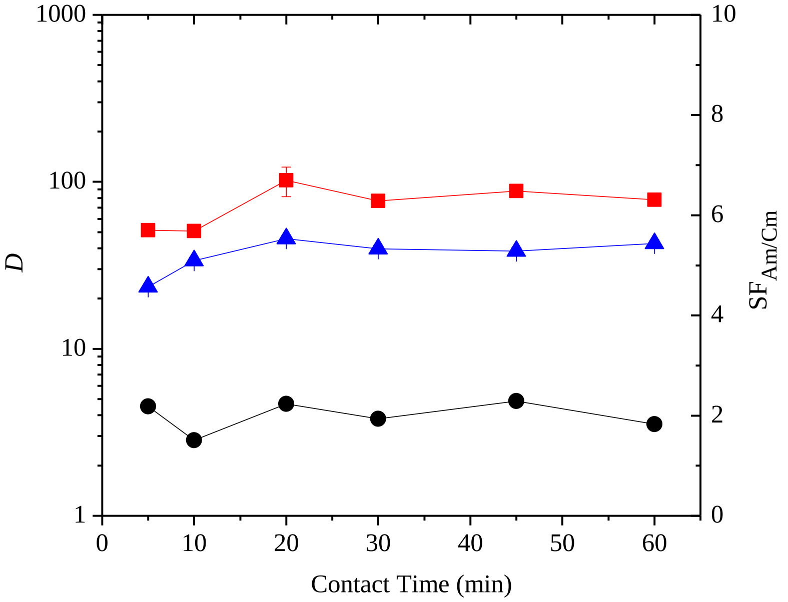

Figure 15. Extraction of Am(III) and Cm(III) from 1.03 M nitric acid by ligand **10** (0.01 M) into 1-octanol as a function of contact time (*D* = distribution ratio, SF = separation factor, ■ = D_{Am}, ▲ = D_{Cm}, ● = $SF_{Am/Cm}$, temperature: 22 ^{o}C ± 1 ^{o}C).

Table 18. Extraction of Y(III) and all the trivalent lanthanides (except Pm) by ligand **10** as a function of the initial nitric acid concentration of the aqueous phase. Results are from ICP-MS (*D* = distribution ratio, SF = separation factor, contact time: 60 min, temperature: 22 °C ± 1 °C).

**[HNO$_3$] (mol/L)**		0.01	0.11	0.30	0.70	1.03	3.11
**Element**	**Atomic Number**	*D*	*D*	*D*	*D*	*D*	*D*
Y	39	0.011	0.010	0.027	0.051	0.064	0.124
La	57	0.016	0.009	0.010	0.010	0.009	0.007
Ce	58	0.032	0.031	0.043	0.052	0.049	0.035
Pr	59	0.040	0.053	0.091	0.123	0.120	0.095
Nd	60	0.044	0.075	0.155	0.221	0.216	0.195
Sm	62	0.052	0.122	0.309	0.472	0.477	0.520
Eu	63	0.048	0.123	0.326	0.515	0.539	0.658
Gd	64	0.035	0.085	0.240	0.400	0.428	0.605
Tb	65	0.056	0.159	0.511	0.933	1.052	1.810
Dy	66	0.069	0.220	0.765	1.483	1.735	3.254
Ho	67	0.080	0.270	0.958	1.841	2.302	3.566
Er	68	0.114	0.314	1.017	1.891	2.365	2.764
Tm	69	0.208	0.327	0.868	1.538	1.844	1.865
Yb	70	0.447	0.370	0.738	1.158	1.292	1.169
Lu	71	0.876	0.444	0.641	0.832	0.861	0.745

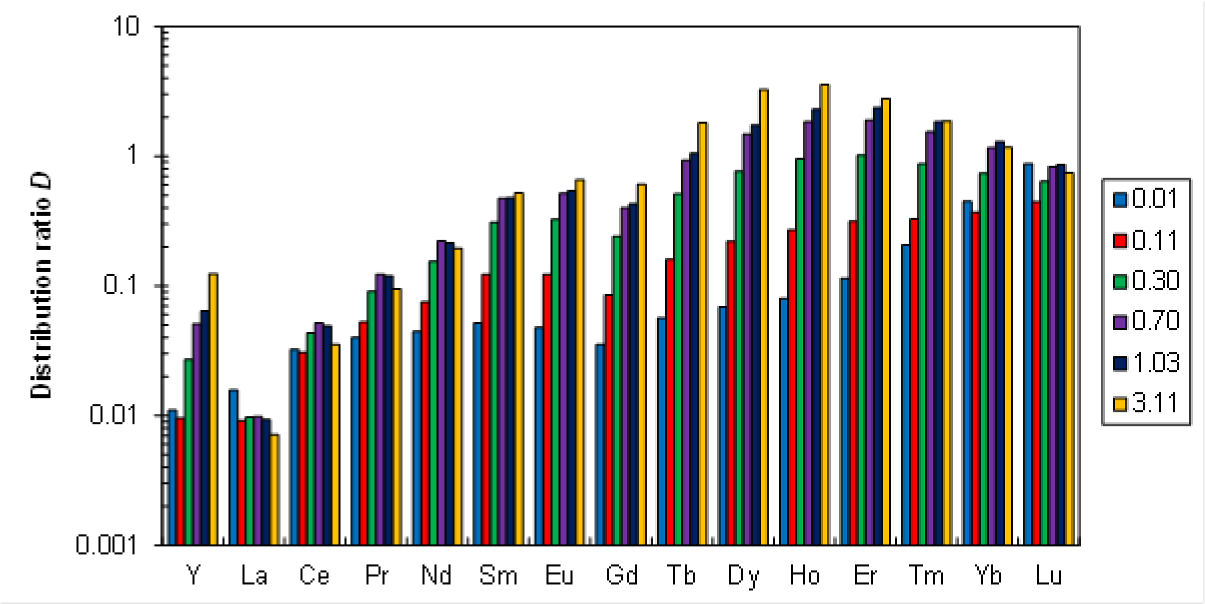

Figure 16. Extraction of Y(III) and all the trivalent lanthanides (except Pm) by ligand **10** as a function of the initial nitric acid concentration (in mol dm^{-3}) of the aqueous phase. Results are from ICP-MS (*D* = distribution ratio, SF = separation factor, contact time: 60 min, temperature: 22 ^{o}C ± 1 ^{o}C).

5: NMR Titrations with Metal Salts

5.1 NMR Titrations of Ligand 6 with Metal Salts

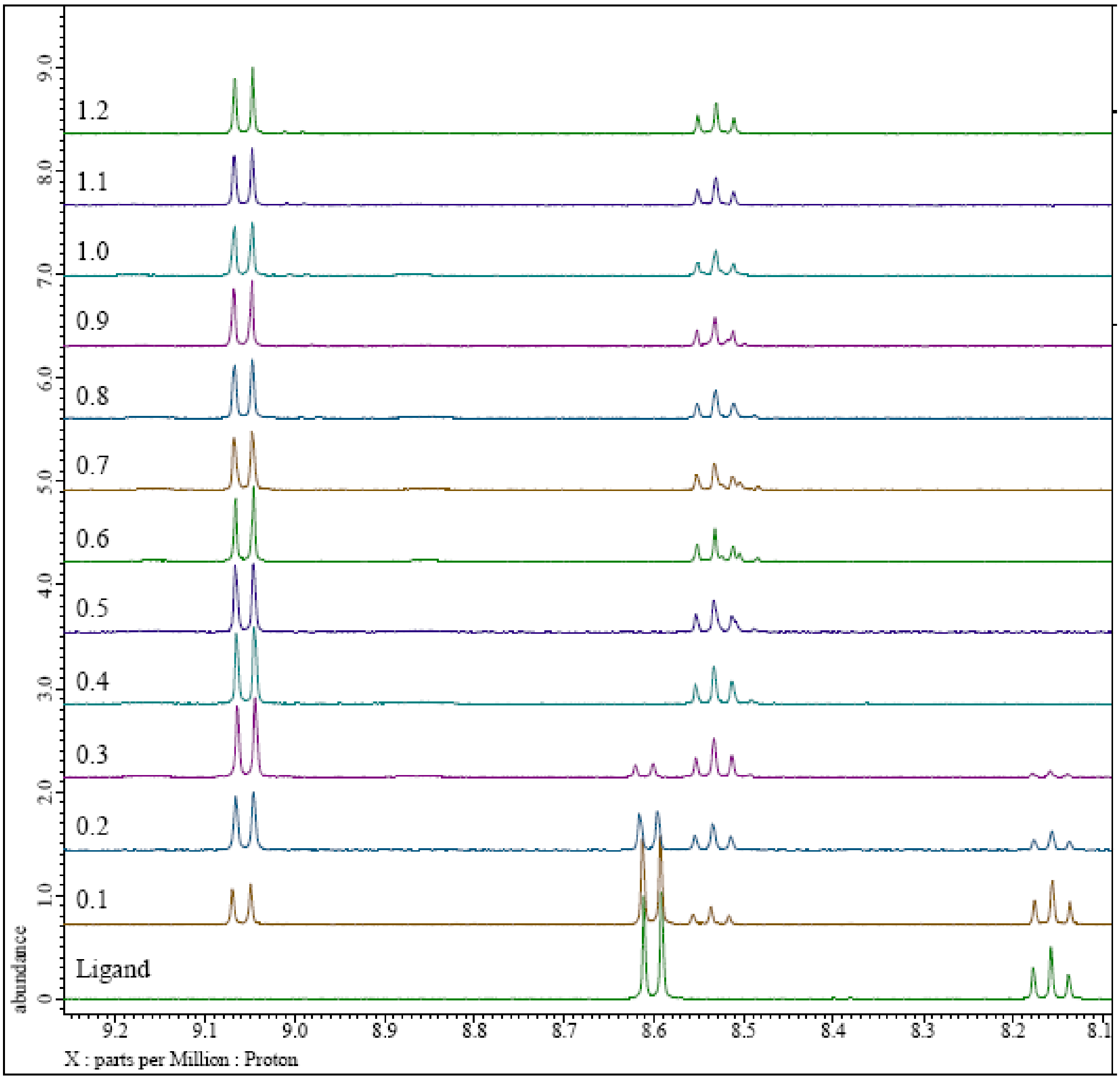

Figure 17. Aromatic region of the stack plot for the ^1H NMR titration of ligand **6** with $Y(NO_3)_3$ in CD_3CN. Bottom spectrum = free ligand. Each preceding spectrum corresponds to the addition of 0.1 equivalents of metal salt solution.

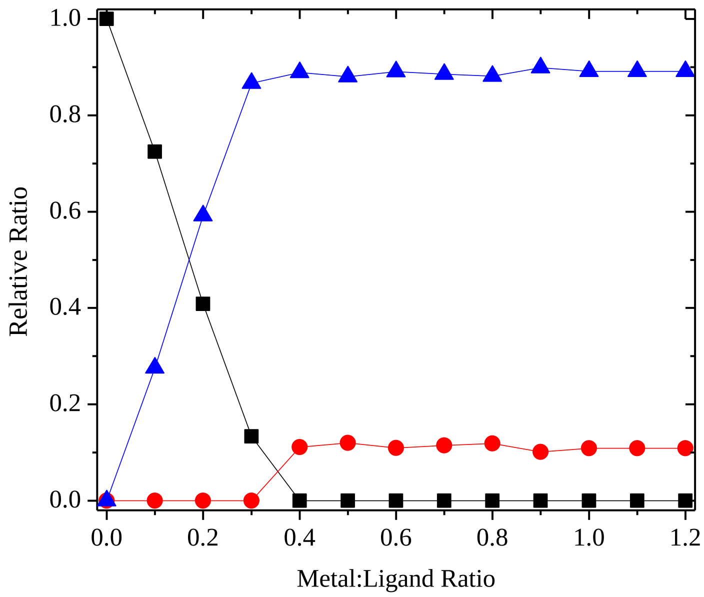

Figure 18. ^{1}H NMR titration of ligand **6** with $Y(NO_3)_3$ in CD_3CN (■ = free ligand, ● = 1:2 complex, ▲ = 1:3 complex).

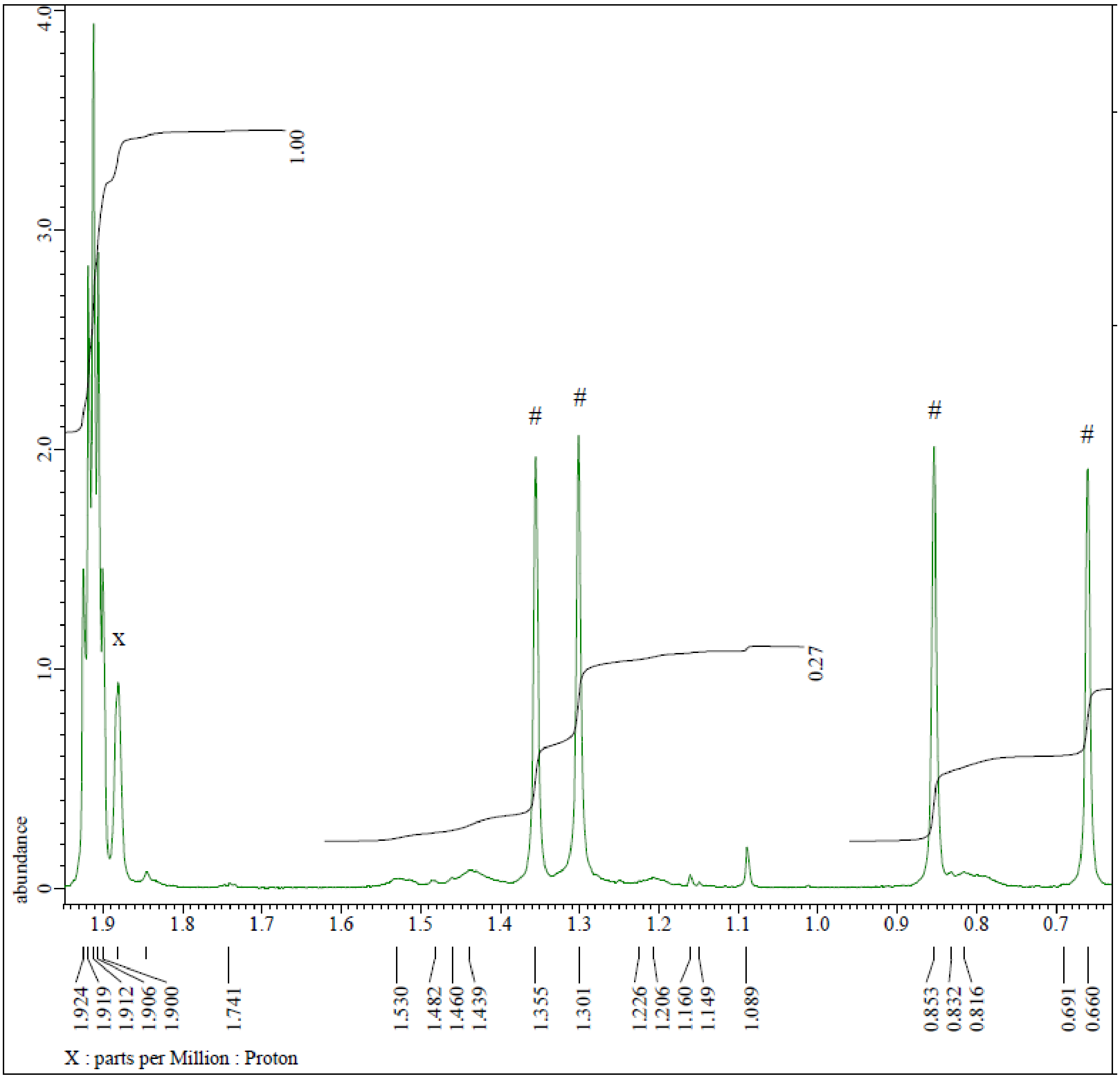

Figure 19. Enlargement of the aliphatic region of the ^1H NMR spectrum of a mixture of ligand **6** and $Y(NO_3)_3$ (0.5 equivalents) in CD_3CN (Assignments: # = diastereotopic methyl protons of the chiral 1:3 complex cation $[Y(\mathbf{6})_3]^{3+}$, x = methylene protons of the chiral 1:3 complex cation $[Y(\mathbf{6})_3]^{3+}$). Peak at 1.91 ppm is due to solvent.

5.2 NMR Titrations of Ligand 8 with Metal Salts

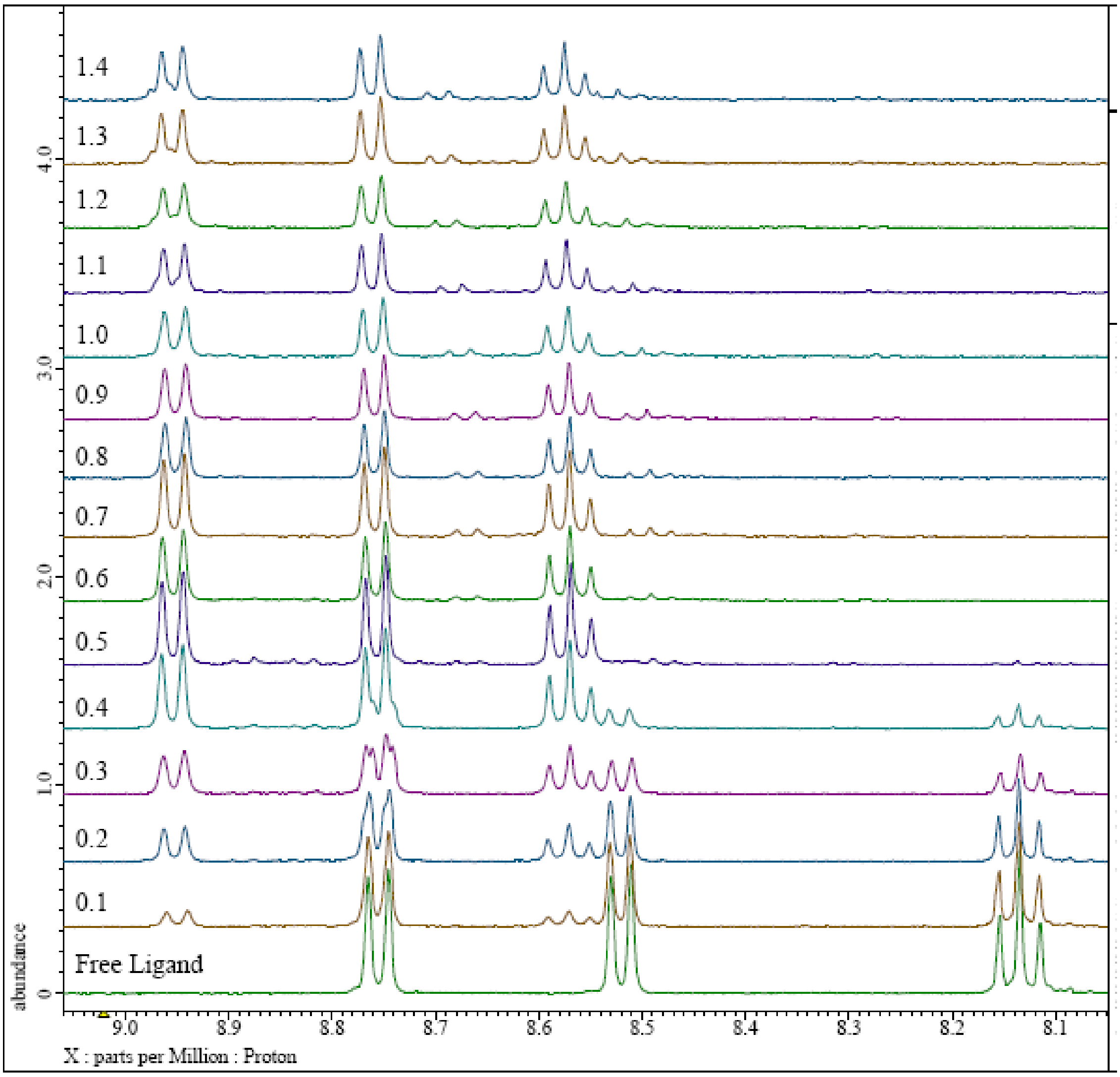

Figure 20. Aromatic region of the stack plot for the ^{1}H NMR titration of ligand **8** with $Y(NO_3)_3$ in CD_3CN. Bottom spectrum = free ligand. Each preceding spectrum corresponds to the addition of 0.1 equivalents of metal salt solution.

5.3 NMR Titrations of Ligand 10 with Metal Salts

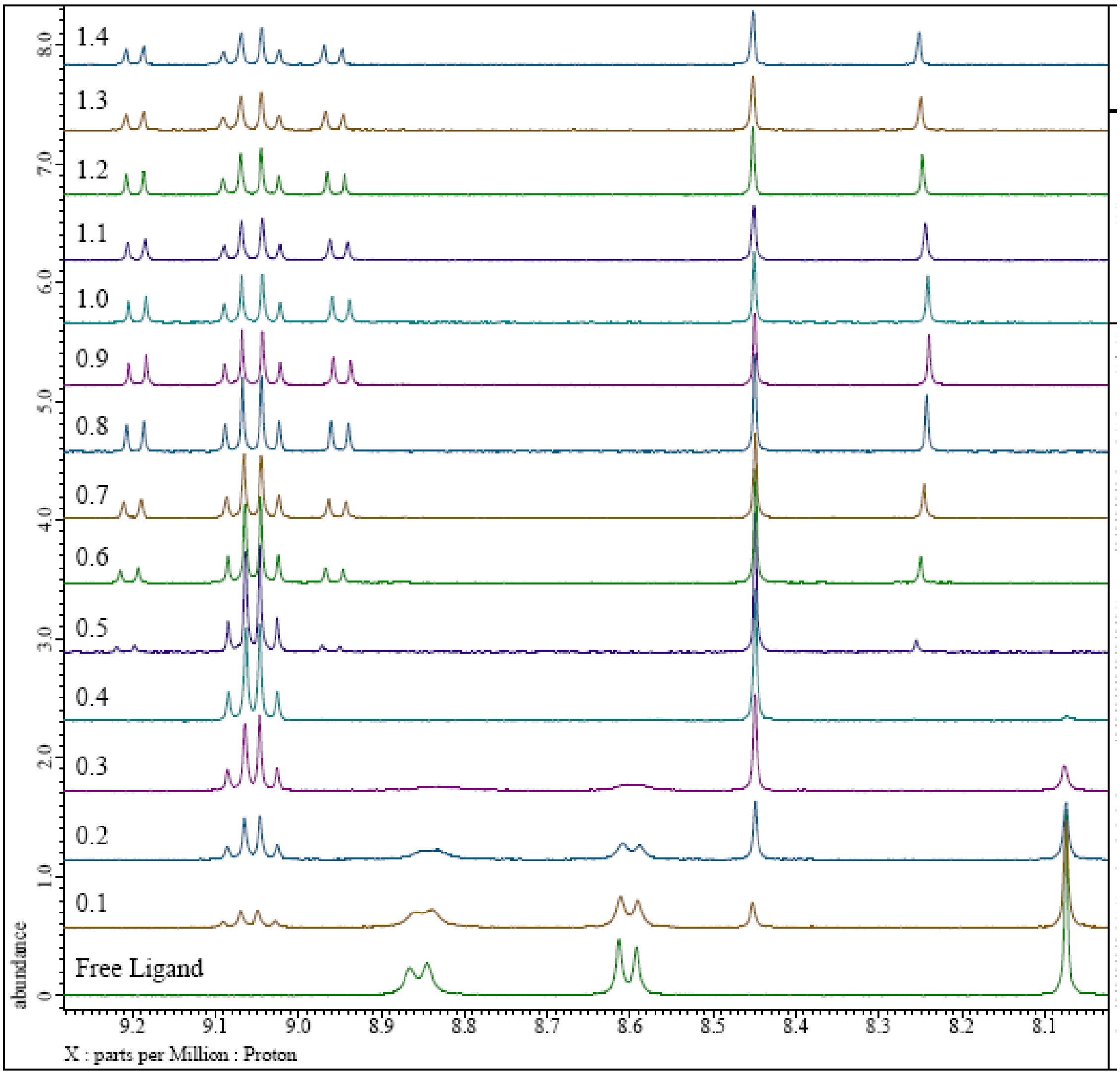

Figure 21. Aromatic region of the stack plot for the ^{1}H NMR titration of ligand **10** with $La(NO_3)_3$ in CD_3CN. Bottom spectrum = free ligand. Each preceding spectrum corresponds to the addition of 0.1 equivalents of metal salt solution.

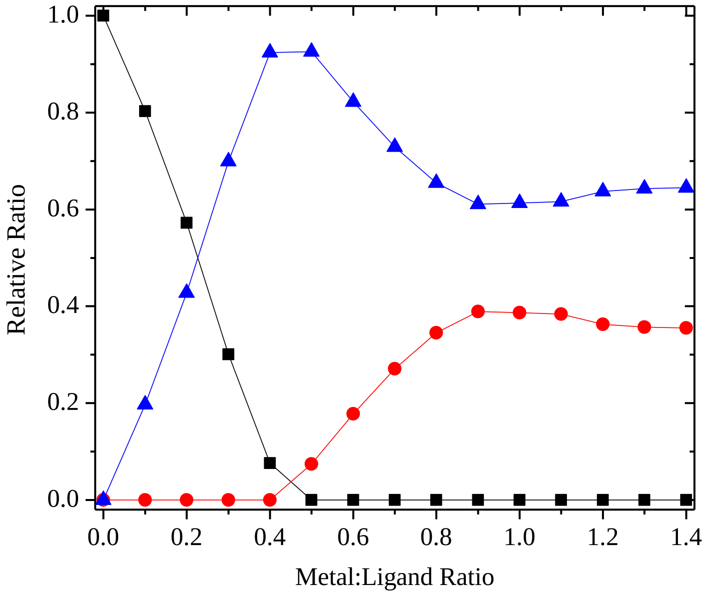

Figure 22. ^{1}H NMR titration of ligand **10** with $La(NO_3)_3$ in CD_3CN (Key: ■ = free ligand, ● = 1:1 complex, ▲ = 1:2 complex).

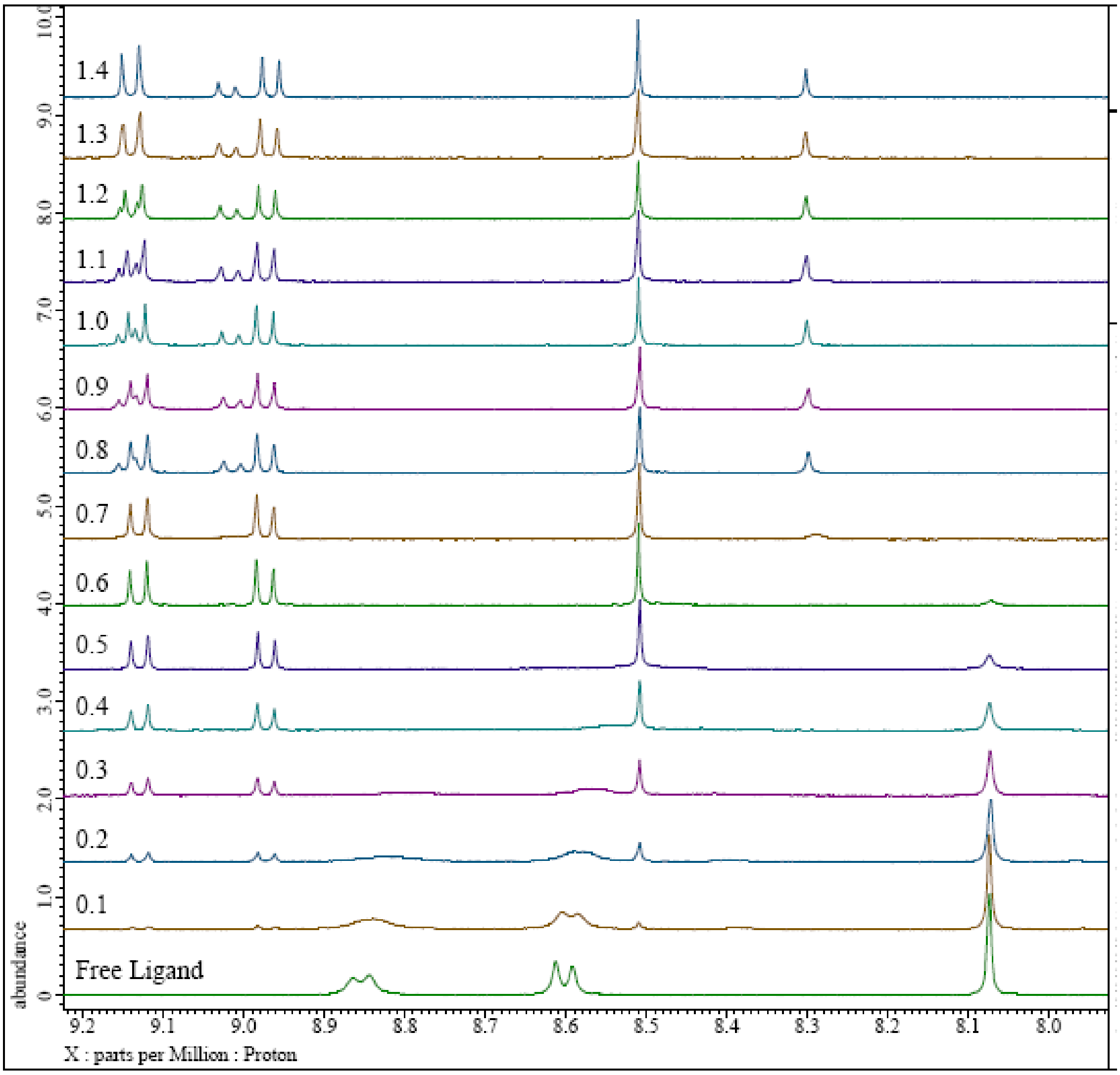

Figure 23. Aromatic region of the stack plot for the ^{1}H NMR titration of ligand **10** with $Lu(NO_3)_3$ in CD_3CN. Bottom spectrum = free ligand. Each preceding spectrum corresponds to the addition of 0.1 equivalents of metal salt solution.

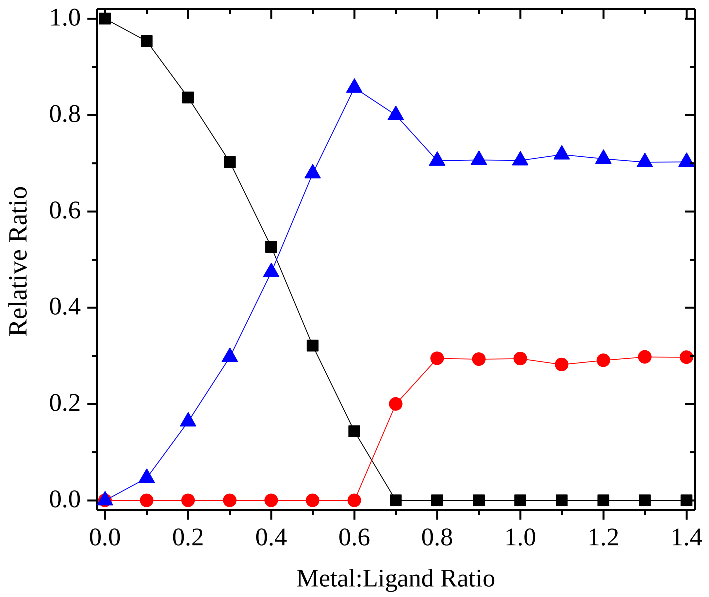

Figure 24. ^{1}H NMR titration of ligand **10** with $Lu(NO_3)_3$ in CD_3CN (■ = free ligand, ● = 1:1 complex, ▲ = 1:2 complex).

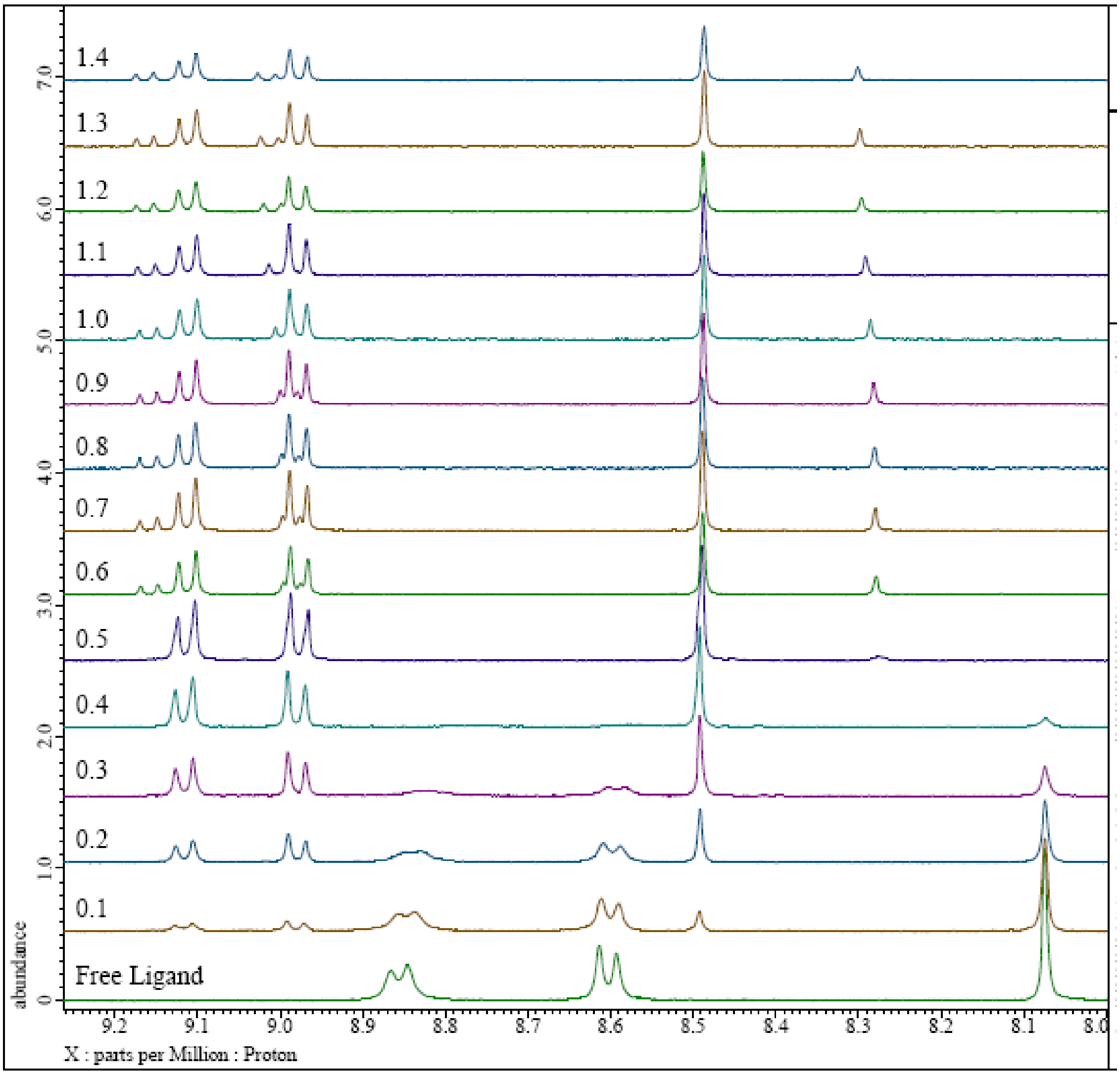

Figure 25. Aromatic region of the stack plot for the ^{1}H NMR titration of ligand **10** with $Y(NO_3)_3$ in CD_3CN. Bottom spectrum = free ligand. Each preceding spectrum corresponds to the addition of 0.1 equivalents of metal salt solution.

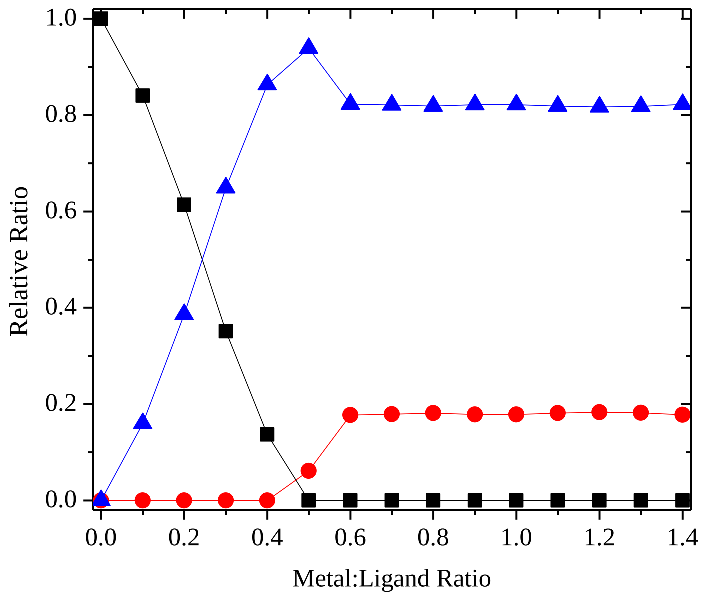

Figure 26. ^{1}H NMR titration of ligand **10** with $Y(NO_3)_3$ in CD_3CN (■ = free ligand, ● = 1:1 complex, ▲ = 1:2 complex).

5.4 NMR Competition Experiments

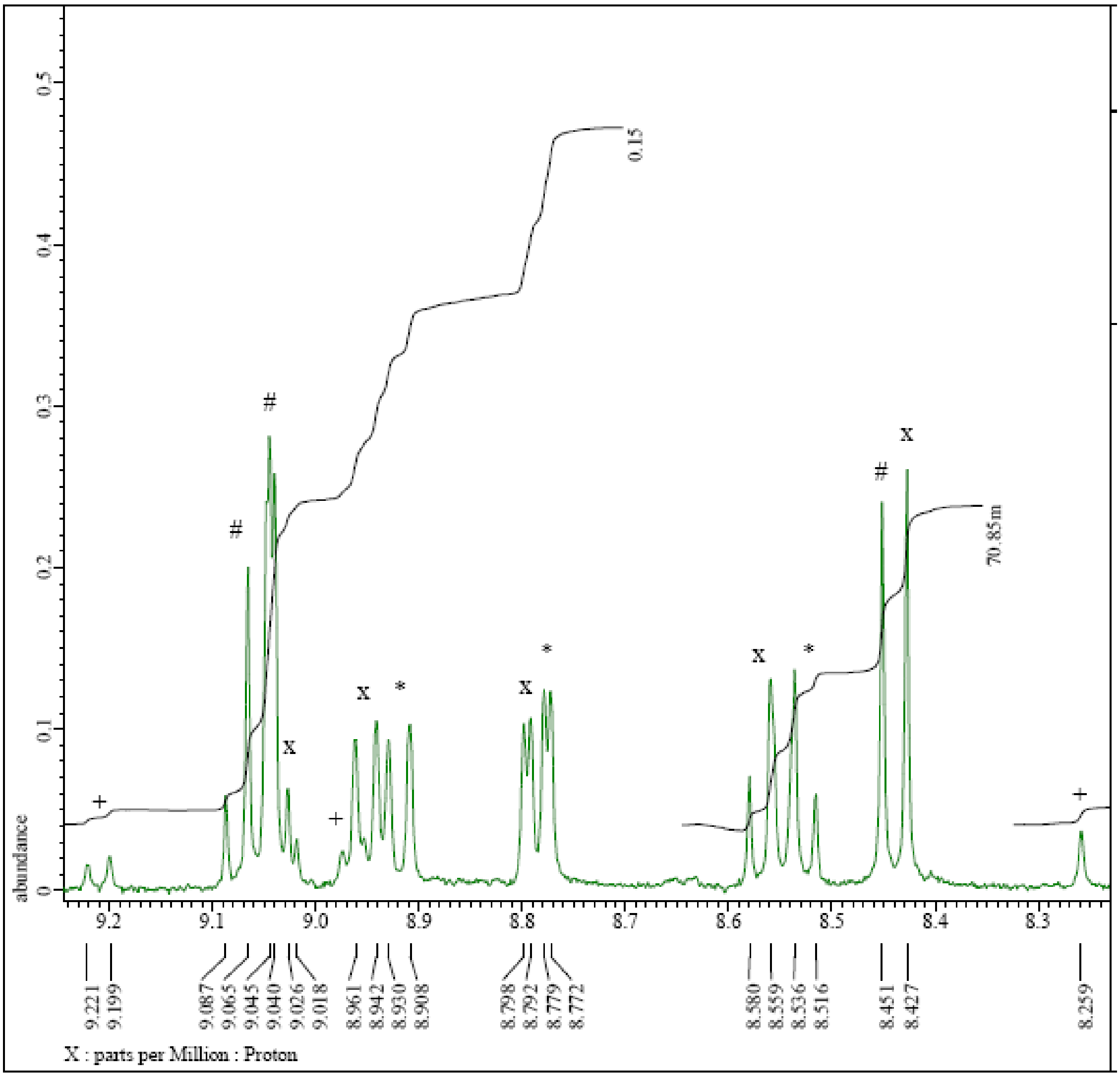

Figure 27. Aromatic region of the ^{1}H NMR spectrum of a 1:1:1 mixture of ligand **8**, ligand **10** and $La(NO_3)_3$ in CD_3CN (Assignments: * = 1:2 bis-complex of **8**, # = 1:2 bis-complex of **10**, x = heteroleptic 1:2 bis-complex, + = 1:1 complex of **10**).

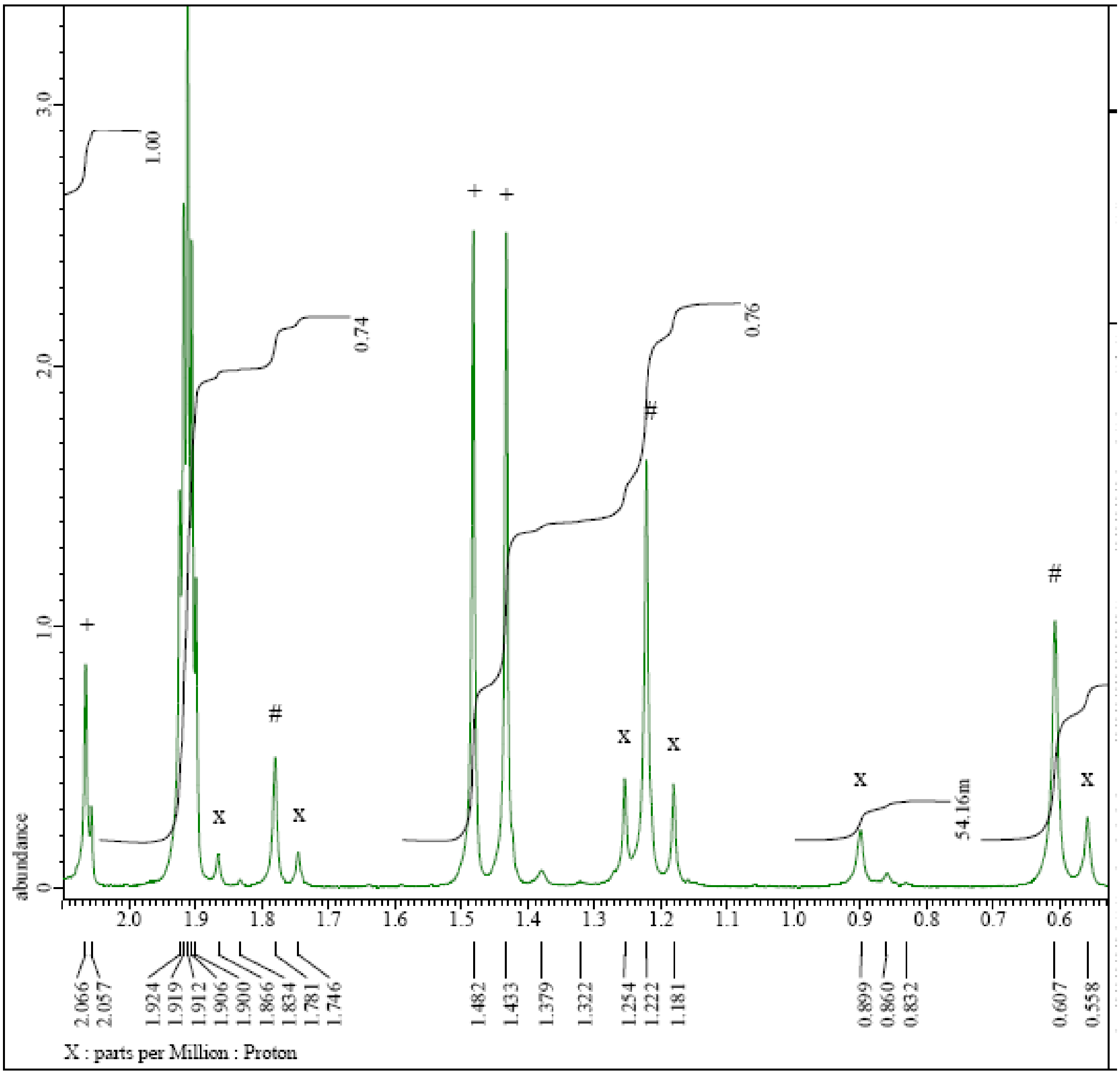

Figure 28. Aliphatic region of the ^{1}H NMR spectrum of the 1:2 bis-complex of ligand **8** with $La(NO_3)_3$ in CD_3CN after ligand **10** (1 equivalent) had been added (Assignments: # = 1:2 bis-complex of **10**, x = heteroleptic 1:2 bis-complex, + = free uncomplexed ligand **8**). Peak at 1.91 ppm is due to solvent.

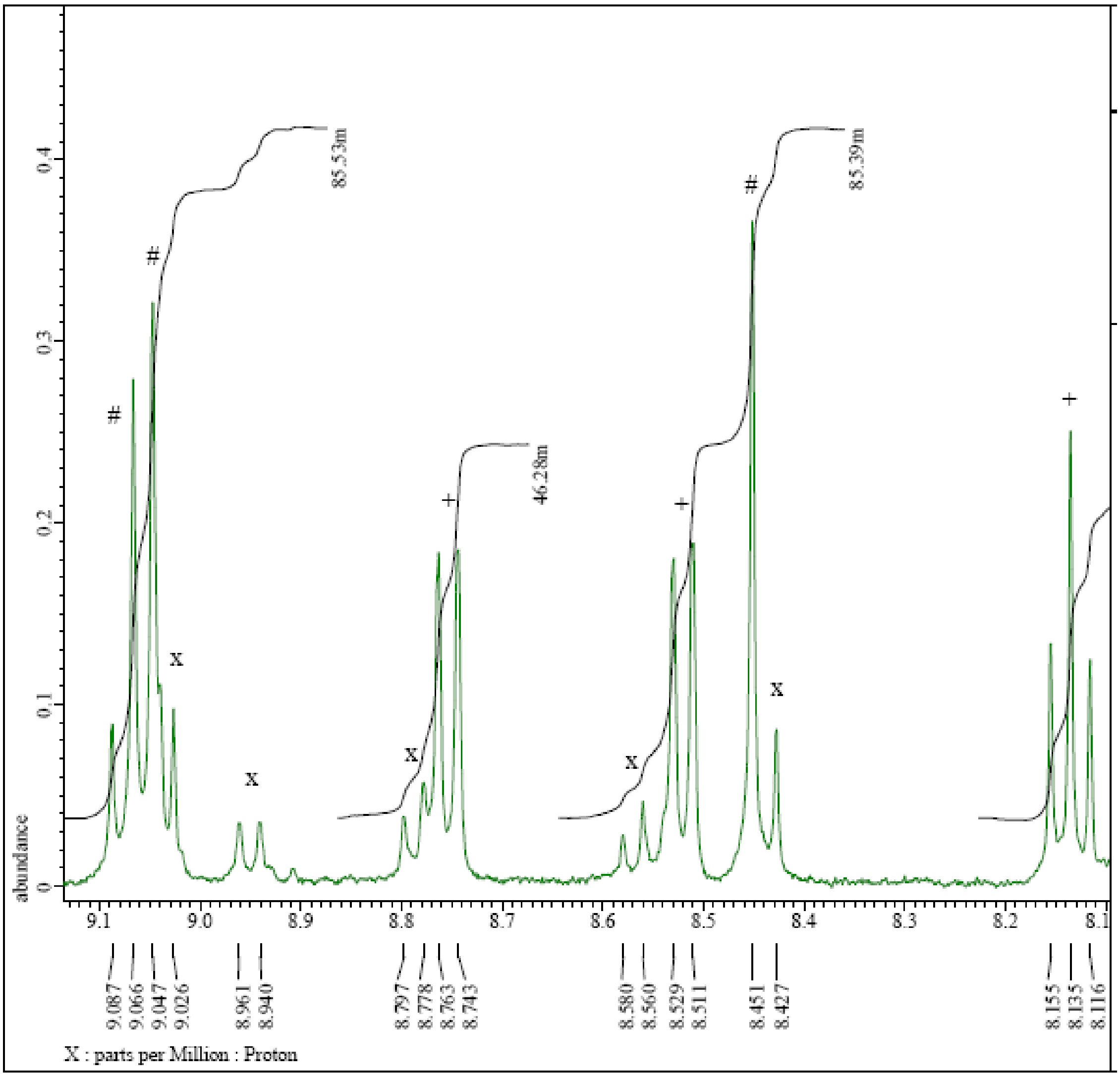

Figure 29. Aromatic region of the ^{1}H NMR spectrum of the 1:2 bis-complex of ligand **8** with $La(NO_3)_3$ in CD_3CN after ligand **10** (1 equivalent) had been added (Assignments: # = 1:2 bis-complex of **10**, x = heteroleptic 1:2 bis-complex, + = free uncomplexed ligand **8**).

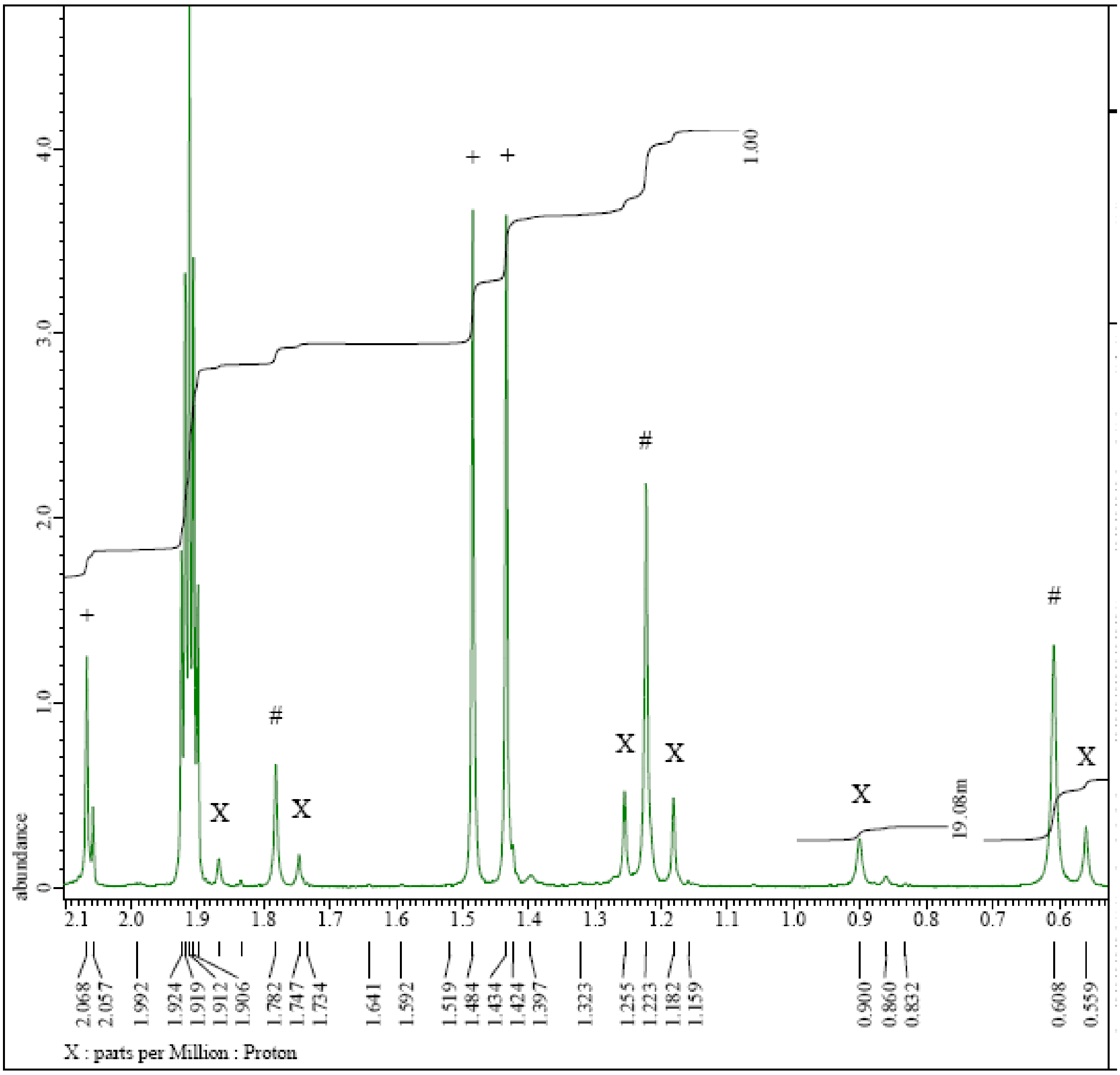

Figure 30. Aliphatic region of the ^{1}H NMR spectrum of the 1:2 bis-complex of ligand **10** with $La(NO_3)_3$ in CD_3CN after ligand **8** (1 equivalent) had been added (Assignments: # = 1:2 bis-complex of **10**, x = heteroleptic 1:2 bis-complex, + = free uncomplexed ligand **8**). Peak at 1.91 ppm is due to solvent.

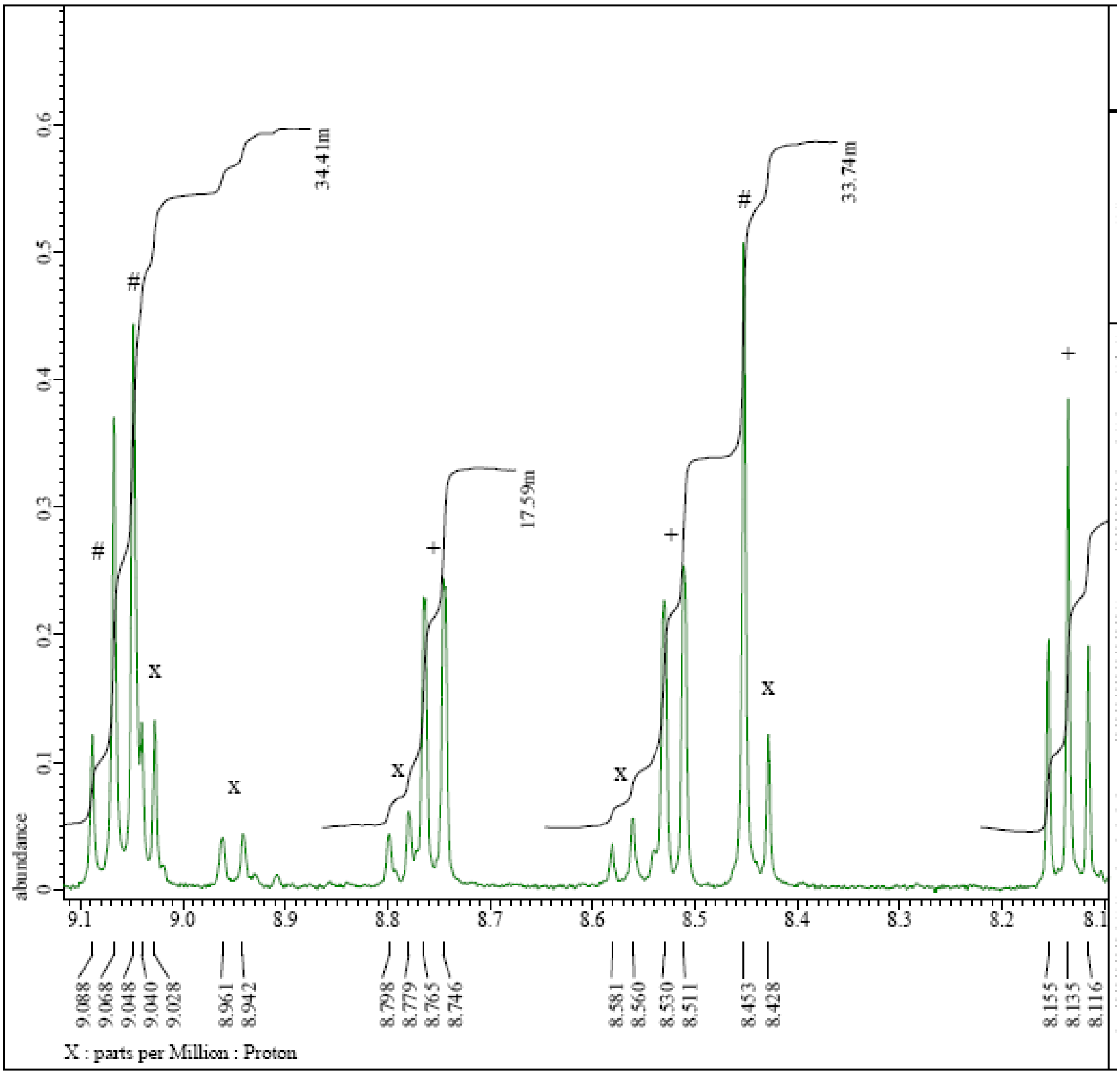

Figure 31. Aromatic region of the ^{1}H NMR spectrum of the 1:2 bis-complex of ligand **10** with $La(NO_3)_3$ in CD_3CN after ligand **8** (1 equivalent) had been added (Assignments: # = 1:2 bis-complex of **10**, x = heteroleptic 1:2 bis-complex, + = free uncomplexed ligand **8**).

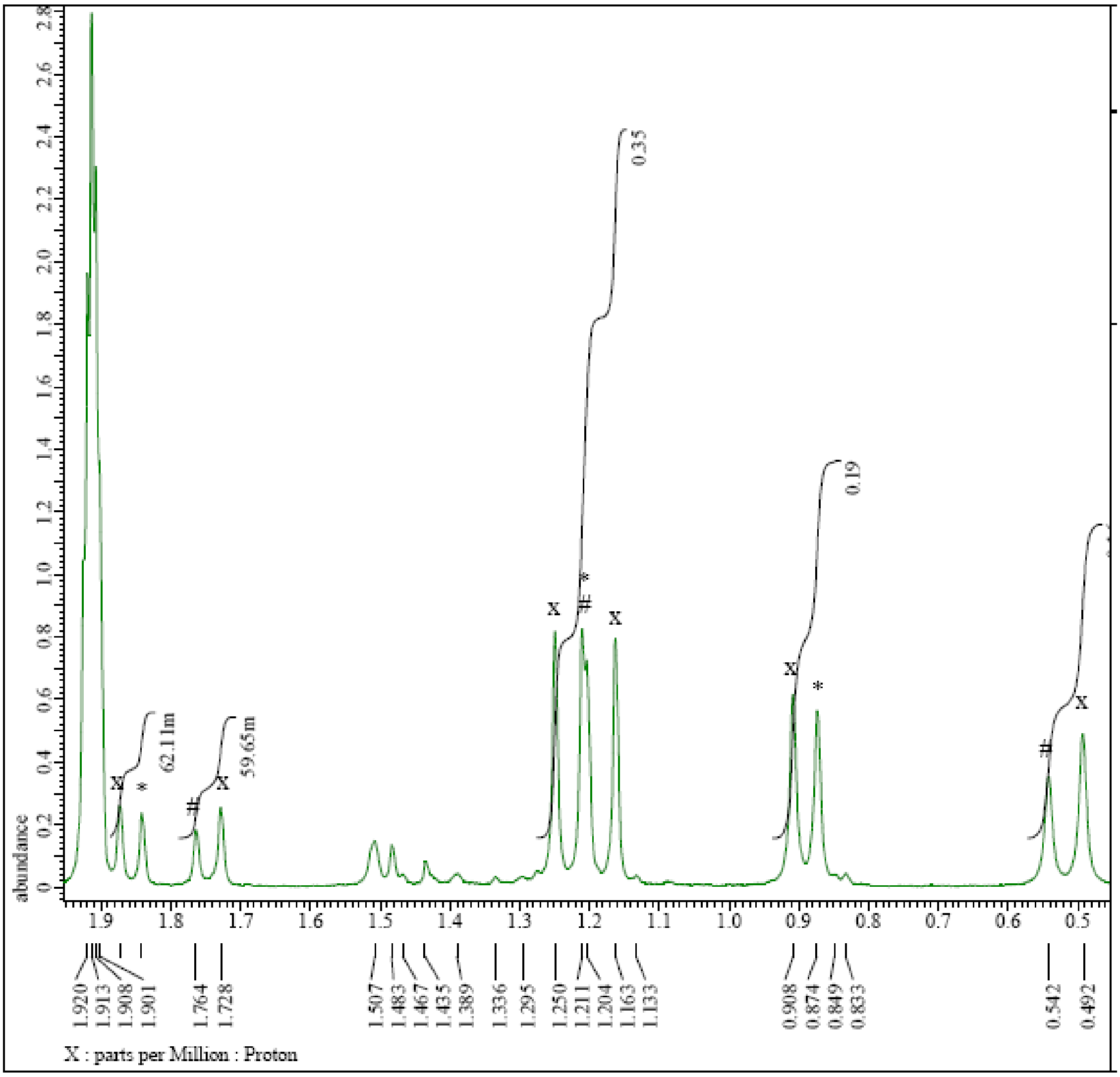

Figure 32. Aliphatic region of the ^{1}H NMR spectrum of a 1:1:1 mixture of ligand **8**, ligand **10** and $Y(NO_3)_3$ in CD_3CN (Assignments: * = 1:2 bis-complex of **8**, # = 1:2 bis-complex of **10**, x = heteroleptic 1:2 bis-complex). Peak at 1.91 ppm is due to solvent.

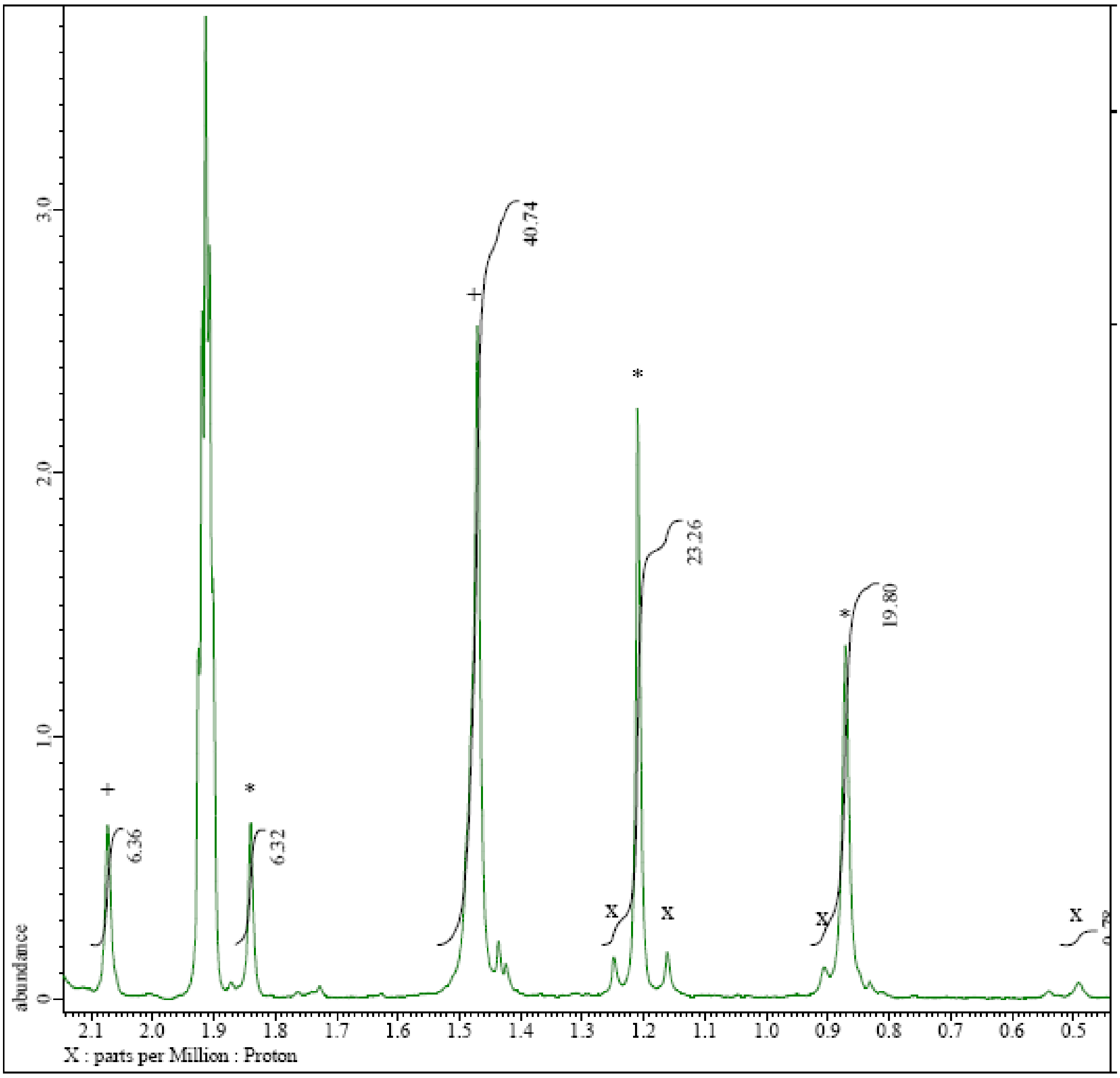

Figure 33. Aliphatic region of the ^{1}H NMR spectrum of the 1:2 bis-complex of ligand **8** with $Y(NO_3)_3$ in CD_3CN after ligand **10** (1 equivalent) had been added (Assignments: * = 1:2 bis-complex of **8**, + = free uncomplexed ligand **10**, x = heteroleptic 1:2 bis-complex). Peak at 1.91 ppm is due to solvent.

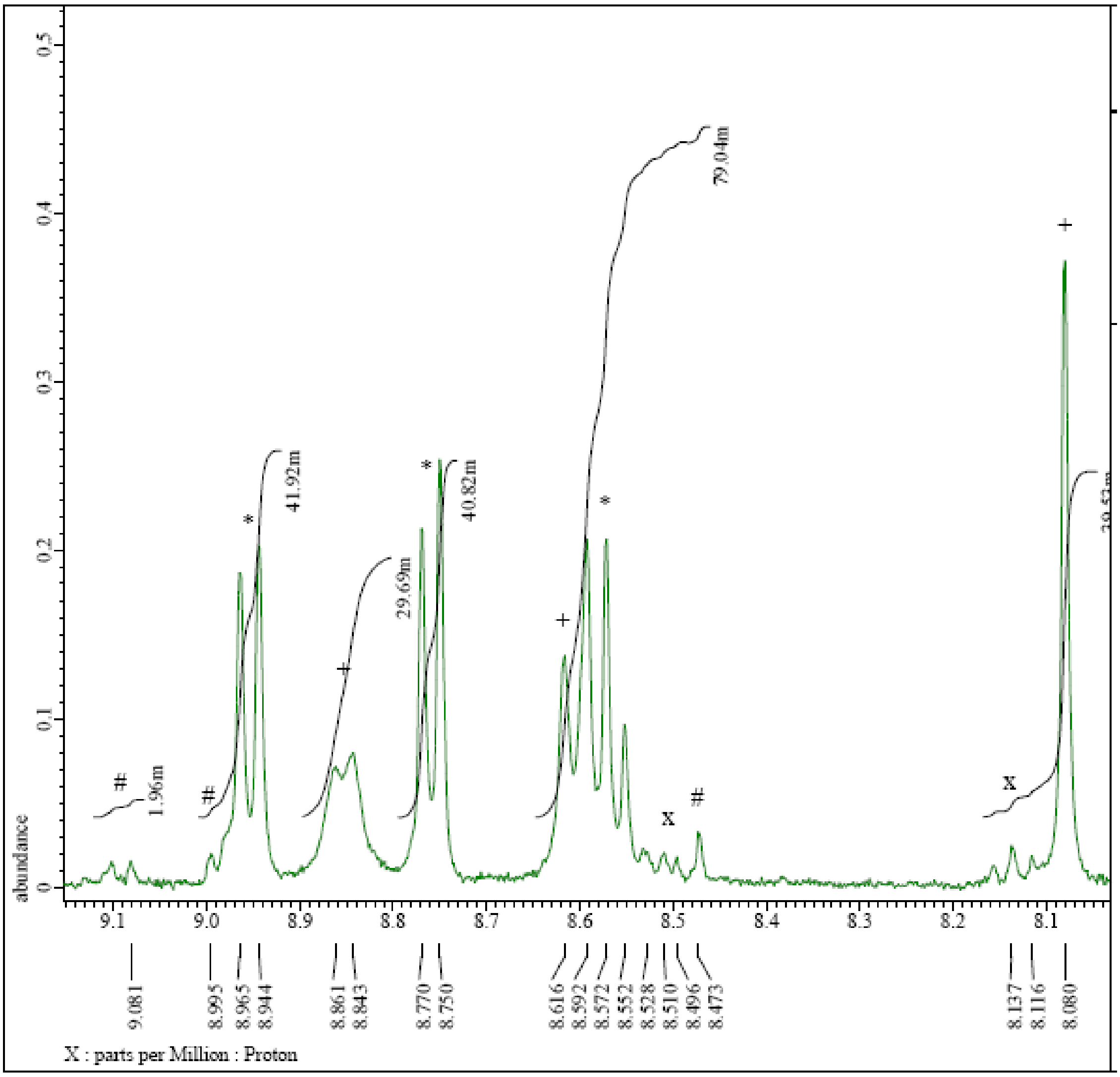

Figure 34. Aromatic region of the ^{1}H NMR spectrum of the 1:2 bis-complex of ligand **8** with $Y(NO_3)_3$ in CD_3CN after ligand **10** (1 equivalent) had been added (Assignments: * = 1:2 bis-complex of **8**, + = free uncomplexed ligand **10**, # = 1:2 bis-complex of **10**, x = free uncomplexed ligand **8**).

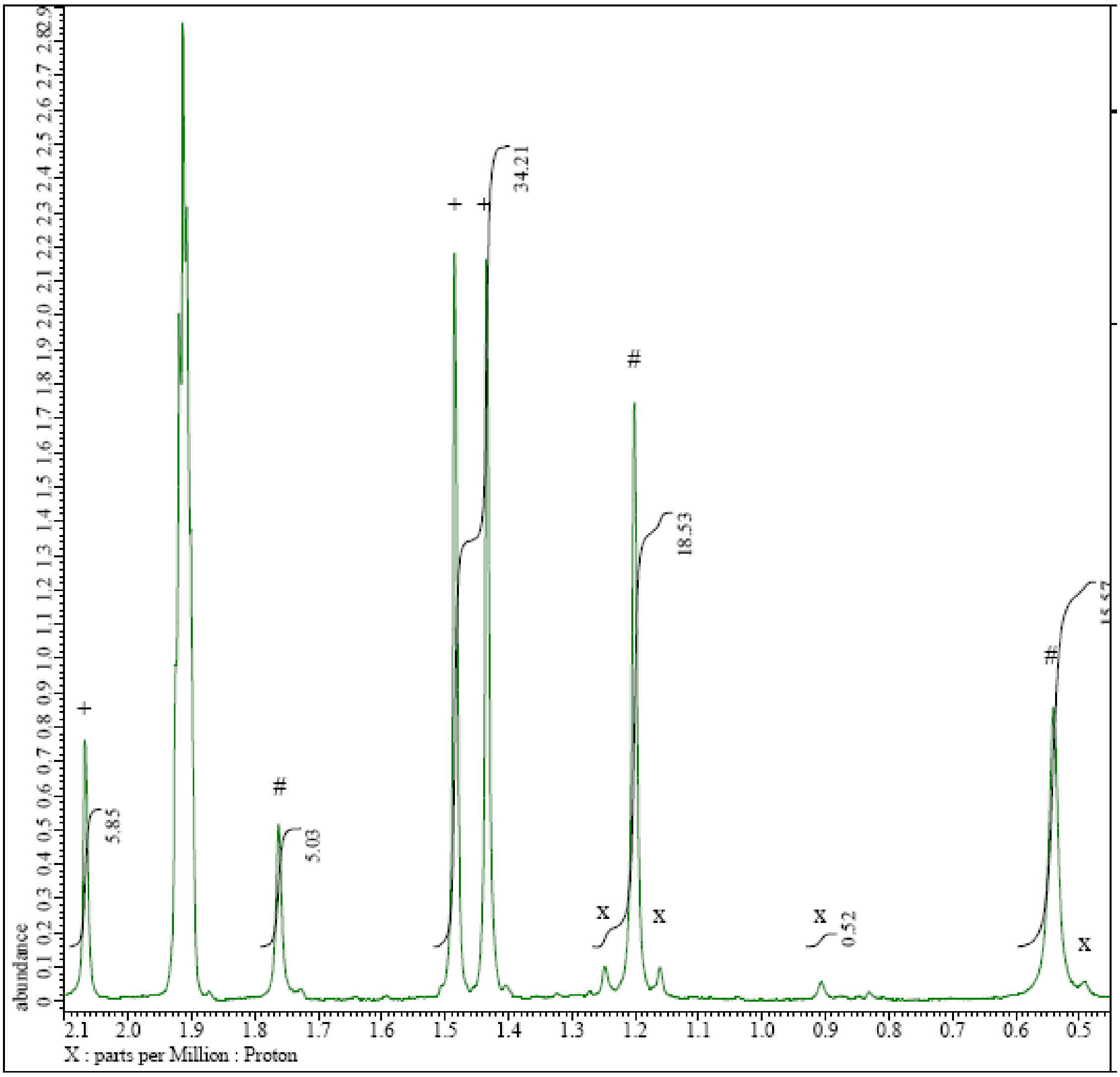

Figure 35. Aliphatic region of the ^{1}H NMR spectrum of the 1:2 bis-complex of ligand **10** with $Y(NO_3)_3$ in CD_3CN after ligand **8** (1 equivalent) had been added (Assignments: # = 1:2 bis-complex of **10**, + = free uncomplexed ligand **8**, x = heteroleptic 1:2 bis-complex). Peak at 1.91 ppm is due to solvent.

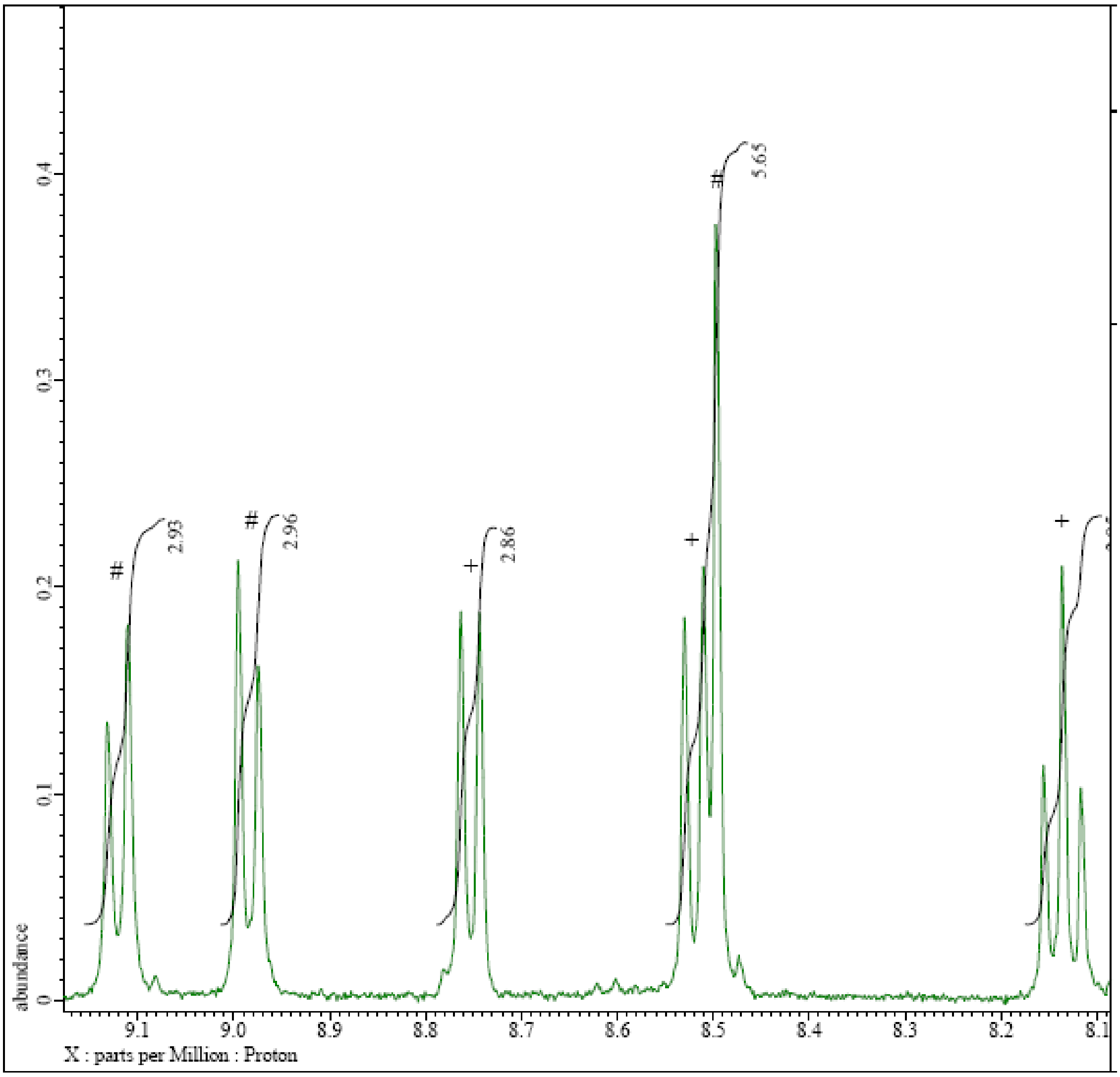

Figure 36. Aromatic region of the ^{1}H NMR spectrum of the 1:2 bis-complex of ligand **10** with $Y(NO_3)_3$ in CD_3CN after ligand **8** (1 equivalent) had been added (Assignments: # = 1:2 bis-complex of **10**, + = free uncomplexed ligand **8**).

6: TRLFS Measurements

6.1 Complexation Kinetics

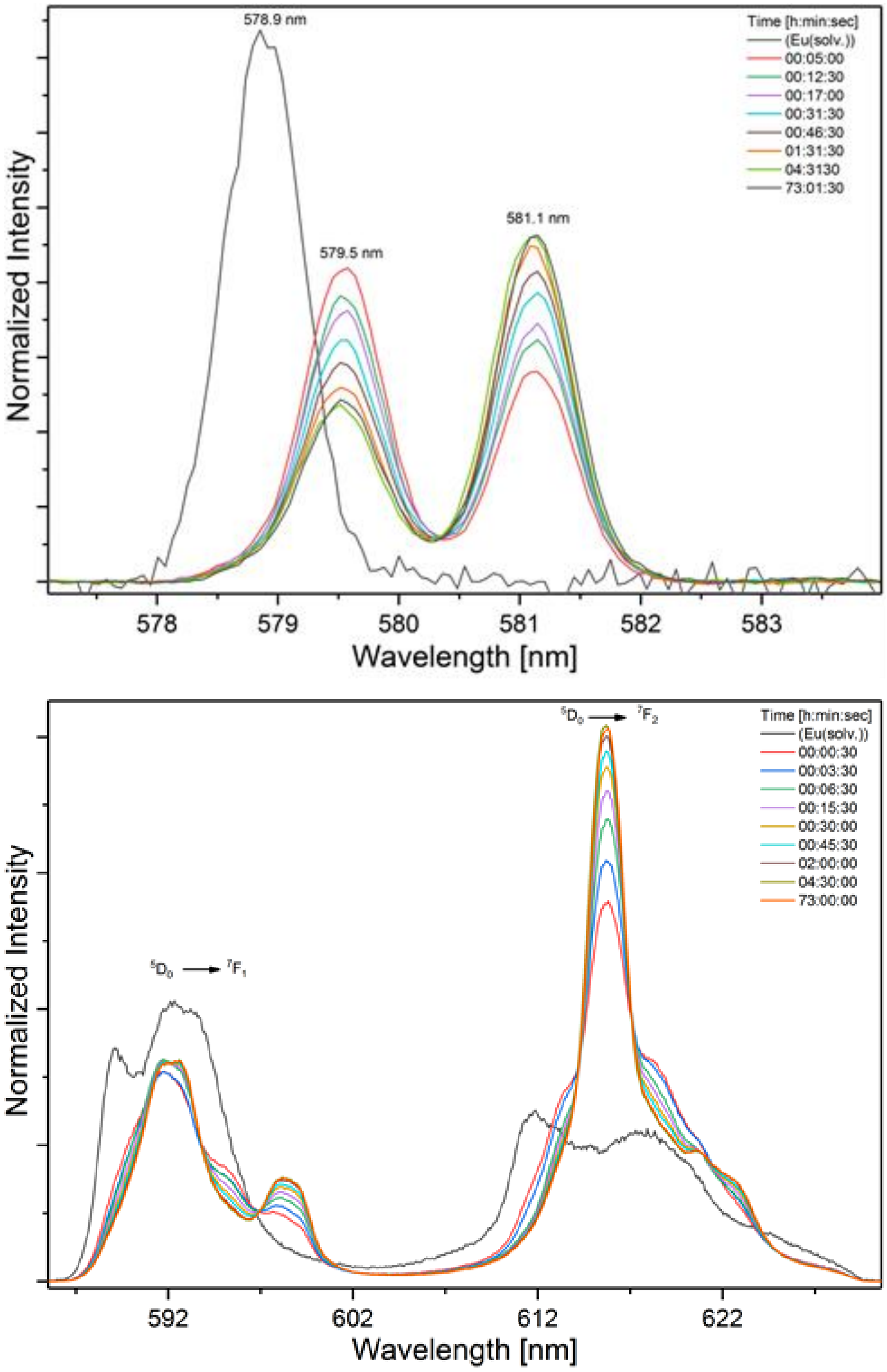

Figure 37. Normalized Eu(III) fluorescence spectra of the $^5D_0 \rightarrow {}^7F_n$ transitions (Top: n = 0; bottom: n = 1,2) as a function of time after addition of the ligand **10** in MeOH + 1.5 vol.% H_2O (c(H^+) = 91.2 mM; c(**10**) = 4.96 × 10^{-7} M; c(Eu(III)) = 7.68 × 10^{-6} M).

6.2 Complexation of Cm(III) and Eu(III) with 10

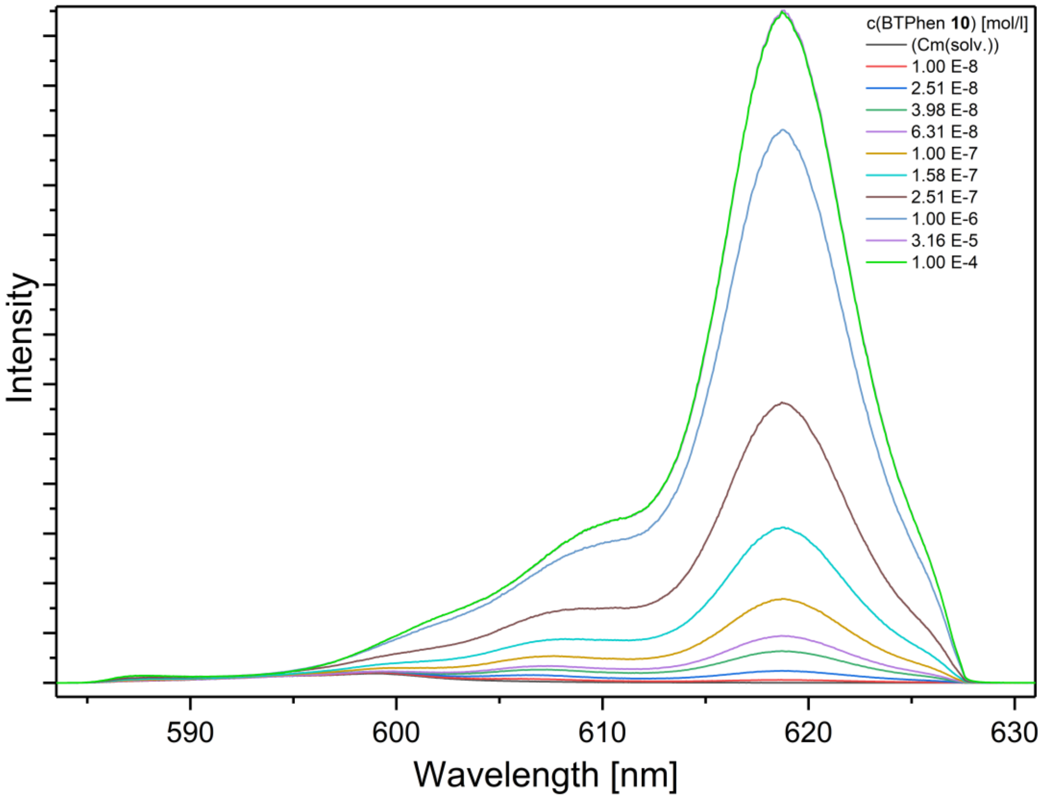

Figure 38. Cm(III) fluorescence spectra as a function of the ligand **10** concentration in MeOH + 1.5 vol.% H_2O ($c(H^+)$ = 91.2 mM; c(Cm(III)) = 4.69×10^{-8} M).

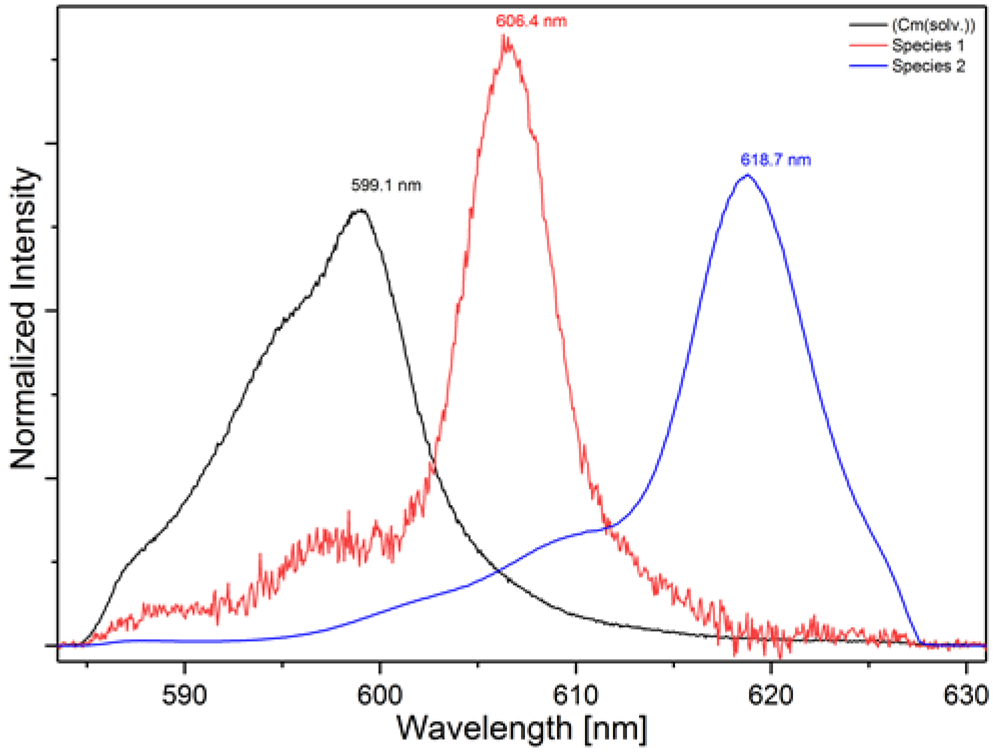

Figure 39. Normalized Cm(III) fluorescence spectra of the single component spectra of the Cm(III) solvent species and the Cm(III)-ligand **10** complex species in MeOH + 1.5 vol.% H_2O ($c(H^+)$ = 91.2 mM).

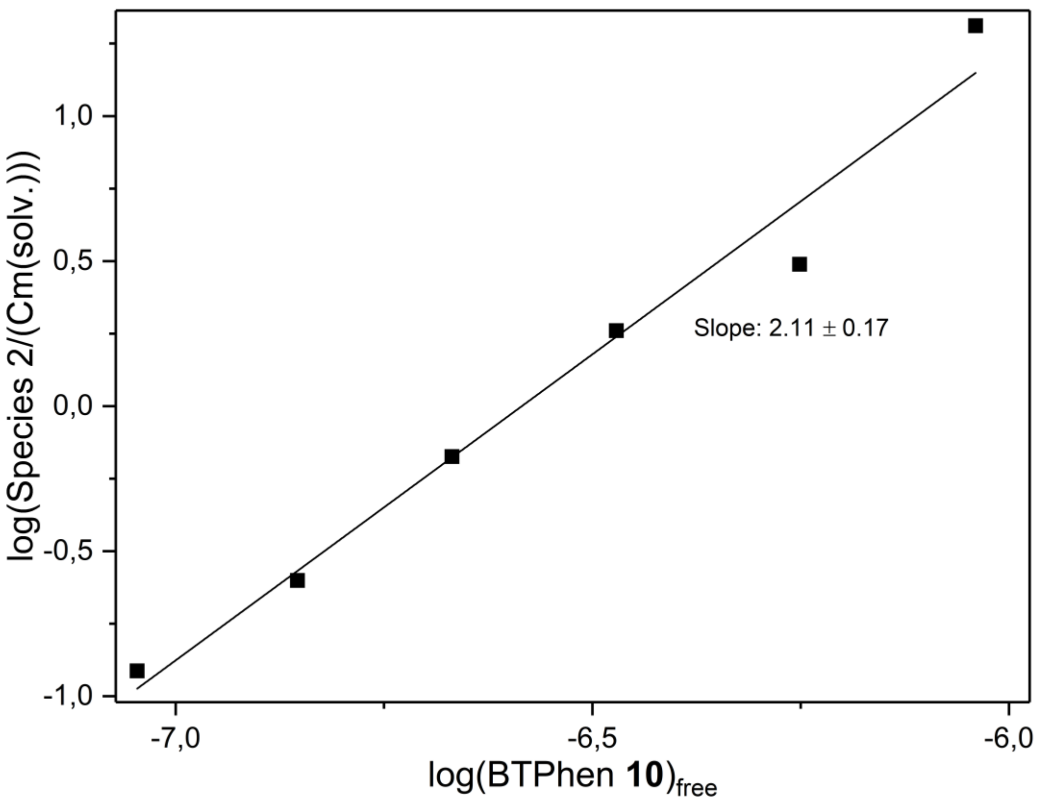

Figure 40. Double logarithmic plot of the concentration ratio c(Species 1)/c(Cm(solv.)) vs. the free ligand **10** concentration.

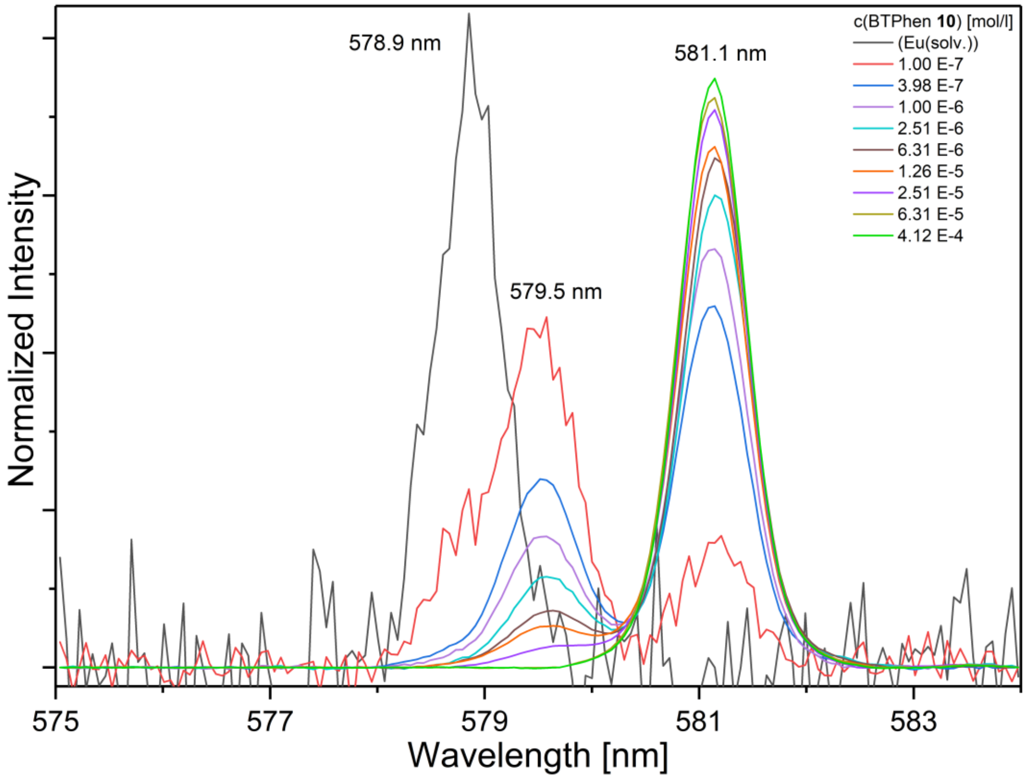

Figure 41. Normalized Eu(III) fluorescence spectra of the $^5D_0 \rightarrow {}^7F_0$ transition as a function of the ligand **10** concentration in MeOH + 1.5 vol.% H_2O ($c(H^+)$ = 91.2 mM; c(Cm(III)) = 4.69×10^{-8} M).

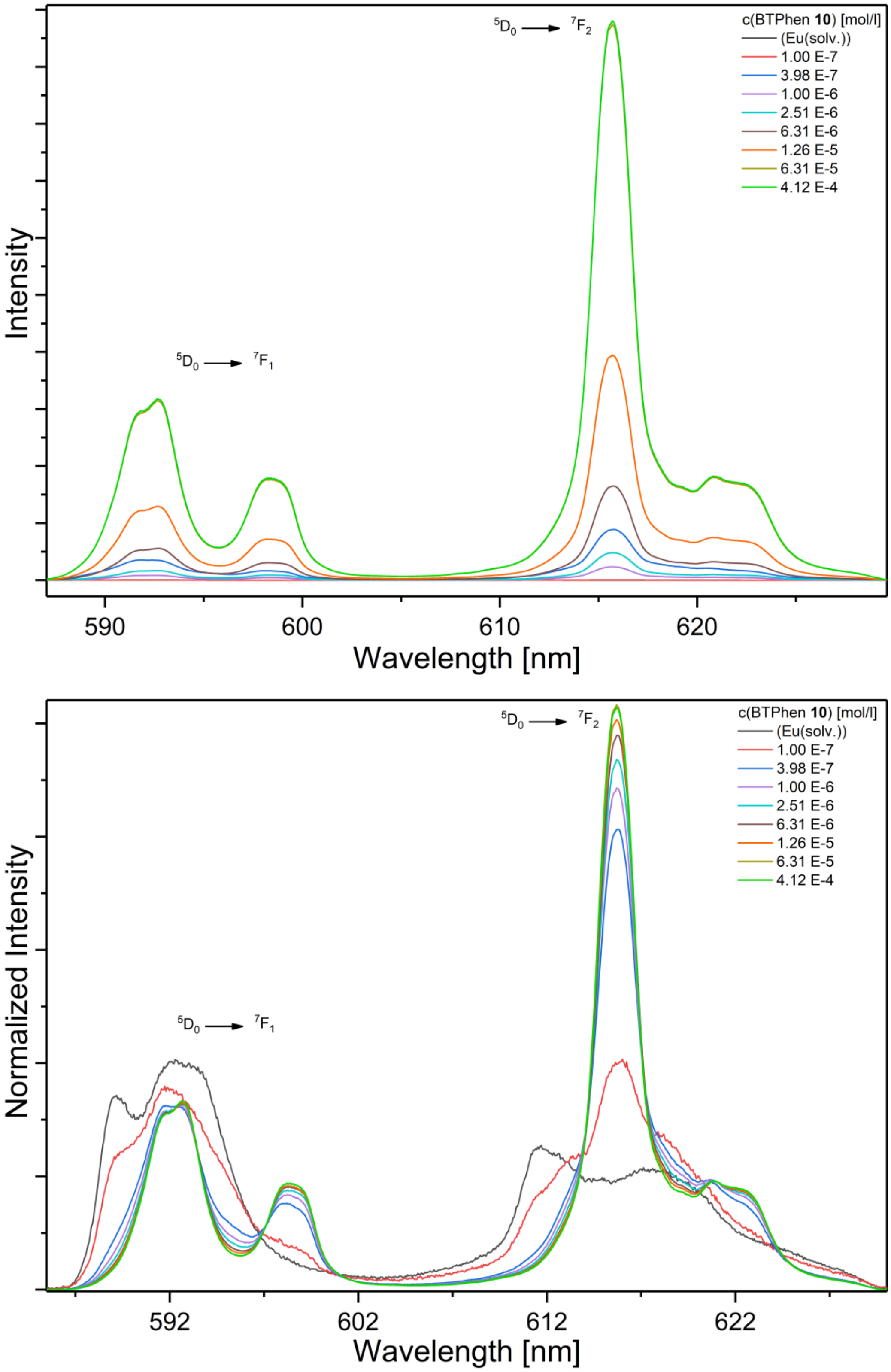

Figure 42. Normalized Eu(III) fluorescence spectra of the $^5D_0 \rightarrow {}^7F_1$ and $^5D_0 \rightarrow {}^7F_2$ transitions as a function of the ligand **10** concentration in MeOH + 1.5 vol.% H_2O (c(H^+) = 91.2 mM; c(Cm(III)) = 4.69×10^{-8} M).

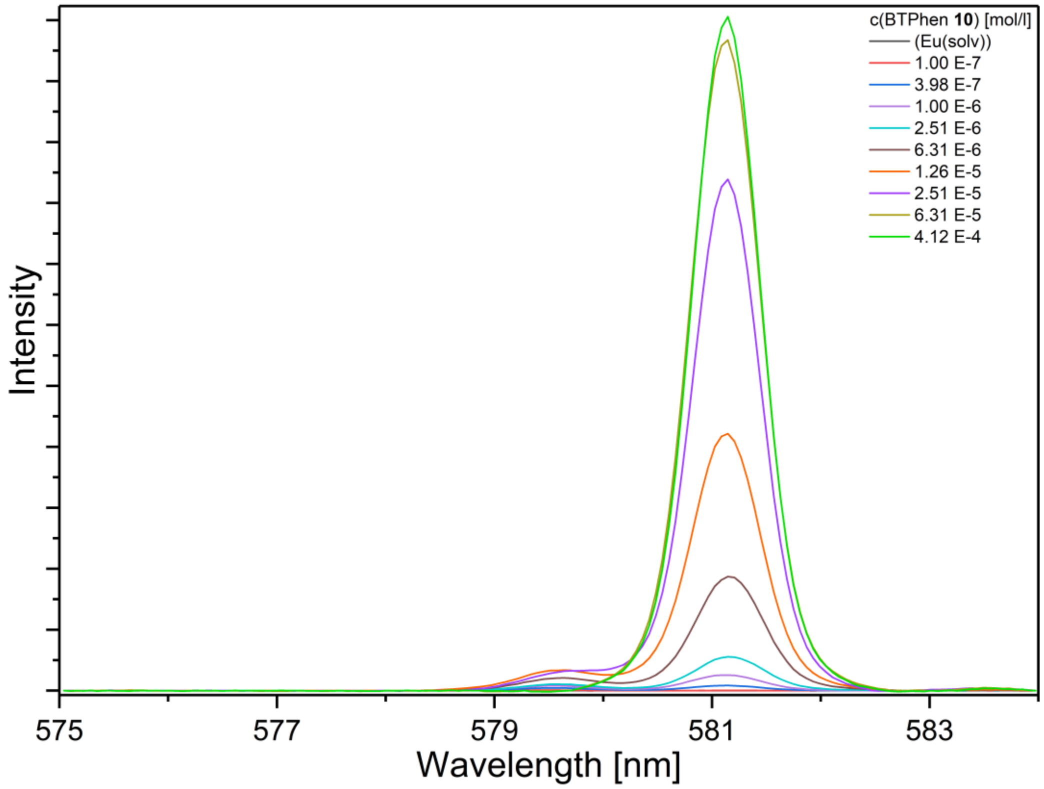

Figure 43. Eu(III) fluorescence spectra of the $^5D_0 \rightarrow {}^7F_0$ transition as a function of the ligand **10** concentration in MeOH + 1.5 vol.% H_2O (c(H^+) = 91.2 mM; c(Cm(III)) = 4.69 × 10^{-8} M).

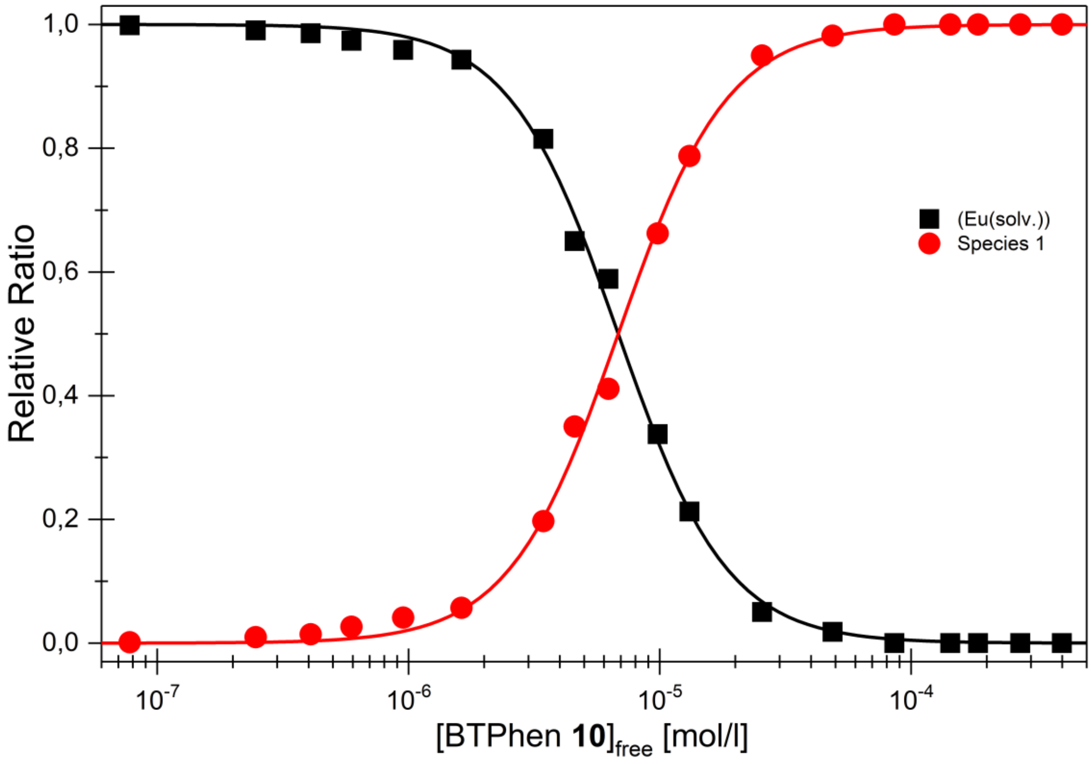

Figure 44. Relative ratio of Eu(solv.) and species 1 as a function of the ligand **10** concentration. Symbols represent experimental data whereas lines denote calculations.

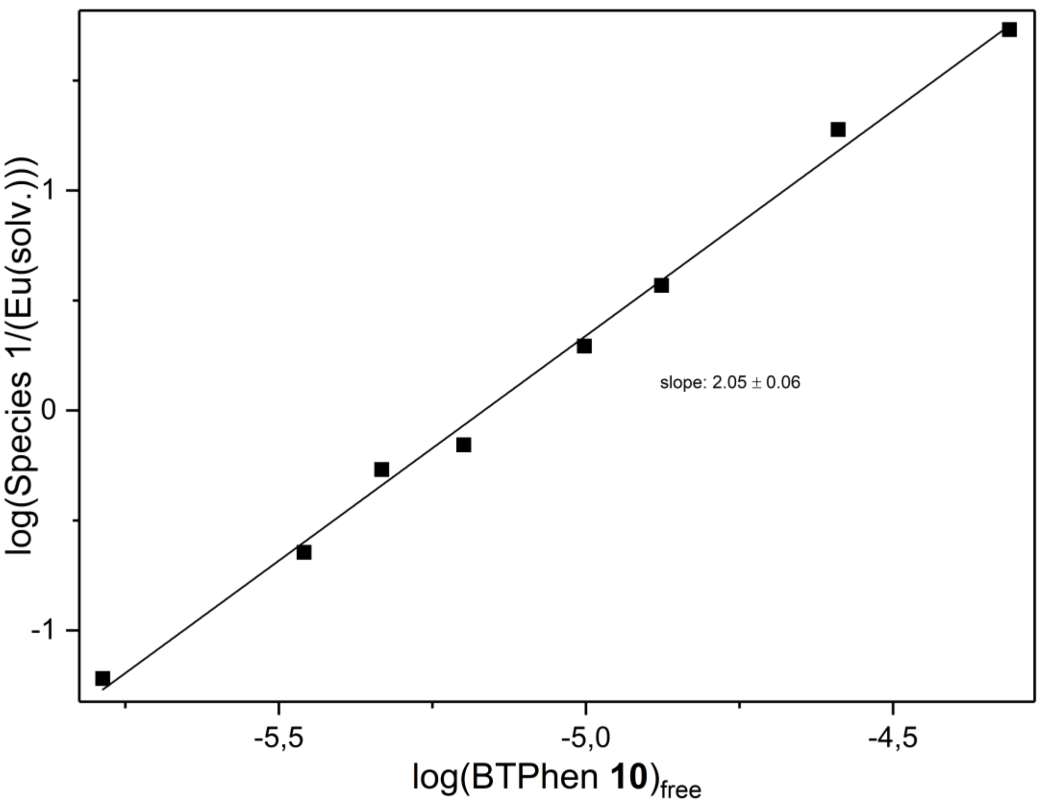

Figure 45. Double logarithmic plot of the concentration ratio c(Species 1)/c(Eu(solv.)) vs. the free ligand **10** concentration.

6.3 Comparison of Mono- and Biphasic Experiments

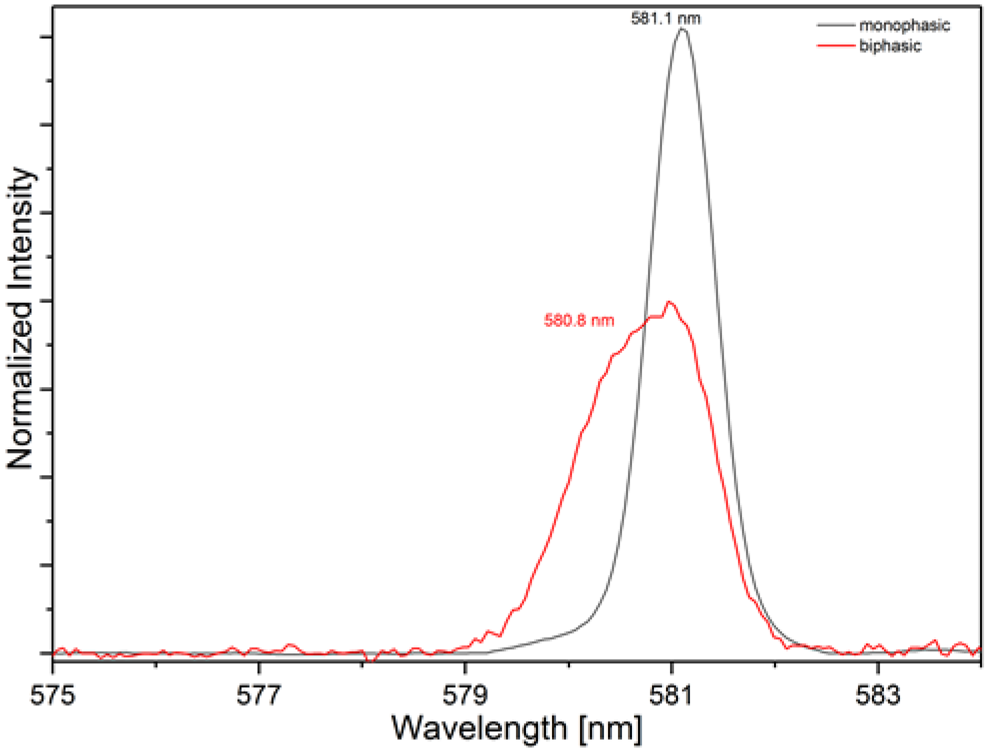

Figure 46. Comparison of the Eu(III) fluorescence emission spectra of (black) the $[Eu(\mathbf{10})_2]^{3+}$ complex in a monophasic batch experiment and (red) the formed complex in the organic phase after extraction. $^5D_0 \rightarrow {}^7F_0$ transition.

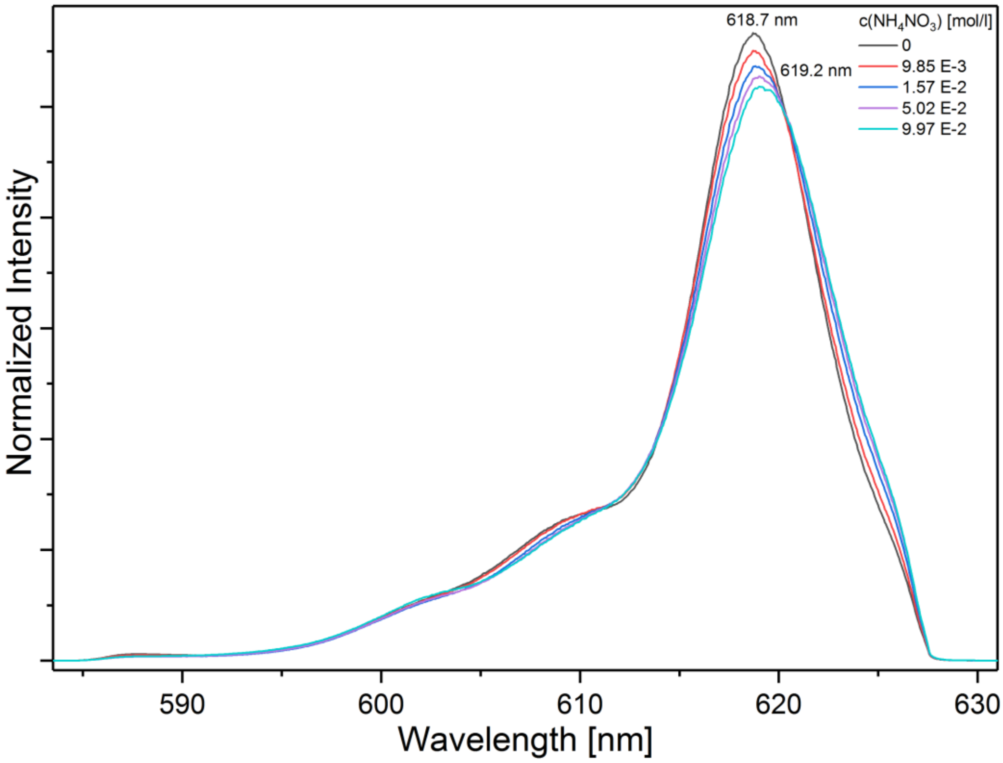

Figure 47. Normalized Cm(III) fluorescence spectra as a function of the NH_4NO_3 concentration in MeOH + 1.5 vol.% H_2O ($c(H^+)$ = 91.2 mM; $c(Cm(III))_{ini} = 4.64 \times 10^{-8}$ M; $c(\mathbf{10})_{ini} = 1.00 \times 10^{-5}$ M).

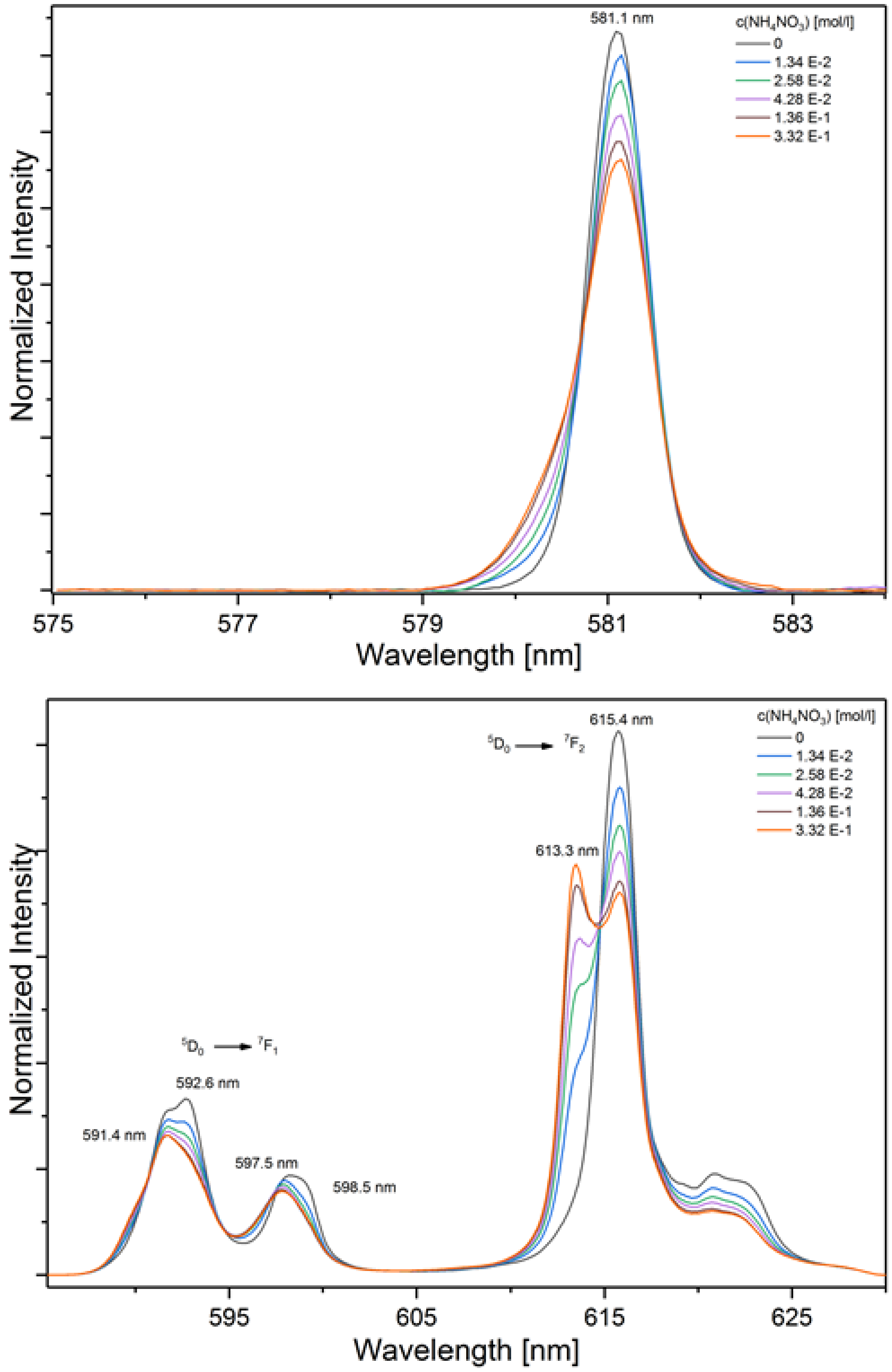

Figure 48. Normalized Eu(III) fluorescence spectra of the $^5D_0 \rightarrow {}^7F_n$ transitions (Top: n = 0; bottom: n = 1,2) as a function of the NH_4NO_3 concentration in MeOH + 1.5 vol.% H_2O ($c(H^+)$ = 91.2 mM; $c(Eu(III))_{ini} = 6.49 \times 10^{-6}$ M; $c(\mathbf{10})_{ini} = 1.58 \times 10^{-4}$ M).

7: DFT Calculations

Ligands **3** and **10** were optimised in the gas phase in the three conformations shown in Figure 49, and their optimised energies are given in Table 19. Single point energies of the gas-phase optimised ligands in an acetonitrile solvent field are given in Table 20, demonstrating that conformation b is shown to be the most stable for both **3** and **10**. This conformation is also shown to be the most stable for **3** and **10** in a 1-octanol solvent field, for which the energies are given in Table 21. Structures of the complexes $[Am(\mathbf{3})_2(NO_3)]^{2+}$ and $[Am(\mathbf{10})_2(NO_3)]^{2+}$ optimised in the gas phase are shown in Figure 50.

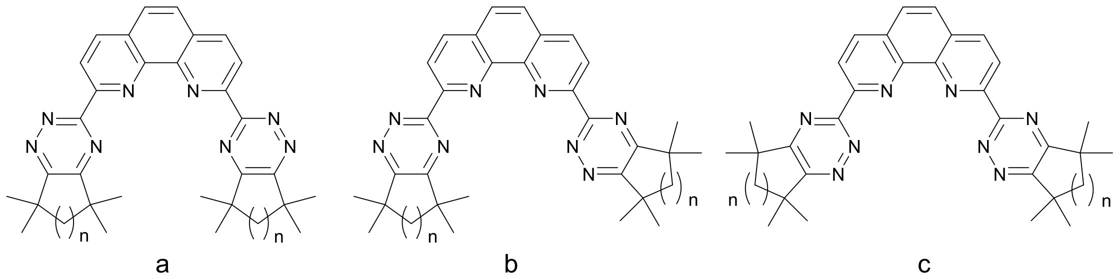

Figure 49. Conformations of ligands **3** and **10** used for DFT optimisations (n = 2 for ligand **3**, n = 1 for ligand **10**).

The binding energy (*E*) for one mole of each ligand (**L**), assumed to be in the most stable conformation b in both cases, was calculated according to the following theoretical reaction schemes:

$$[AmNO_3]^{2+} + 2\mathbf{L} \longrightarrow [Am\mathbf{L}_2NO_3]^{2+}$$

$$E = \frac{E([Am\mathbf{L}_2NO_3]^{2+}) - \left(E([AmNO_3]^{2+}) + 2\,E(\mathbf{L})\right)}{2}$$

The absolute and relative calculated binding energies in the gas phase (no solvent field), in an acetonitrile solvent field, and in a 1-octanol solvent field are given in Table 22.

Table 19. Calculated absolute and relative energies of free ligands **3** and **10**, and their Am(III) complexes in the gas phase.

Compound	Conformation	Absolute energy/Ha	Relative energy/kJ mol^{-1}
**3**	a	−1757.051877	0.000
**3**	b	−1757.051286	1.552
**3**	c	−1757.046546	13.998
**10**	a	−1678.392511	0.000
**10**	b	−1678.391110	3.680
**10**	c	−1678.386022	17.039
[Am(**3**)$_2$(NO_3)]$^{2+}$	–	−4389.897671	–
[Am(**10**)$_2$(NO_3)]$^{2+}$	–	−4232.580303	–
[Am(NO_3)]$^{2+}$	–	−875.237455	–

Table 20. Calculated absolute and relative energies of free ligands **3** and **10**, and their Am(III) complexes in an acetonitrile solvent field.

Compound	Conformation	Absolute energy/Ha	Relative energy/kJ mol^{-1}
**3**	a	−1757.101870	5.561
**3**	b	−1757.103988	0.000
**3**	c	−1757.103375	1.609
**10**	a	−1678.444267	2.321
**10**	b	−1678.445151	0.000
**10**	c	−1678.444354	2.093
[Am(**3**)$_2$(NO_3)]$^{2+}$	–	−4390.116995	–
[Am(**10**)$_2$(NO_3)]$^{2+}$	–	−4232.800658	–
[Am(NO_3)]$^{2+}$	–	−875.8553812	–

Table 21. Calculated absolute and relative energies of free ligands **3** and **10**, and their Am(III) complexes in a 1-octanol solvent field.

Compound	Conformation	Absolute energy/Ha	Relative energy/kJ mol^{-1}
**3**	a	−1757.091806	2.664
**3**	b	−1757.092821	0.000
**3**	c	−1757.091644	3.090
**10**	a	−1678.433895	0.760
**10**	b	−1678.434184	0.000
**10**	c	−1678.432471	4.499
$[Am(\mathbf{3})_2(NO_3)]^{2+}$	–	−4390.094298	–
$[Am(\mathbf{10})_2(NO_3)]^{2+}$	–	−4232.778301	–
$[Am(NO_3)]^{2+}$	–	−875.823402	–

Table 22. Calculated binding energies for ligands **3** and **10** for molecules in the gas phase, in an acetonitrile solvent field, and in a 1-octanol solvent field.

Ligand	Solvent	Absolute binding energy/Ha	Relative binding energy/kJ mol^{-1}
**3**	–	−0.278231	1.791
**3**	acetonitrile	−0.026819	1.755
**3**	1-octanol	−0.042627	1.676
**10**	–	−0.278913	0.000
**10**	acetonitrile	−0.027487	0.000
**10**	1-octanol	−0.043265	0.000

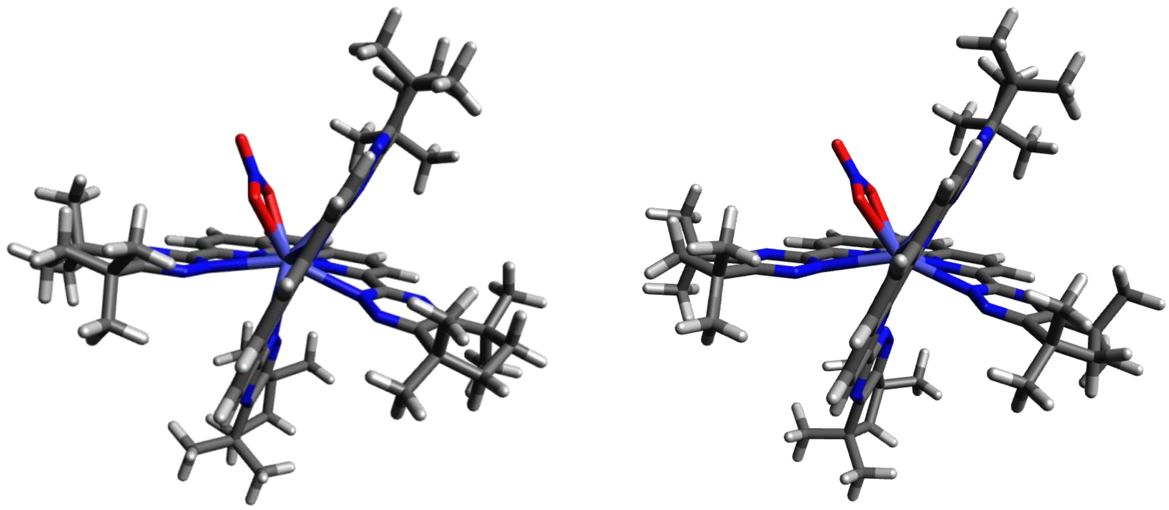

Figure 50. Gas-phase optimised structures of $[Am(\mathbf{3})_2(NO_3)]^{2+}$ (left) and $[Am(\mathbf{10})_2(NO_3)]^{2+}$ (right).

8: References

1. F. W. Lewis, L. M. Harwood, M. J. Hudson, A. Geist, V. N. Kozhevnikov, P. Distler, J. John, *Chem. Sci.* **2015**, *6*, 4812–4821.
2. G. R. Pabst, O. C. Pfuller, J. Sauer, *Tetrahedron* **1999**, *55*, 5047–5066.
3. (a) F. W. Lewis, L. M. Harwood, M. J. Hudson, M. G. B. Drew, J. F. Desreux, G. Vidick. N. Bouslimani, G. Modolo, A. Wilden, M. Sypula, T.-H. Vu, J.-P. Simonin, *J. Am. Chem. Soc.* **2011**, *133*, 13093–13102. (b) F. W. Lewis, L. M. Harwood, M. J. Hudson, M. G. B. Drew, A. Wilden, M. Sypula, G. Modolo, T.-H. Vu, J.-P. Simonin, G. Vidick, N. Bouslimani, J. F. Desreux, *Proc. Chem.* **2012**, *7*, 231–238.
4. (a) G. E. Gream, J. C. Paice, C. C. R. Ramsay, *Aust. J. Chem.* **1969**, *22*, 1229–1247. (b) I. K. Korobitsyna, V. A. Nikolaev, *J. Org. Chem. USSR* **1976**, *12*, 1245–1251. (c) P. L. Verheidt, C. Kruk, H. Cerfontain, *Rec. Trav. Chim. Pays Bas* **1982**, *101*, 85–87.
5. R. C. Clark, J. S. Reid, *Acta Crystallogr. Sect. A: Found. Crystallogr.* **1995**, *51*, 887–897.
6. CrysAlisPro, Rigaku Oxford Diffraction, Tokyo, Japan.
7. G. M. Sheldrick, *Acta Crystallogr. Sect. A: Found. Crystallogr.* **2015**, *71*, 3–8.
8. G. M. Sheldrick, *Acta Crystallogr. Sect. A: Found. Crystallogr.* **2008**, *64*, 112–122.
9. O. V. Dolomanov, L. J. Bourhis, R. J. Gildea, J. A. K. Howard, H. Puschmann, *J. Appl. Cryst.* **2009**, *42*, 339–341.
10. M. J. Frisch, G. W. Trucks, H. B. Schlegel, G. E. Scuseria, M. A. Robb, J. R. Cheeseman, G. Scalmani, V. Barone, B. Mennucci, G. A. Petersson et al. Gaussian 09, Revision C.01. Gaussian Inc., Wallingford CT **2009**.
11. A. D. Becke, *J. Chem. Phys.* **1993**, *98*, 5648–5652.
12. C. T. Lee, W. T. Yang, R. G. Parr, *Phys. Rev. B* **1988**, *37*, 785–789.
13. A. Schäfer, H. Horn, R. Ahlrichs, *J. Chem. Phys.* **1992**, *97*, 2571–2577.
14. K. Eichkorn, F. Weigend, O. Treutler, R. Ahlrichs, *Theor. Chem. Acta* **1997**, *97*, 119–124.
15. W. Küchle, M. Dolg, H. Stoll, H. Preuss, *J. Chem. Phys.* **1994**, *100*, 7535–7542.
16. X. Cao, M. Dolg, H. Stoll, *J. Chem. Phys.* **2002**, *118*, 487–496.
17. A. Schäfer, C. Huber, R. Ahlrichs, *J. Chem. Phys.* **1994**, *100*, 5829–5835.
18. M. Sundararajan, V. Sinha, T. Bandyopadhyay, S. K. Ghosh, *J. Phys. Chem. A* **2012**, *116*, 4388–4395.

19. A. Bhattacharyya, M. Mohapatra, P. K. Mohapatra, T. Gadly, S. K. Ghosh, D. Manna, T. K. Ghanty, N. Rawat, B. S. Tomar, *Eur. J. Inorg. Chem.* **2017**, 820–828.
20. D. Manna, S. Mula, A. Bhattacharyya, S. Chattopadhyay, T. K. Ghanty, *Dalton Trans.* **2015**, *44*, 1332–1340.
21. A. V. Marenich, C. J. Cramer, D. G. Truhlar, *J. Phys. Chem. B* **2009**, *113*, 6378–6396.
